# Sensitivity of habitat network models to changes in maximum dispersal distance

**DOI:** 10.1371/journal.pone.0293966

**Published:** 2023-11-06

**Authors:** Damian O. Ortiz-Rodríguez, Antoine Guisan, Maarten J. Van Strien

**Affiliations:** 1 Planning of Landscape and Urban Systems (PLUS), Institute for Spatial and Landscape Planning, ETH Zürich, Zürich, Switzerland; 2 WSL Swiss Federal Research Institute, Birmensdorf, Switzerland; 3 Department of Environmental Systems Science, ETH Zürich, Zürich, Switzerland; 4 Department of Ecology and Evolution, University of Lausanne, Lausanne, Switzerland; 5 Institute of Earth Surface Dynamics, University of Lausanne, Lausanne, Switzerland; Southeastern Louisiana University, UNITED STATES

## Abstract

Predicting the presence or absence (occurrence-state) of species in a certain area is highly important for conservation. Occurrence-state can be assessed by network models that take suitable habitat patches as nodes, connected by potential dispersal of species. To determine connections, a connectivity threshold is set at the species’ maximum dispersal distance. However, this requires field observations prone to underestimation, so for most animal species there are no trustable maximum dispersal distance estimations. This limits the development of accurate network models to predict species occurrence-state. In this study, we performed a sensitivity analysis of the performance of network models to different settings of maximum dispersal distance. Our approach, applied on six amphibian species in Switzerland, used habitat suitability modelling to define habitat patches, which were linked within a dispersal distance threshold to form habitat networks. We used network topological measures, patch suitability, and patch size to explain species occurrence-state in habitat patches through boosted regression trees. These modelling steps were repeated on each species for different maximum dispersal distances, including a species-specific value from literature. We evaluated mainly the predictive performance and predictor importance among the network models. We found that predictive performance had a positive relation with the distance threshold, and that almost none of the species-specific values from literature yielded the best performance across tested thresholds. With increasing dispersal distance, the importance of the habitat-quality-related variable decreased, whereas that of the topology-related predictors increased. We conclude that the sensitivity of these models to the dispersal distance parameter stems from the very different topologies formed with different movement assumptions. Most reported maximum dispersal distances are underestimated, presumably due to leptokurtic dispersal distribution. Our results imply that caution should be taken when selecting a dispersal distance threshold, considering higher values than those derived from field reports, to account for long-distance dispersers.

## Introduction

The distance across which individuals of a species can physically move and disperse is a crucial element to consider in biodiversity conservation, as it is an important determinant of the probability that an organism is able to reach and occupy other areas. An effective way to study patterns of spatial distribution of animal (meta)populations [1] and movement across landscapes is through the conceptual framework of habitat mosaics [2, 3], which are spatial representations of suitable habitat patches in an unsuitable (or less suitable) matrix [4]. The movement of animals through the matrix allows them to reach new suitable patches after habitat destruction or resource depletion (e.g. food or water). Colonization of new suitable patches is also important for the maintenance of gene flow among populations [5, 6]. There can be multiple factors, such as specific insurmountable landscape features (e.g. barriers), that make it difficult for a species to reach other suitable patches [7, 8]. However, even without significant resistance to movement from the matrix, physical, morphological or behavioral traits can also limit the dispersal abilities of organisms, allowing them to only cover a certain maximum distance [5, 9]. Due to intraspecific variation, the dispersal distance is generally not a fixed value, and aggregated for individuals of a population it can be seen as a probability distribution (i.e. dispersal kernel) in which the maximum dispersal distance corresponds to the distant end of the tail [10]. The probabilities of movement and dispersal thus directly affect the probability of finding a species in an environmentally-suitable habitat patch. Thus, the persistence of a population in a habitat patch not only depends on factors related to habitat quality and size, but also on its connectivity to surrounding patches [11, 12], and generally to the structure (topology) of the habitat network it forms a part of [3, 13].

There is currently a wealth of network modelling applications in landscape ecology and conservation. These include studies on the impact of transport infrastructure on the genetic structure of a metapopulation [14], prioritization of areas for conservation [15, 16], demonstrations of the relevance theoretical principles from islands biogeography [17], and assessments of robustness against habitat loss [18]. In a habitat network, the nodes represent the habitat patches, and the edges or links represent potential movement of organisms between them [3, 19]. In networks in which not all of the patches are connected to all other patches (i.e. the network is not fully-connected), the network can be seen as split into several “components” [19]. Inside a network component, each patch can be reached directly or indirectly from all other patches. Single patches that are not connected to any other are themselves also separate network components [20]. Since several network indices that describe the position or importance of a patch in the network rely on calculating connections with an extended vicinity (e.g. neighborhood [21]) or all the other patches in the network (e.g. probability of connectivity [22], betweenness centrality [23]), their values are limited to the nodes that form a particular component (but note that this depends on the algorithm settings used to calculate them). Therefore, it is relevant to know how the network is organized and whether and how much it is fragmented.

Determining whether two patches are connected by an edge needs to take into consideration if the dispersal abilities of species allow movement between the pair of patches [24]. Given this, habitat networks need to specify a geographical distance over which no edges are formed, which is dependent on such dispersal ability. Therefore, data on maximum dispersal distances is needed to parametrize network models. Available data on maximum dispersal distances consists of estimates based on what has been observed in the field, but getting reliable estimates is very difficult for two reasons. First, dispersal distance estimates from genetic analyses in principle can only detect “effective dispersal”, which is when the migrant organisms successfully reproduce [25, 26] (but see Manel, Gaggiotti [27]). Second, these techniques often rely on unrealistic assumptions, such as complete genotyping of the parent pools or Hardy-Weinberg equilibrium in the population [26, 28]. This makes the determination of the presence of migrants in a certain patch by these means often unreliable [26]. Other possible methods to determine maximum dispersal distances are radio or GPS tracking [29, 30]. However, especially for small organisms, these methods have limitations related to the physical placement of transmitters and the low battery life of small devices [31]. Harmonic direction finders solve those problems [32, 33], but they have a very short detection range and lack signal differentiation for specific individuals [31, 34], which would require other methods of field marking and identification to measure maximum dispersal distances. For mark-recapture studies (and indeed for all other methods), the drawback is that there are uncommon long-distance dispersal events which are difficult to detect [35, 36], which often causes the maximum dispersal distances of species to be underestimated [37, 38]. In short, despite the importance of knowing the maximum dispersal distance to construct habitat network models, there is great ambiguity in estimating this distance in the field.

The application of advanced modelling techniques to conservation is especially relevant and urgent in the case of amphibians, as it is globally the most endangered and sharply declining class of vertebrates [39]. Fortunately, there are already efforts relying on network modelling to assess the presence and distribution of amphibians in habitat patches (e.g. [40, 41]). However, for the reasons mentioned above, also for amphibians the estimation of the maximum dispersal distance is problematic. In a comprehensive review of amphibian dispersal by Smith and Green [42], whose findings were revisited by Covarrubias et al. [50], there is a reported mean maximum dispersal distance for anurans of 2.02 km. Smith and Green mention that this is double the distance (i.e. 1 km) over which, according to previous reports, “amphibian populations would be isolated from dispersal” [42: 113]. In the same work, it is further reported that 44% of the amphibian species from the studies they compiled move no farther than 400 m, while 7% of the anurans disperse to more than 10 km. Importantly, these authors also found that probability distribution of dispersal distances of amphibians in many cases follows an inverse power law, or more generally, a leptokurtic distribution (most individuals moving over a relatively short distance, but with a considerable amount of individuals moving over large distances). Smith and Green [42: 119] also concluded that “monitoring larger areas in the future will result in the discovery of longer distance movements”. Despite the importance of knowing the maximum dispersal distance to construct habitat network models, there is thus great uncertainty in estimating this distance in nature. This raises the question how sensitive the results of network modelling approaches are to the choice of maximum dispersal distance.

In this study, we explored the effect of maximum dispersal distance on the predictive power of network-based species occurrence-state (presence or absence) models. We applied the original network modelling suite presented in Ortiz-Rodríguez et al. [41] to six amphibian species of conservation importance in the Swiss Plateau. In brief, this modelling suite uses Habitat Suitability Modelling (HSM; [43]) as the basis for delineating the habitat patches that form the nodes of a network model. As standard site-occupancy models are inadequate for non-systematically sampled data, the occurrence-state (i.e. the presence or absence of a species) in these habitat patches is determined by assessing sampling intensity, and the network models are subsequently used to fit the occurrence-state to predictor variables including network topology measures [41]. In addition to fitting models with edge formation delimited by species-specific (mean) maximum dispersal distances taken from the literature, we also generated a series of networks with a uniform set of maximum dispersal distances. This resulted in a large number of different habitat networks. We performed an extensive sensitivity analysis on these habitat networks, comparing them based on their predictive performance, their structure, and the importance of different predictor variables. We discussed the implications of our results for amphibian dispersal and the interpretation of habitat network models overall.

## Methods

### Species and study area

We used data from six amphibian species, which are either the most endangered in Switzerland (*Alytes obstetricans*, *Bombina variegata*, *Epidalea calamita* and *Hyla arborea*) or are problematic because they are part of a genetic complex with a history of invasion and displacement (*Pelophylax ridibundus* and *P*. *lessonae* / *P*. *esculentus*, nearly indistinguishable in the field, henceforth referred to as *Pelophylax lessonae* agg [44–46]). We focused on the Swiss Plateau, a densely populated [47] European landscape with many anthropogenic pressures. We extracted species presence data from an aggregated database of geopositioned records of 13 amphibian species observed at water bodies between 2006 and 2015 by different monitoring projects and observers (therefore, not systematically sampled), and provided by InfoSpecies KARCH (www.karch.ch). As we only worked with such readily available data, and did not handle directly any animals, no approval from any animal research ethics committee was necessary.

### HSM and patch delineation

For each species, we used a selection of 20 environmental predictors (S1 Appendix) to do an ensemble HSM (combining GLM, MaxEnt and Random Forest as modelling techniques) within the R-package Biomod2 [48], using default package settings. We binarized the continuous suitability maps by the point of the ROC curve that minimized the difference between sensitivity and specificity. On the obtained binary suitability maps (suitable vs. non-suitable) we applied an environmental mask consisting of stationary water bodies, amphibian spawning sites and reported presences of any amphibian in the original database. Each area in the mask that was classified as suitable was considered a habitat patch. For each of these patches, we calculated the patch area as well as the Habitat Suitability Index (HSI), defined as the mean value of habitat suitability in the continuous suitability map output by the HSM. The patch area and HSI were then used as predictor variables in the network boosted regression trees (BRT) models (see below).

### Network generation

As in Ortiz-Rodríguez et al. [41], we defined the edges with a least cumulative cost algorithm that, in the present case, operated over a uniform resistance surface, which meant that the costs equaled approximately the interpatch Euclidean distances. The algorithm drew an edge between pairs of patches if the cost was below a certain threshold, which corresponded to a chosen maximum dispersal distance. The conversion of cost distances to dispersal probabilities was performed according to the p2p function of the R-package PopGenReport [49]:

$$cost\;=log\;\left(prob\right)/log\left(p\right)*d0$$ (1)

in which *cost* is the cost–value associated with a certain probability *prob*, *prob* is the probability of dispersal between patches, and *d0* is the dispersal distance of a proportion *p* of individuals. We set *p* = 0.5 so that *d0* equaled the median dispersal distance. The dispersal probability threshold beyond which no edges were drawn was set to 0.0001. By adjusting the d0 term, we were able to simulate a range of maximum dispersal distances (see below). This algorithm was built to accommodate the usage of different cost surfaces, which makes these networks more directly comparable to networks generated with different cost surface definitions.

### Dispersal distances included

For every species, we developed networks with their species-specific maximum dispersal distance reported in the literature (Table 1). To be consistent with Ortiz-Rodríguez et al. [41], we used for *H*. *arborea* a maximum dispersal distance value of 2658 m, which was only 258 m higher than the reported value in Smith and Green [42]. Considering the dispersal distances reported in Smith and Green [42] and Covarrubias et al. [50], we also built networks with maximum dispersal distances for the extremes of 300 m and 10 km, the distance previously considered as limit for dispersal events (1 km), and the reported mean maximum dispersal distance for anurans (2 km). We also included a maximum dispersal distance of 4 km as another fixed value considering that in the review of Cayuela et al. [51] the maximum dispersal distance of amphibians was generally given as 3698 ± 6256 m and as 4506 ± 7269 m for anurans only. In addition, out of our studied species, one (*Bombina variegata*) had exactly 4 km as species-specific reported maximum dispersal distance [52], while that of another species (*Epidalea calamita*) was close to that value. We additionally expanded the exploration of maximum dispersal distances for all species with fixed increasing intervals (6 km and 8 km) until reaching the extreme value over which only 7% of amphibians have been found to disperse [i.e. 10 km; 42]. Hence, for each of the six species we ran models at 300 m, 1 km, 2 km, 4 km, 6 km, 8 km and 10 km, as well as at a previously reported species-specific maximum dispersal distances (Table 1).

**Table 1:** Dispersal distances reported for the species in our study and *d0* (Eq 1) values used to approximate them.

Species	Species-specific Max. Disp. Dist. (m)	Values of *d0*
*Alytes obstetricans*	1500 [53]	113
*Bombina variegata*	4000 [52]	301
*Epidalea calamita*	4411 [42]	332
*Hyla arborea*	2658 [41]	200
*Pelophylax lessonae* agg	1760 [54]	133
*Pelophylax ridibundus*	1760 [54]	133

### Boosted regression trees modelling and evaluation

For all the 47 generated networks (*Bombina variegata* did not need a separated 4 km maximum dispersal distance network, as 4 km was its reported maximum dispersal distance in the literature), we calculated the same topological variables as in Ortiz-Rodríguez et al. [41]. These were the degree [55], third-order neighborhood [21], unweighted betweenness centrality [23], strength [weighted version of degree; 56], and habitat availability [41], based on the probability of connectivity index [22]. We used all of them, as well as the HSI and the patch area as predictors of species occurrence-state in the boosted regression trees (BRT) models [57].

The response variable (occurrence-state) was defined by an approach based on sampling-intensity presented in Ortiz-Rodríguez et al. [41]. The number of times a patch was visited (sampling intensity) was determined by observations of any pond-based amphibian species. If a patch had been visited frequently to sample this group of species but the species of interest was not found, this species was assumed absent (also called target-group absences in [58]). With a plot of the average number of times a focal species was observed for every number of patch visits, we defined a sampling intensity threshold over which patches were considered unoccupied. This produced the binary occurrence-state values of presence (1) for the patches where the species was observed, and likely absence (0) for those where it was not found during visits to patches above the threshold.

For each network, we ran 100 BRT model iterations, with learning rate = 0.001, tree complexity = 5, and a bagging fraction of 0.75, following the general guidelines of Elith et al. [57], with the gbm.step function of the R‐package dismo [59]. For all the models, we observed, summarized and evaluated the area under the receiver operating characteristics curve (AUC). This is a measure of accuracy of a model, or more specifically, a discrimination metric, which shows numerically and graphically the sensitivity and specificity of a model [43, 60]. We used specifically the cross-validated AUC (henceforth AUC-cv), as cross-validation is a method to assess how generalizable is a model [61]. We also recorded the training AUC, the number of classification trees of the BRTs, the importance of each of the predictor variables, as well as partial dependence plots for relevant predictors and species. In these last plots, the fitted functions (scores on the response variables) were automatically centered by subtracting the mean [57]. Additionally, we measured the number of components in each of the networks.

## Results

### Network structure

The different species we considered for this study generated differing amounts of suitable habitat patches (Table 2). *Pelophylax lessonae* agg had the highest amount of habitat patches, and *P*. *ridibundus* the lowest.

**Table 2:** Number of edges and components for each setting of maximum dispersal distances tested and each species considered, with their corresponding number of patches and abbreviation.

Species	Abbreviation	Number of patches	Dispersal Distance (km)	Number of edges	Number of components
*Alytes obstetricans*	Alobs	2395	0.3	519	1907
			1.0	2441	1014
			1.5 (Species-specific)	4347	561
			2.0	6617	299
			4.0	19344	41
			6.0	37615	8
			8.0	61222	6
			10.0	89911	3
*Bombina variegata*	Bovar	2735	0.3	722	2050
			1	3129	980
			2	8435	257
			4 (Species-specific)	23959	34
			6	46006	7
			8	73582	3
			10	106671	1
*Hyla arborea*	Hyarb	1900	0.3	530	1398
			1	2169	706
			2	5644	235
			2.6 (Species-specific)	8398	134
			4	15022	41
			6	26886	9
			8	41412	4
			10	58286	2
*Epidalea calamita*	Epcal	2010	0.3	524	1516
			1	2155	831
			2	5745	308
			4	15814	53
			4.4 (Species-specific)	18427	35
			6	29425	15
			8	46564	6
			10	66357	1
*Pelophylax lessonae agg*	Peagg	3074	0.3	762	2351
			1	3509	1078
			1.7 (Species-specific)	7982	357
			2	9675	248
			4	29528	21
			6	56927	1
			8	91299	1
			10	132013	1
*Pelophylax ridibundus*	Perid	1256	0.3	393	882
			1	1563	413
			1.7 (Species-specific)	3231	193
			2	3782	155
			4	9453	39
			6	16511	18
			8	25195	7
			10	34627	5

**Fig 1:**
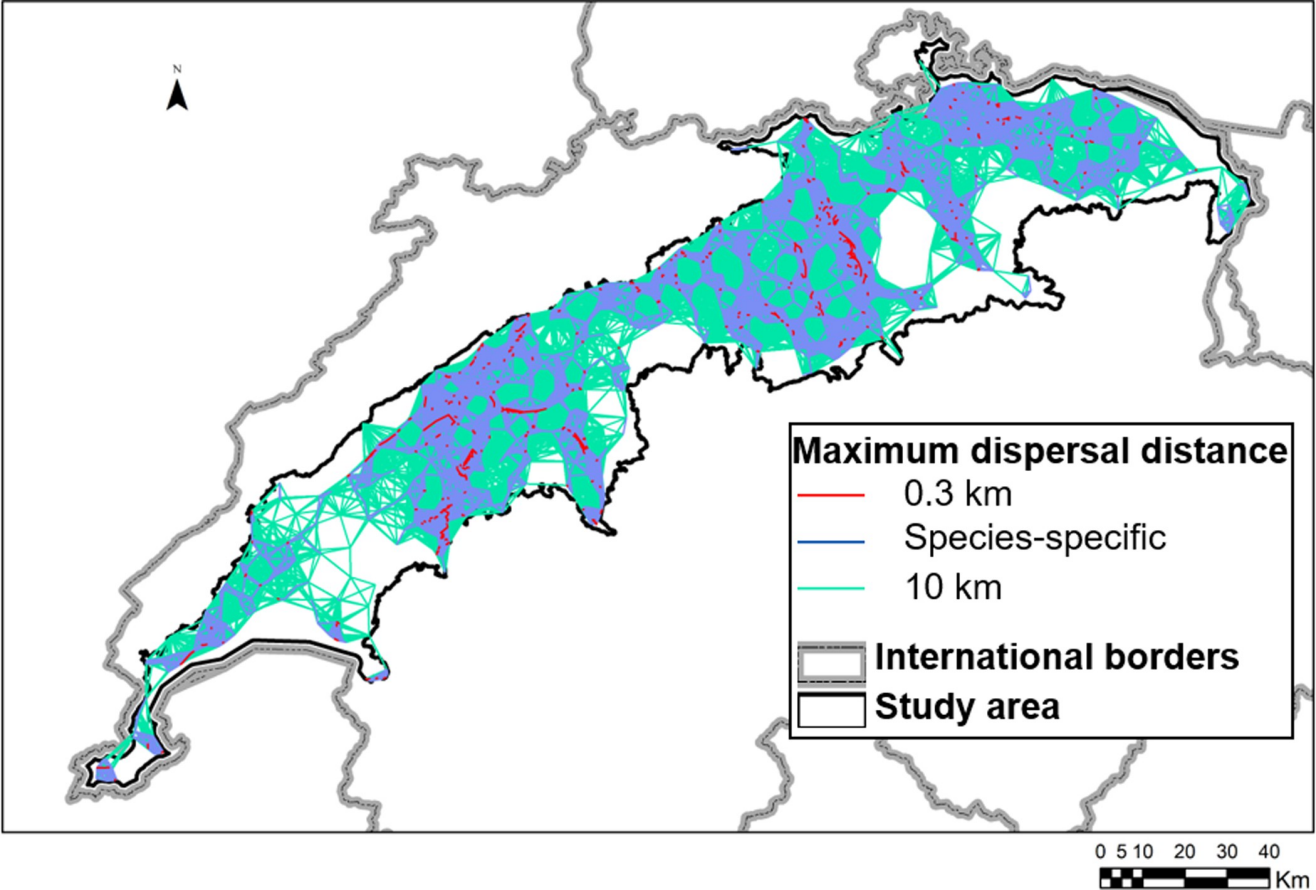
Example of overlaid habitat networks for the shortest (300 m), longest (10 km), and species-specific (4411 m) maximum dispersal distances for *Epidalea calamita*. Note that what might seem a solid surface is actually densely intertwined edges. Grey dotted line: international borders; black line: study area.

**Fig 2:**
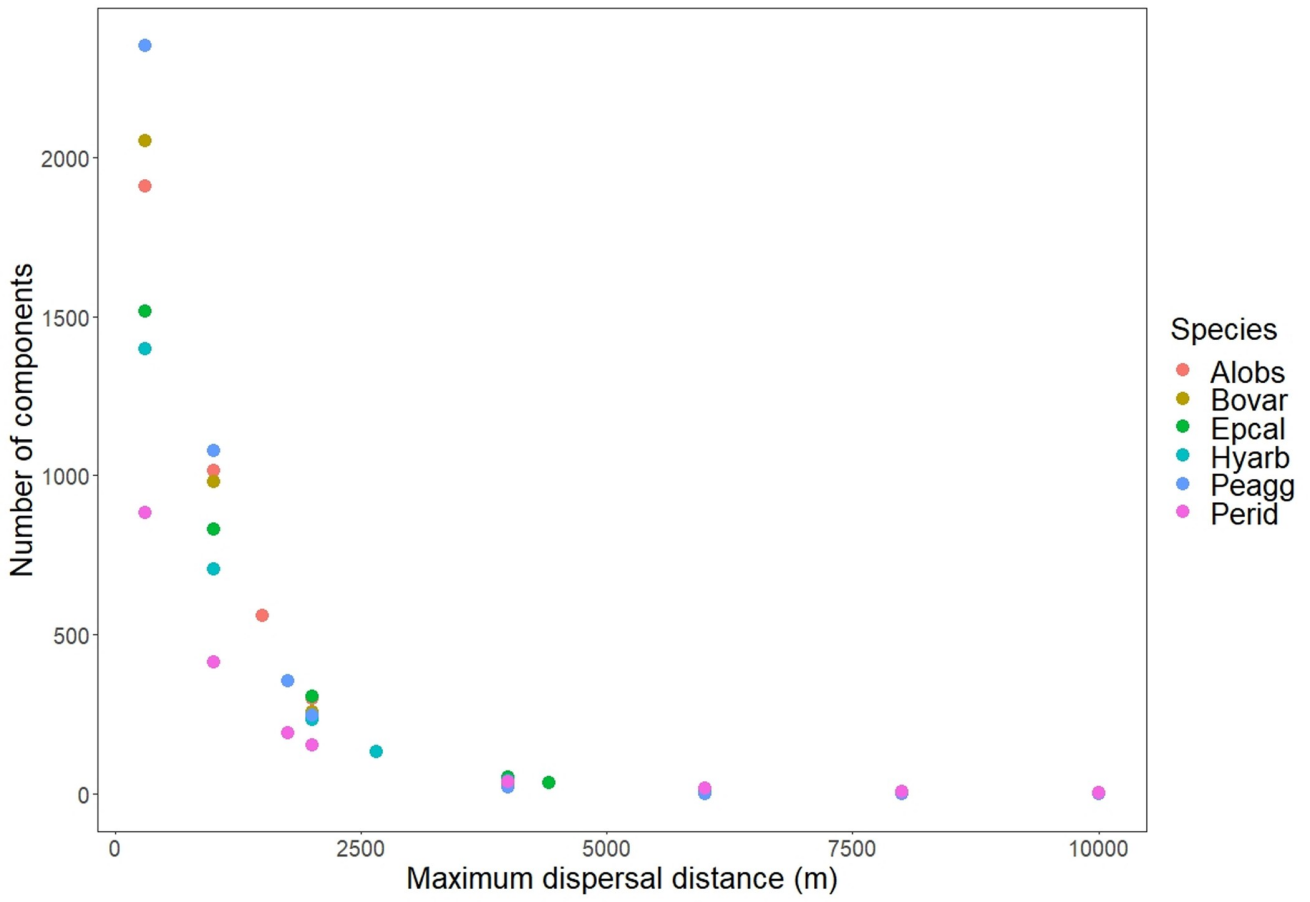
Number of components in relation to maximum dispersal distance for the networks of six amphibian species (colors). For abbreviations of species, see Table 2.

When the maximum dispersal distances were increased, the number of edges between patches also strongly increased (Table 2). Changing the dispersal distance also had an influence on the number of connected components: For all species, there was a plethora of small components for the shortest maximum dispersal distance (330 m), and a single giant component (plus a few additional small components for some species) for the longest one (10 km; Table 2; example in Fig 1). This resulted in an inverse relationship between the number of components and maximum dispersal distance (Fig 2). For the shortest maximum dispersal distance, there was a clear linear correlation between increasing number of components in the network and number of patches, which became less clear with increasing distance. At the longest maximum dispersal distances, they showed no relation whatsoever (Table 2 and S2 Appendix).

### Evaluation of predictive performance

**Fig 3:**
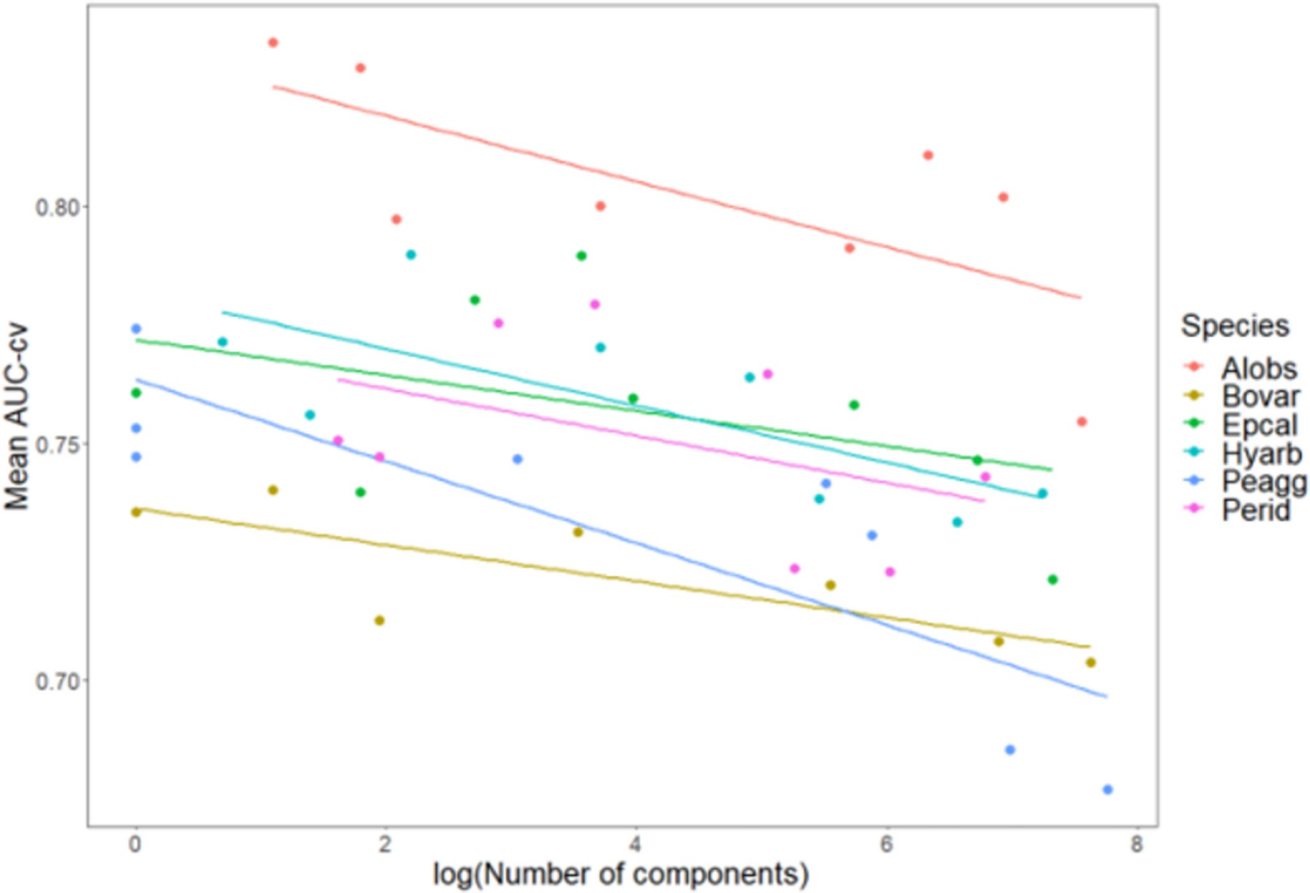
Scatterplot of the mean cross-validated AUC (AUC-cv) and the (log transformed) number of components of habitat networks for six amphibian species (colors), with linear trendlines. For abbreviation of species, see Table 2.

**Fig 4:**
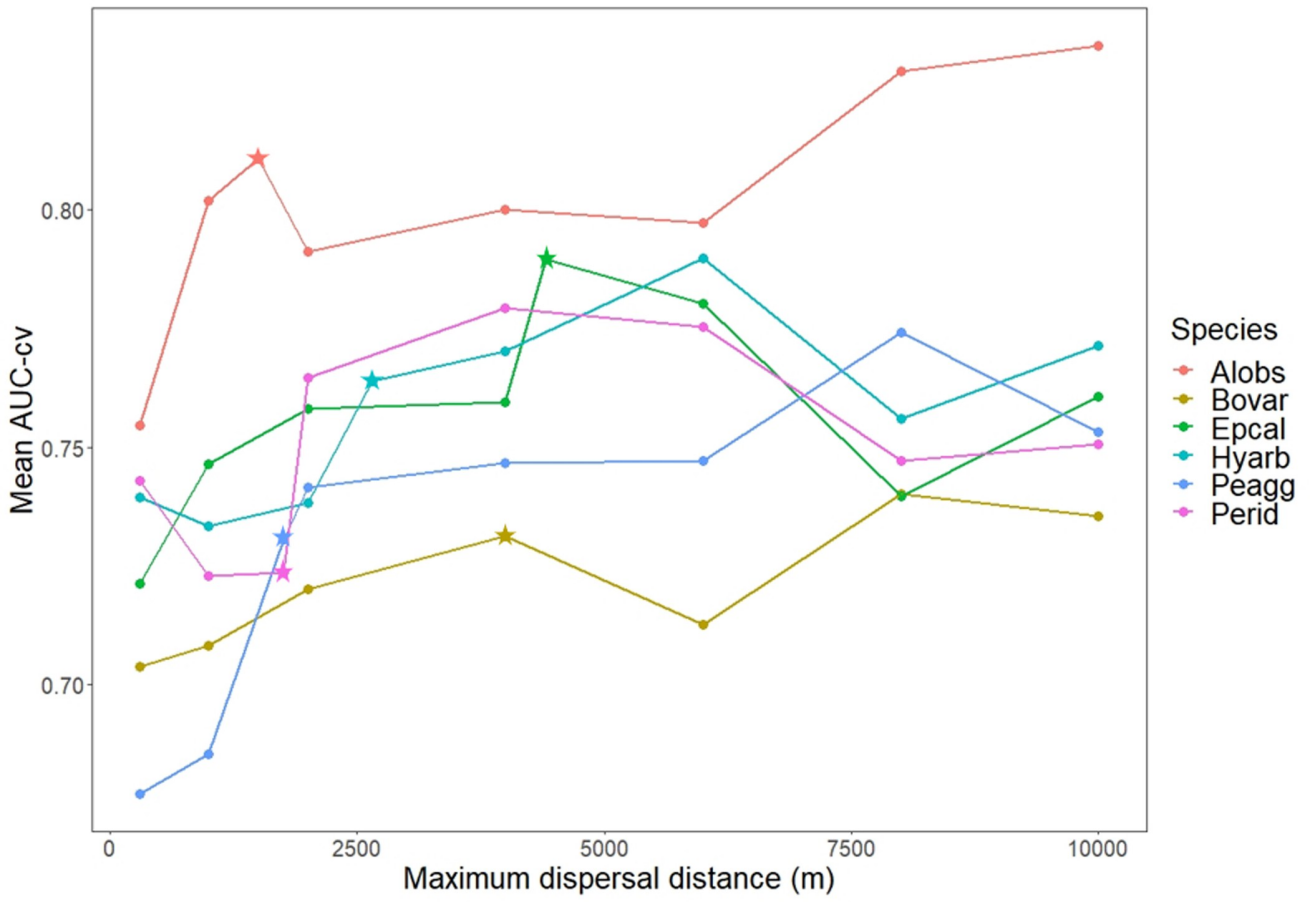
Scatterplot of the mean cross-validated AUC (AUC-cv) and the maximum dispersal distance for the six amphibian species tested. Stars indicate the species-specific maximum dispersal distances. For species abbreviations, see Table 2.

**Fig 5:**
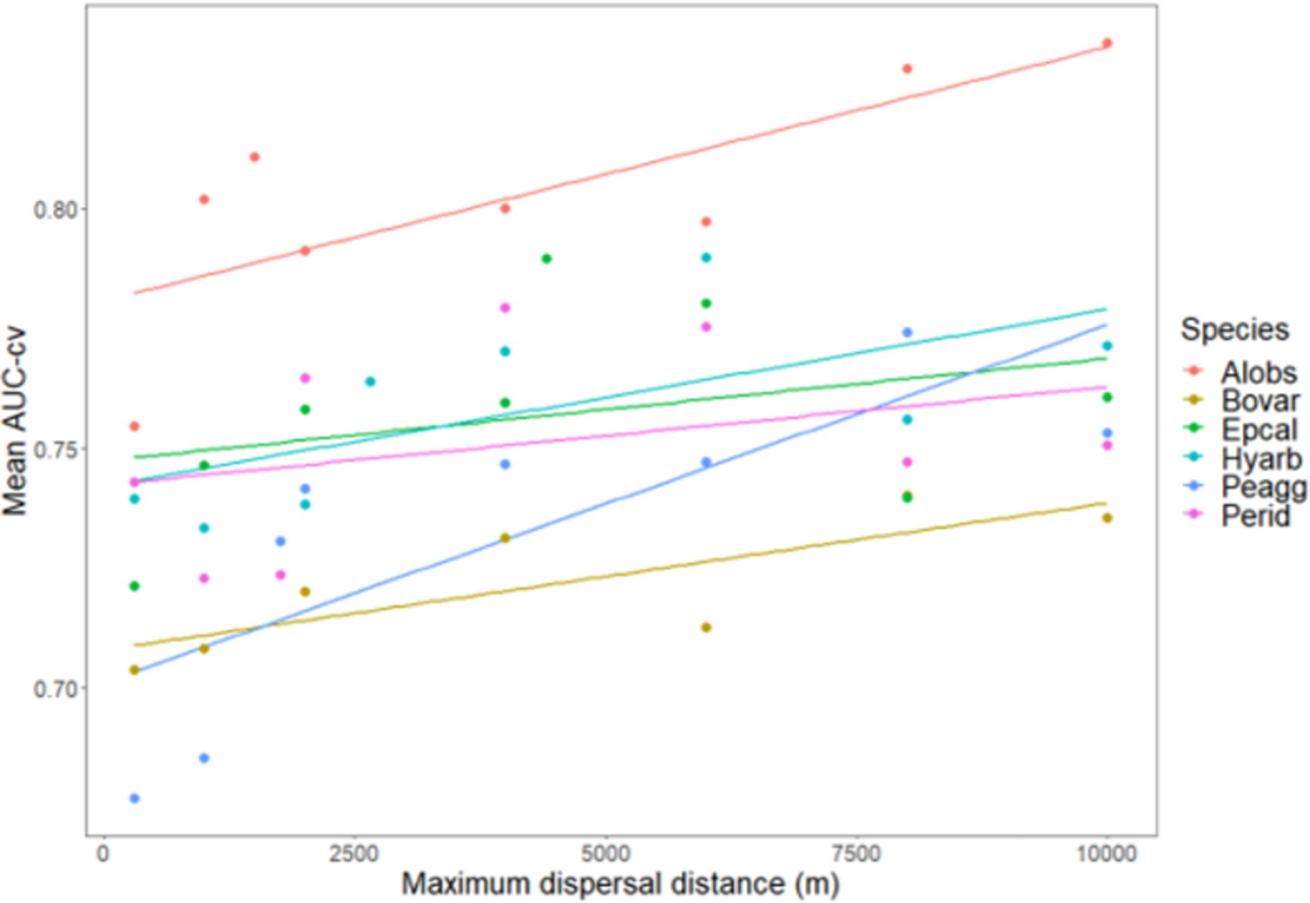
Scatterplot of mean cross-validated AUC (AUC-cv) and the maximum dispersal distance for the six amphibian species tested, with linear regression trendlines. For species abbreviations, see Table 2.

**Fig 6:**
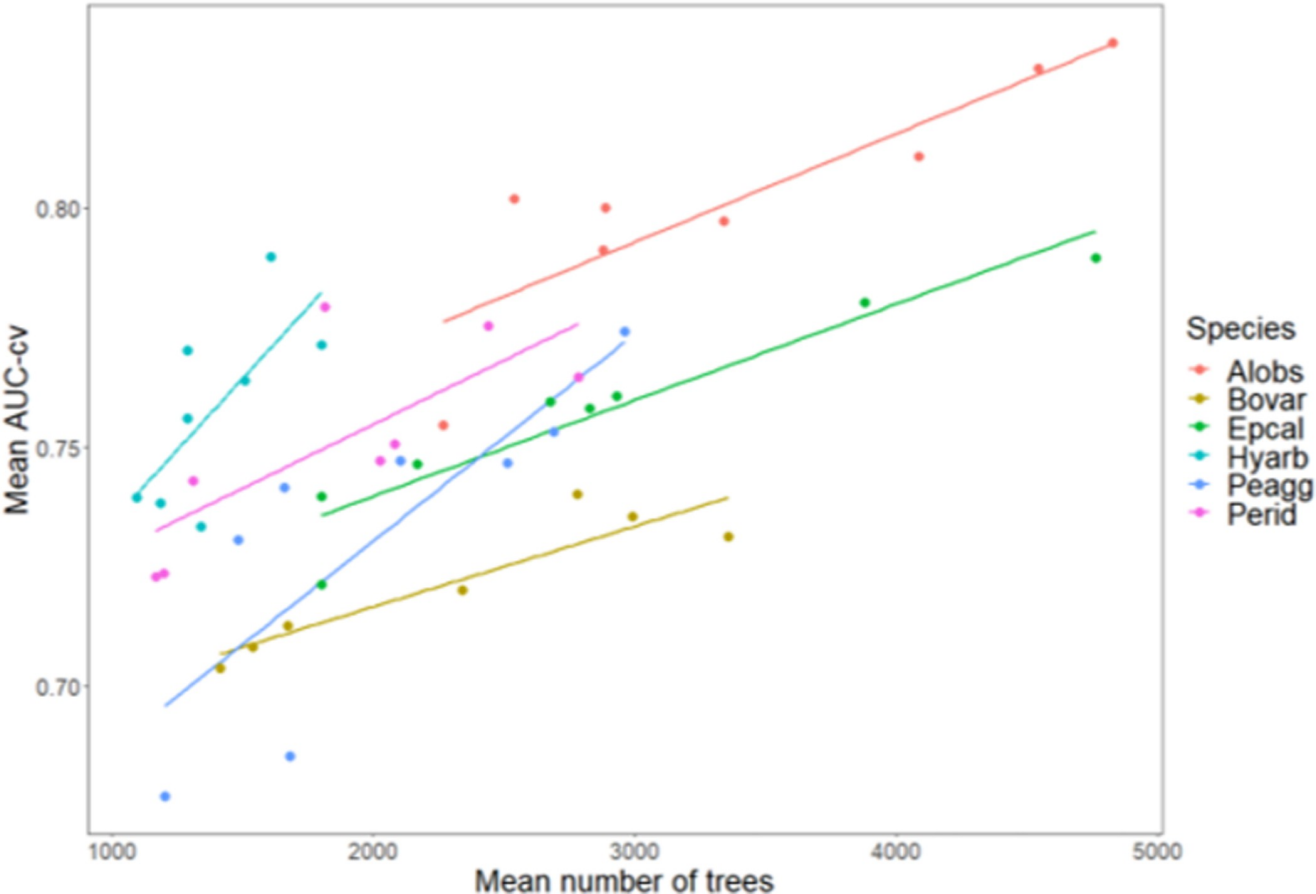
Scatterplot of mean cross-validated AUC (AUC-cv) and the mean number of regression trees used in the boosted regression trees (BRT) models, grouped by species, with trendlines. For species abbreviations, see Table 2.

Networks with less components had in general a higher AUC-cv (Fig 3 and S3 Appendix). There was no single maximum dispersal distance that had the best performance (in terms of mean AUC-cv) for all the six amphibian species (Figs 4 and 5). Assessing the relationship between maximum dispersal distance and AUC-cv generated by connecting the means, we observed non-monotonic, wave-like patterns in AUC-cv within the tested maximum dispersal distances for almost all species (shown for mean values of AUC-cv in Fig 4). These patterns were not regular when compared between the species. However, the shortest maximum dispersal distance (300 m) produced lower AUC-cv scores for all species than did the networks with the longest maximum dispersal distance (10 km), and AUC-cv was found to be positively correlated to increasing maximum dispersal distance in linear models for all the species (Table 3, shown for means in Fig 5). AUC-cv was also positively correlated to the mean number of regression trees found in the BRT models. For some species the highest number of trees, corresponding to high AUC-cv values, was much lower than for others (S4 Appendix and Fig 6).

**Table 3:** Significance (p-value), standard error, and R^2^ scores for the linear relation between maximum dispersal distance and cross-validated AUC (AUC-cv) across all the network models for each study species.

Species	P-value	R^2^	Std. Error
*Alytes obstetricans*	<2e-16 ***	0.5333	1.75E-07
*Bombina variegata*	<2e-16 ***	0.4368	1.32E-07
*Epidalea calamita*	<2e-16 ***	0.09503	2.33E-07
*Hyla arborea*	<2e-16 ***	0.2791	2.10E-07
*Pelophylax lessonae* agg	<2e-16 ***	0.5834	2.24E-07
*Pelophylax ridibundus*	3.36e-16 ***	0.0801	2.45E-07

lm (AUC-cv ∼ maximum dispersal distance)

In general, the mean AUC-cv of all models was above the 0.7 acceptability threshold ([62]; Fig 5). *Pelophylax lessonae* agg was the only species that produced a mean value below this threshold. It has to be noted, however, that considering the whole distribution of AUC-cv scores along the 100 model runs per maximum dispersal distance setting (per species), for some distances there were still many models below the threshold even though the mean was above it. The mean AUC-cv of the models produced with the species-specific reported maximum dispersal distances were all above the aforementioned AUC acceptability threshold. However, *E*. *calamita* was the only species for which its reported maximum dispersal distance yielded the highest mean AUC-cv of all the tested maximum dispersal distances. For *A*. *obstetricans* and *B*. *variegata* the species-specific maximum dispersal distances were at the local (not overall) maximum in AUC-cv, while for *H*. *arborea* and *P*. *lessonae* agg they were rather far from them (Fig 4). For *P*. *ridibundus*, the species-specific dispersal distance yielded one of its lowest AUC-cv scores across the tested maximum dispersal distances. The models with the highest mean AUC-cv at every tested distance were those performed on *A*. *obstetricans* (Fig 5).

### Predictor variable importance and partial dependence tendencies

**Fig 7:**
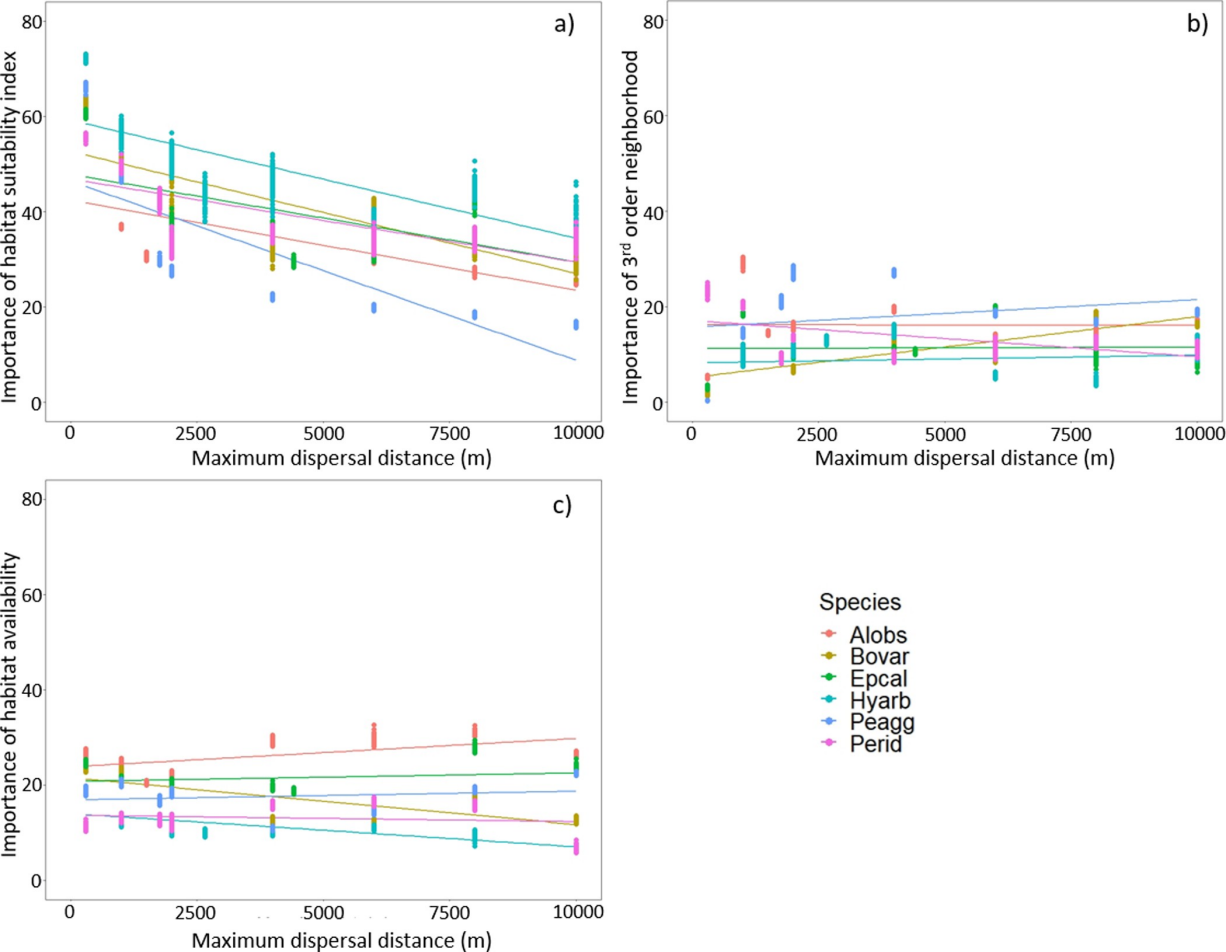
Relation of variable importance of three predictors to maximum dispersal distances in six amphibian species. (a) Habitat suitability index; (b) Third-order neighborhood; (c) Habitat availability. For species abbreviations see Table 2.

For several of the species, habitat suitability index (HSI) was the most important variable in the BRTs (Fig 7 and S5 Appendix). This was very clear at shorter maximum dispersal distances, but this was not always the case with larger dispersal distances. At larger maximum dispersal distances, habitat availability became more important for *A*. *obstetricans*, while this was true for four different topological predictors in the case of *Pelophylax lessonae* agg. Habitat availability and third-order neighborhood were among the most important predictors for several species and maximum dispersal distance settings (S5 Appendix). When overlaying the values of variable importance of all species for the three most consistently important predictor variables (i.e. HSI, habitat availability and third-order neighborhood), the topological variables did not present a discernible common pattern (Fig 7). Only for the shortest maximum dispersal distances, HSI had on average a much higher importance, with an overall decreasing importance with increasing dispersal distance (Fig 7).

**Fig 8:**
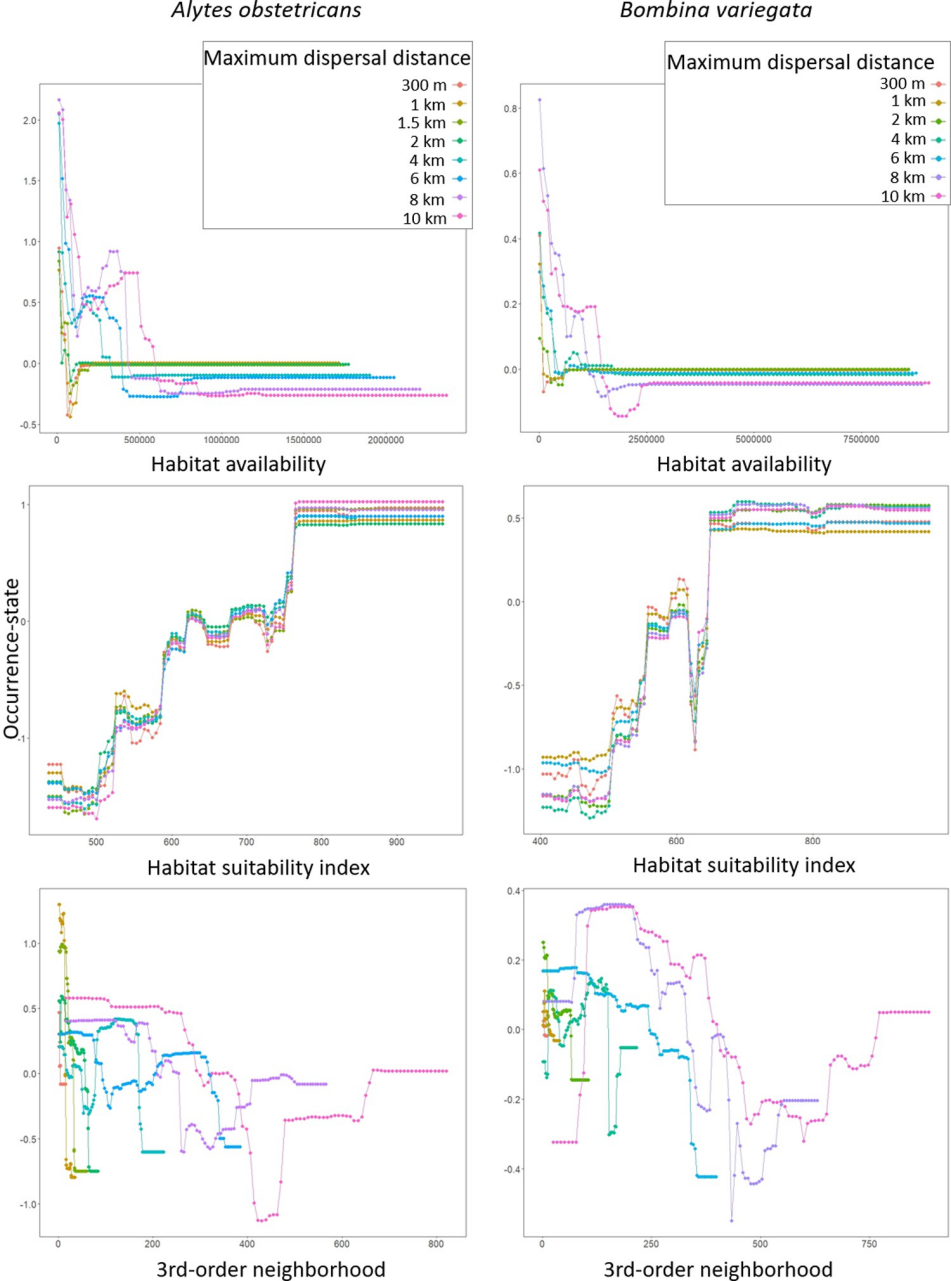
Partial dependence plots on the occurrence-state response of the habitat availability (top), habitat suitability index (center), and third-order neighborhood (bottom) predictor variables for all the maximum dispersal distance settings of *Alytes obstetricans* (left) and *Bombina variegata* (right). The fitted response scale is centered by subtraction of the mean.

The partial dependence plots of the species with both the highest (*A*. *obstetricans*) and the lowest (*B*. *variegata*) maximum AUC-cv values showed similarly marked differences between topological and quality-related variables in how consistent their response curves were among the different maximum dispersal distance settings. There was a strongly consistent pattern for HSI among their networks at all the tested maximum dispersal distances. This was a positive but noisy trend (Fig 8) for both species. The pattern was less consistent for habitat availability (habAv). Counterintuitively, low values of habitat availability were associated with presences and higher values were associated with absences (Fig 8). The response curves of the third-order neighborhood predictor showed even less of a consistent pattern (Fig 8). This was a difficult comparison because the complete range of values was not present for the lowest dispersal distances, as in these there were simply not many third order neighbors.

## Discussion

Our study shows prominently that previously reported maximum dispersal distances of amphibians are most probably underestimated, as has been suggested for animals in general [61]. Another main finding was that the importance of the predictor variables and the shape of their response curves change with increasing maximum dispersal distances. The results also notably indicated that the correlations between predictive performances and maximum dispersal distances fluctuated but were overall positive.

The tendency of overall higher AUC-cv with increasing maximum dispersal distances was consistent but showed considerable variation (Fig 5). Furthermore, for most of our study species the previously reported dispersal distances (Table 1) rarely corresponded to the highest AUC score, or best predictive performance. The only species which had the best performance at the reported maximum dispersal distance (*Epidalea calamita*) was also the one with the highest reported distance among our studied species. These findings point to the conclusion, supported by earlier insights [42, 63, 64], that the reported dispersal distances for most species are notoriously underestimated. Such underestimation is expected given that existing records are just snapshots of the process of dispersal, which as a population-wide process can have considerable variation over time. Presumably, the underestimation happens in large part because while most of the population disperse up to a limited distance, there are some rare long-distance dispersal events [65, 66]. This gives rise to a leptokurtic distribution of dispersal distances. Such dispersal distribution is ubiquitous in nature, as found in plants [26, 36], insects [67], mammals [68], birds [69], humans [70, 71], and of course amphibians [65, 72]. It is thus likely that the dispersal distances reported for our study species are also underestimated. Habitat network studies should consider this underestimation in the construction of the habitat networks and also experiment with longer distances than those reported in literature. In our study, choosing longer distances led to an increasing model fit in most cases.

A further implication of the underestimation of dispersal distances in literature, and therefore presumably also in the construction of habitat networks, is that many species are in reality less fragmented than the published dispersal distances make us believe. As a logical result of the positive correlation between model fit and maximum dispersal distance, we observed an inverse correlation of the predictive performance of our models with the number of components in the habitat network (Fig 3). If we assume that the models with the highest fit are closest to the true habitat networks of our focal species, the true habitat networks for most species are more connected than the one based on published maximum dispersal distances. Although well-connected habitat networks can bring disadvantages, such as an easier dispersal of infectious diseases [73], in general they provide benefits for species, such as alternative routes of dispersal in the face of harsh conditions and better chances of survival and reproduction [74, 75]. Given these benefits, the conservation status of our focal species in terms of habitat fragmentation may thus be more positive than what can be derived from literature. However, to substantiate this hypothesis, further research is needed to determine the validity of our assumption that the habitat network models with the highest fit best represent the true habitat networks.

Habitat quality-related predictors have proven to be highly important in models to predict species presence that also incorporate network topological variables [40, 41]. In our results, however, the importance of HSI compared to the other variables in the model decreased with increasing maximum dispersal distance, whereas the importance of topological predictors increased. This seems to follow the aforementioned relation between dispersal distance and topological integrity: network models with a short maximum dispersal distance produce a small topological neighbourhood, in which connectivity variables are less influential and the quality-related factors have a higher importance (Fig 7). Conversely, in scenarios where many patches are reachable (i.e., models in in which higher maximum dispersal distance is assumed), the influence of connectivity plays a bigger part in the likelihood of a species occurring in a habitat patch. We can derive from this that, in the exploration of models that approach the true topology of the habitat network, the assumed maximum dispersal distance will determine whether connectivity or sheer availability of quality habitat is interpreted as more important for species occurrence and hence richness [76, 77].

The sensitivity of the connectivity-related predictors to maximum dispersal distance in our models is also apparent from the response curves. As mentioned previously, the topological predictors showed very different response values for the same predictor values at the different dispersal distances (high response discordance, Fig 8), and showed quite similar values among the dispersal distances for the quality-related HSI. Meanwhile, for habitat availability, the variable that incorporates the influence of both connectivity and quality [22, 41], such discordance was intermediate. This again points towards a high sensitivity of the connectivity-related predictors to maximum dispersal distance, which follows from the completely different network topologies that can be generated in the models with different maximum dispersal distance assumptions.

While the variation in model performance applied to all species, for some of them the models were consistently better or worse than for other species. The species with the highest AUC-cv for every dispersal distance was *A*. *obstetrican*s, whereas the models *for B*. *variegata* were on average the worst performing for all dispersal distances above 1 km (Fig 4). Interestingly, unlike most pool-based amphibians, both of these species can been found in river courses [78], which entails the possibility of passive long-distance dispersal [66]. This could explain the high performance of *A*. *obstetricans*. While the overall better performance of models at higher dispersal distances also held for *B*. *variegata*, an alternative explanation remains to be found on the overall poorer performance for this species, whose dispersal distance and frequency has been found to vary between managed and largely untouched environments [72].

Another pattern worth noting was the correlation between AUC-cv and the mean number of regression trees used to build the BRT models (Fig 6). The algorithm we used for this purpose (gbm.step; [79]) searches for the number of regression trees that produces the lowest residual deviance. Since residual deviance is itself a measure of goodness of fit [80, 81], this will of course translate to a good score of our model predictive performance metric of choice (AUC-cv). However, for some species this optimum number of trees was much lower than for others. One possible influence on the number of trees could be the number of observations in the response variable, as adding more data points (presences and likely absences, in our case) should increase the chance that the decision trees yield a different response value for the same section of predictor space [57], which would potentially mean more trees are required to reach the lowest possible residual deviance. To substantiate this idea, further research should be done using different datasets to determine whether the number of trees and its correlation to model fit is indeed influenced by the sample size.

Besides their maximum dispersal distance, organisms have a diversity of dispersal methods, capabilities and behaviors, which results in a variety of dispersal kernels [10]. This variety leads to many possibilities of further research that are worth exploring at every step of the presented methodology. Perhaps the most obvious expansion for future research is using non-neutral cost-surfaces [41, 82–84], which could drastically change the topology of the network considering environmental barriers other than sheer distance. Other possibilities would be to use different network structure metrics to test our models, and to explore distributions of dispersal in the population by changing the proportion p of the population that reaches d0. Given the sensitivity of our models to the maximum dispersal distance parameter, occurrence-state could also be modelled with network topologies not defined by species dispersal distance such as the ubiquitous applications of circuit theory [7, 85], as used in the CircuitScape software [86, 87]. However, using circuit theory leaves out the perspective of the intrinsic locomotion capacities of the species. Furthermore, the application of circuit theory to predict species occurrence to our knowledge has been demonstrated on a case using intensive systematic sampling generating presence and absence data on tagged birds [88], which makes it so far unsuitable for less systematic databases and groups that are less easy to monitor. Another interesting possibility would be an approach that assesses the influence of connectivity factors on occurrence-state using continuous suitability surfaces instead of discrete habitat patches. The resistant-kernel estimator [64] is close to this. However, this method relies very much on expert opinion, and at larger scales ends up defining discrete limits that result on a tessellation of the continuous landscape. Therefore, using HSM and an environmental mask to define discrete patches still appears as the best option for analyses that aim to use habitat networks.

Overall, our findings indicate that maximum dispersal distance has a non-trivial and non-uniform, but overall positive relation to the fit of habitat network models predicting presence or absence of a species in habitat patches. The models are sensitive to the definition of the maximum dispersal distance parameter due to the vastly different network topologies that can emerge from it, and which define the size of the neighborhood of suitable patches. Therefore, for habitat network modelling any reported maximum dispersal distance has to be treated carefully and it should be acknowledged that it is likely that a non-negligible amount of individuals are dispersing at much further distances than estimated from field observations.

## Supporting information

S1 Appendix Settings for Habitat Suitability Modelling (HSM). (DOCX)

S2 Appendix Relation between number of components and number of patches of the networks of six amphibian species with different maximum dispersal distance settings (colors). The distances with an asterisk (*) are exclusively species-specific.
(DOCX)

S3 Appendix Significance (p-value), standard error, and R^2^ scores for the linear relation between number of components and cross-validated AUC (AUC-cv) for across all the network models for each study species. (DOCX)

S4 Appendix Mean cross-validated AUC (AUC-cv) and number of regression trees used in the boosted regression trees (BRT) models for the networks of all the species at every dispersal distance setting. (DOCX)

S5 Appendix BRT variable importance plots for all models (all species and maximum dispersal distance settings). In each figure: 3^rd^. org. neigh = third-order neighborhood, B.C. = betweenness centrality, Hab. Av. = habitat availability, HSI = habitat suitability index.
(DOCX)
