# Supplementary material for: Sensitivity of habitat network models to changes in maximum dispersal distance
Source: PLoS One. 2023 Nov 6;18(11):e0293966. doi: 10.1371/journal.pone.0293966 (PMC10627463; doi:10.1371/journal.pone.0293966)
Supplement: S1 Appendix — (DOCX) [file pone.0293966.s001.docx]

**S1 Appendix.** Settings for Habitat Suitability Modelling (HSM)

For each of the six species, we performed ensemble habitat suitability modelling with the R-package Biomod2 [1] with 20 predictor variables (Table S1A) We used the mean ensemble of three runs each of generalized linear (GLM), random forest (RF), and maximum entropy (MaxEnt) models. This was done with default settings, 10,000 pseudoabsences and one round of pseudoabsence selection. As in Ortiz-Rodríguez et al. [2:10460], all our models were evaluated “with ROC AUC, with a quality threshold of AUC ≥ 0.7” [3]. We also used the same criterion to binarize the continuous habitat suitability maps, which was “the point in the ROC curve that minimizes the difference between sensitivity and specificity.”

**Table S1A.** Predictor variables of HSM for all the species.

| Category | Predictor | Variable type | Source |
| --- | --- | --- | --- |
| Human influence | Density of traffic | Continuous | NPVM with tunnels removed [4] |
|  | Density of railways | Continuous | SwissTLM3D [5] |
|  | Total noise at night-time | Continuous | EMPA [6] |
|  | Population density | Continuous | STATPOP [7] |
|  | Agriculture density | Continuous | Arealstatistik [8] |
|  | Arable land | Binary | Arealstatistik [8] |
|  | Green settlements | Binary | Arealstatistik [8] |
|  | Grey settlements | Binary | Arealstatistik [8] |
|  | Meadows and farm pastures | Binary | Arealstatistik [8] |
|  | Orchards, vineyards, horticulture | Binary | Arealstatistik [8] |
| Natural landscape features | Deciduous forest coverage | Binary | Waldmischungsgrad [9] |
|  | Mixed forest coverage | Binary | Waldmischungsgrad [9] |
|  | Coniferous forest coverage | Binary | Waldmischungsgrad [9] |
|  | Density of forest | Continuous | Waldmischungsgrad [9] |
|  | Distance to forest edge | Continuous | Waldmischungsgrad [9] |
|  | Presence of rivers | Binary | SwissTLM3D [5] |
|  | Slope | Continuous | swissALTI3D [10] |
| Climatic variables | Mean summer precipitation | Continuous | Broennimann  *et al.* [11] |
|  | Mean annual direct solar radiation | Continuous | Broennimann *et al.* [11] |
|  | Mean annual temperature | Continuous | Broennimann *et al.* [11] |

**References**

1. Thuiller W, Georges D, Engler R, Breiner F. Package ‘biomod2’. 2016. Available from: https://cran.r-project.org/package=biomod2

2. Ortiz-Rodríguez DO, Guisan A, Holderegger R, van Strien MJ. Predicting species occurrences with habitat network models. Ecology and Evolution. 2019;9(18):10457-71. doi: https://doi.org/10.1002/ece3.5567.

3. Bulluck L, Fleishman E, Betrus C, Blair R. Spatial and temporal variations in species occurrence rate affect the accuracy of occurrence models. Global Ecology and Biogeography. 2006;15(1):27-38. doi: doi:10.1111/j.1466-822X.2006.00170.x.

4. Bundesamt für Raumentwicklung ARE. Nationales Personenverkehrsmodell des UVEK (NPVM). ARE. 2010. Available from https://www.are.admin.ch/are/de/home/verkehr-und-infrastruktur/grundlagen-und-daten/verkehrsmodellierung/nationales-personenverkehrsmodell.html

5. Office fédéral de topographie Swisstopo. Catalogue des objets swissTLM3D 1.4. Swisstopo. 2016. Available from: https://www.swisstopo.admin.ch/de/geodata/landscape/tlm3d.html

6. EMPA. Aufbereitung von flächendeckenden Grundlagen für die Schallausbreitungsmodellierung in den Bereichen Meteorologie und Bodeneigenschaften. EMPA. 2011. Available from: https://www.bafu.admin.ch/bafu/de/home/themen/laerm/publikationen-studien/publikationen/strassenlaerm-berechnungsmodell_sonroad18.html

7. BFS Bundesamt für Statistik. Statistik der Bevölkerung und der Haushalte (STATPOP). BFS. 2015. Available from: https://www.bfs.admin.ch/bfs/en/home/statistics/population/surveys/statpop.html

8. OFS Office fédéral de la statistique. Statistique de la superficie selon nomenclature 2004 – Occupation du sol (Land Cover). OFS. Berne 2010. Available from: https://www.bfs.admin.ch/bfs/fr/home/statistiques/espace-environnement/enquetes/area.html

9. BFS Bundesamt für Statistik. Waldmischungsgrad der Schweiz. BFS. 2013. Available from:https://www.bfs.admin.ch/bfs/de/home/dienstleistungen/geostat/geodaten-bundesstatistik/boden-nutzung-bedeckung-eignung/abgeleitete-und-andere-daten/waldmischungsgrad-schweiz.html

10. Swisstopo Office fédéral de topographie. swissALTI3D: The high precision digital elevation model of Switzerland. Swisstopo. 2018. Available from: https://www.swisstopo.admin.ch/en/geodata/height/alti3d.html

11. Broennimann O, Randin C, Zimmermann NE, Guisan A. Swiss Eco-Climatic GIS data. Ecospat - Spatial Ecology Group Université de Lausanne & WSL; 2003. Available from: https://www.unil.ch/ecospat/home/menuguid/ecospat-resources/data.html#chclim25
