# Supplementary material for: Sensitivity of habitat network models to changes in maximum dispersal distance
Source: PLoS One. 2023 Nov 6;18(11):e0293966. doi: 10.1371/journal.pone.0293966 (PMC10627463; doi:10.1371/journal.pone.0293966)
Supplement: S2 Appendix — The distances with an asterisk (*) are exclusively species-specific. (DOCX) [file pone.0293966.s002.docx]

**S2 Appendix.** Relation between number of components and number of patches of the networks of six amphibian species with different maximum dispersal distance settings (colors). The distances with an asterisk (*) are exclusively species-specific.

**
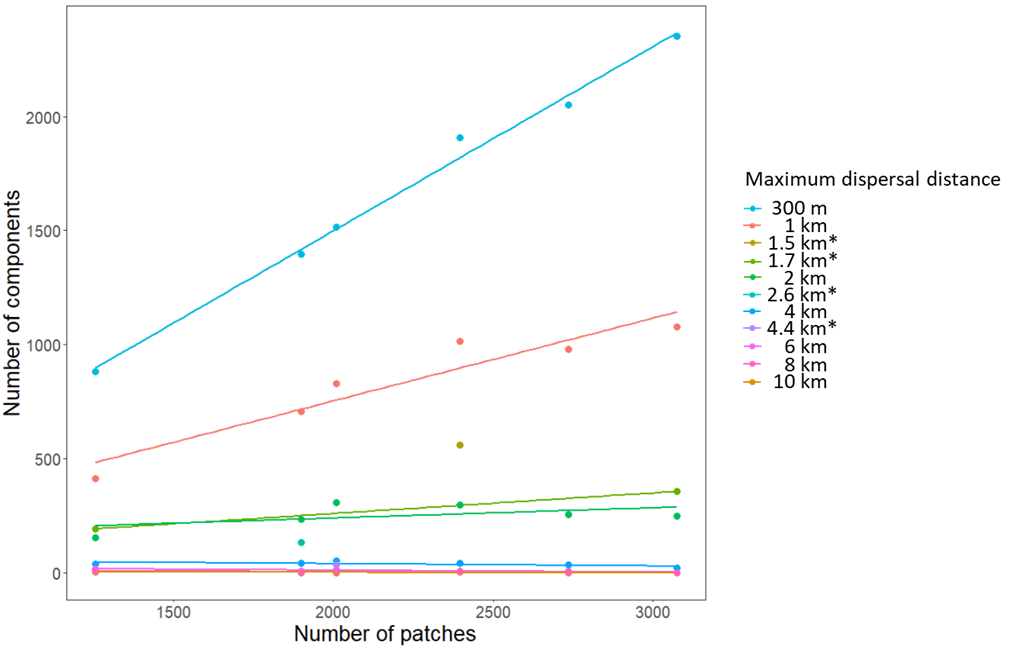
**
