# Supplementary material for: Sensitivity of habitat network models to changes in maximum dispersal distance
Source: PLoS One. 2023 Nov 6;18(11):e0293966. doi: 10.1371/journal.pone.0293966 (PMC10627463; doi:10.1371/journal.pone.0293966)
Supplement: S3 Appendix — (DOCX) [file pone.0293966.s003.docx]

**S3 Appendix.** Significance (p-value), standard error, and R^2^ scores for the linear relation between number of components and cross-validated AUC for across all the network models for each study species.

**lm (AUC_cv ~ number of components)**

| **Species** | **P-value** | **R^2^** | **Std. Error** |
| --- | --- | --- | --- |
| *Alytes obstetricans* | <2e-16 *** | 9.11E-07 | 9.11E-07 |
| *Bombina variegata* | <2e-16 *** | 6.46E-07 | 6.46E-07 |
| *Epidalea calamita* | <2e-16 *** | 1.13E-06 | 1.13E-06 |
| *Hyla arborea* | <2e-16 *** | 1.44E-06 | 1.44E-06 |
| *Pelophylax* *lessonae* agg | <2e-16 *** | 6.96E-07 | 6.96E-07 |
| *Pelophylax ridibundus* | <2e-16 *** | 2.77E-06 | 2.77E-06 |
