# Supplementary material for: Sensitivity of habitat network models to changes in maximum dispersal distance
Source: PLoS One. 2023 Nov 6;18(11):e0293966. doi: 10.1371/journal.pone.0293966 (PMC10627463; doi:10.1371/journal.pone.0293966)
Supplement: S4 Appendix — (DOCX) [file pone.0293966.s004.docx]

**S4 Appendix.** Mean cross-validated AUC (AUC-cv) and number of regression trees used in the boosted regression trees (BRT) models for the networks of all the species at every dispersal distance setting.

| **Species** | **Dispersal Distances  (km)** | **Mean AUC-cv** | **Mean number of trees** |
| --- | --- | --- | --- |
| *Alytes obstetricans* | 0.3 | 0.7545 | 2268 |
|  | 1 | 0.8019 | 2540 |
|  | 1.5 (Species-specific) | 0.8107 | 4083 |
|  | 2 | 0.7912 | 2878 |
|  | 4 | 0.8 | 2890 |
|  | 6 | 0.7972 | 3340 |
|  | 8 | 0.8293 | 4540 |
|  | 10 | 0.8347 | 4828 |
| *Bombina variegata* | 0.3 | 0.7038 | 1414 |
|  | 1 | 0.7083 | 1540 |
|  | 2 | 0.7201 | 2342 |
|  | 4 (Species-specific) | 0.7313 | 3356 |
|  | 6 | 0.7126 | 1675 |
|  | 8 | 0.7401 | 2778 |
|  | 10 | 0.7356 | 2990 |
| *Hyla arborea* | 0.3 | 0.7394 | 1096 |
|  | 1 | 0.7335 | 1340 |
|  | 2 | 0.7383 | 1187 |
|  | 2.6 (Species-specific) | 0.764 | 1511 |
|  | 4 | 0.7703 | 1292 |
|  | 6 | 0.7898 | 1610 |
|  | 8 | 0.7559 | 1290 |
|  | 10 | 0.7714 | 1804 |
| *Epidalea calamita* | 0.3 | 0.7214 | 1803 |
|  | 1 | 0.7465 | 2168 |
|  | 2 | 0.7581 | 2826 |
|  | 4 | 0.7595 | 2675 |
|  | 4.4 (Species-specific) | 0.7897 | 4761 |
|  | 6 | 0.7803 | 3879 |
|  | 8 | 0.7396 | 1804 |
|  | 10 | 0.7606 | 2932 |
| *Pelophylax* agg. | 0.3 | 0.677 | 1204 |
|  | 1 | 0.6854 | 1680 |
|  | 1.7 (Species-specific) | 0.7306 | 1486 |
|  | 2 | 0.7415 | 1658 |
|  | 4 | 0.7466 | 2515 |
|  | 6 | 0.7471 | 2102 |
|  | 8 | 0.7742 | 2962 |
|  | 10 | 0.7533 | 2690 |
| *Pelophylax ridibundus* | 0.3 | 0.743 | 1312 |
|  | 1 | 0.723 | 1172 |
|  | 1.7 (Species-specific) | 0.7237 | 1202 |
|  | 2 | 0.7646 | 2786 |
|  | 4 | 0.7793 | 1815 |
|  | 6 | 0.7755 | 2438 |
|  | 8 | 0.7472 | 2026 |
|  | 10 | 0.7506 | 2082 |
