# Supplementary material for: Sensitivity of habitat network models to changes in maximum dispersal distance
Source: PLoS One. 2023 Nov 6;18(11):e0293966. doi: 10.1371/journal.pone.0293966 (PMC10627463; doi:10.1371/journal.pone.0293966)

**S5 Appendix.** BRT variable importance plots for all models (all species and maximum dispersal distance settings). In each figure: 3^rd^. org. neigh = third-order neighborhood, B.C. = betweenness centrality, Hab. Av. = habitat availability, HSI = habitat suitability index.

***Alytes obstetricans***
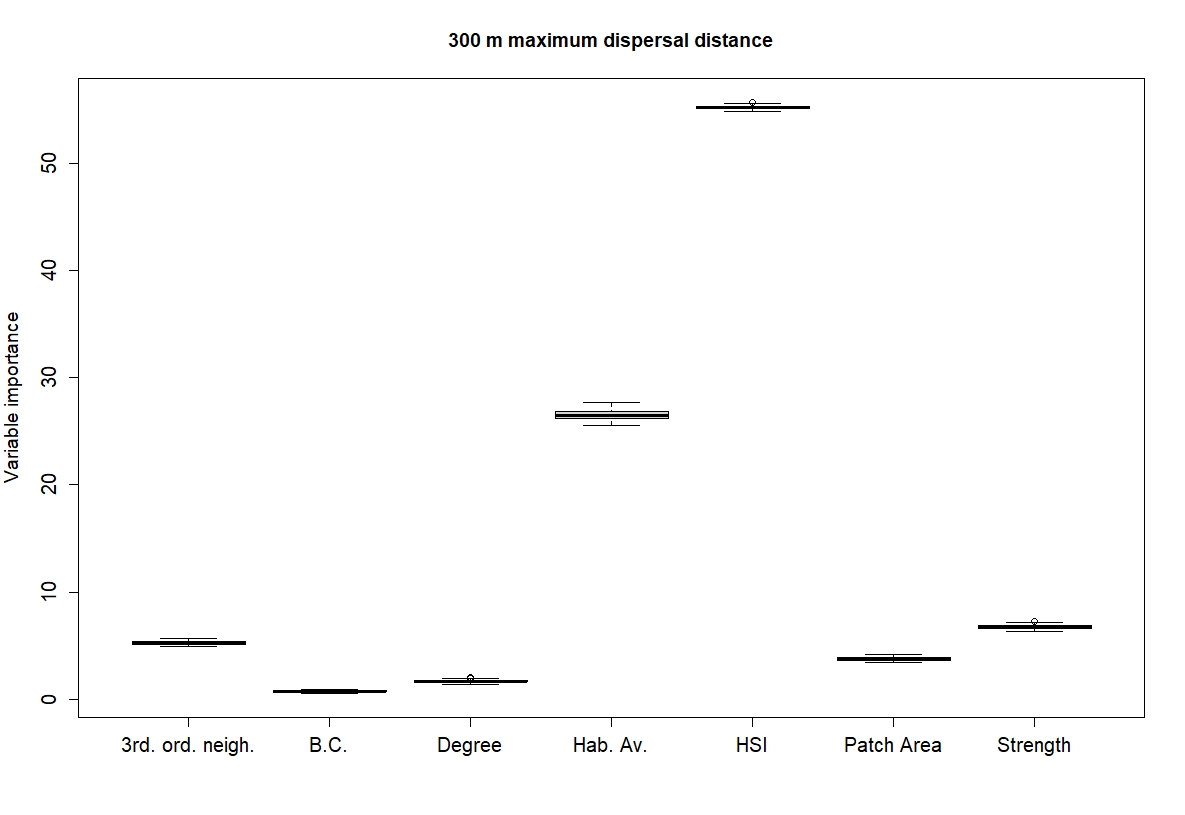


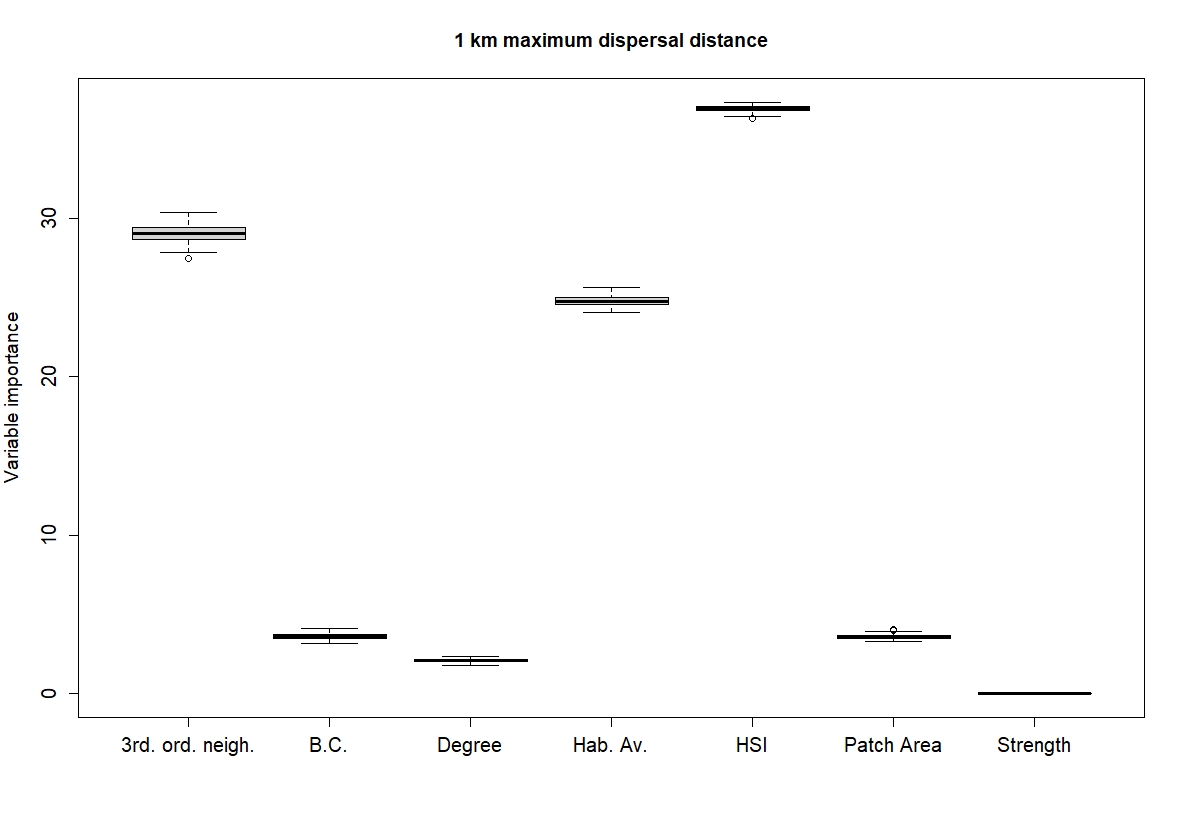


***Alytes obstetricans***


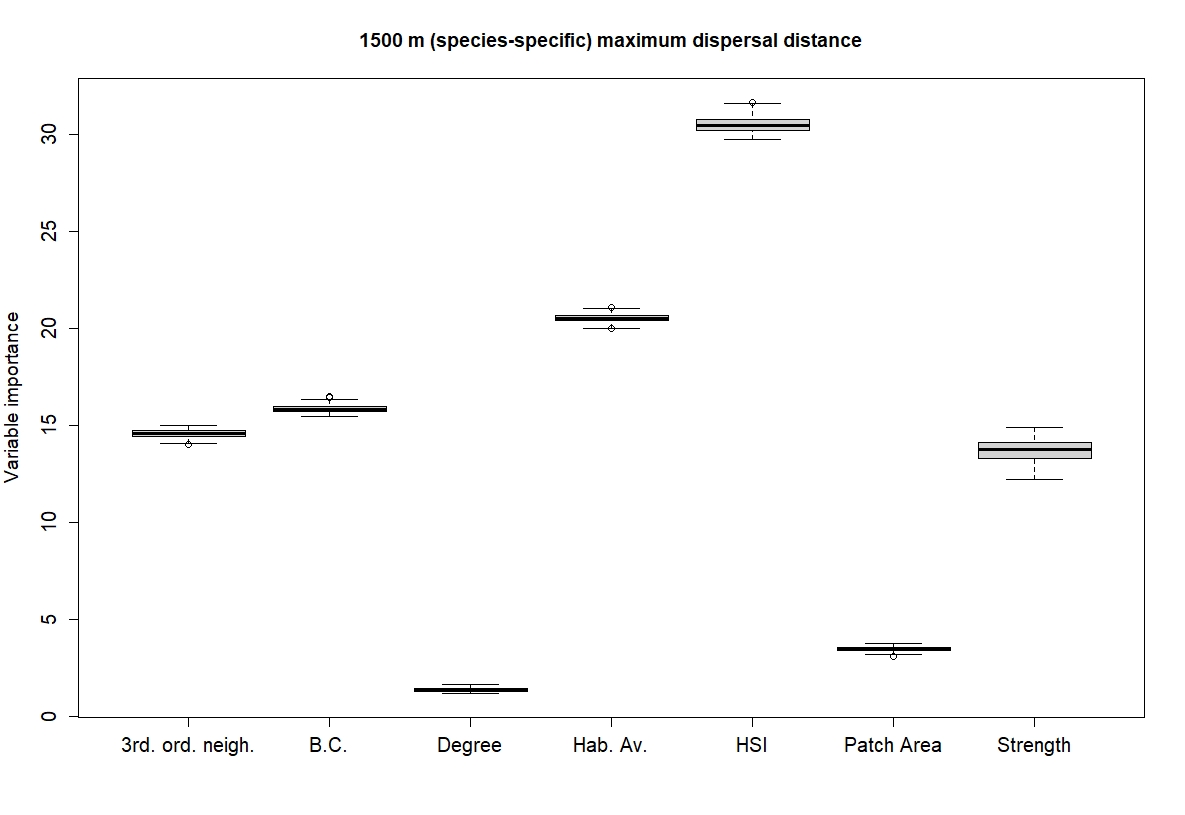


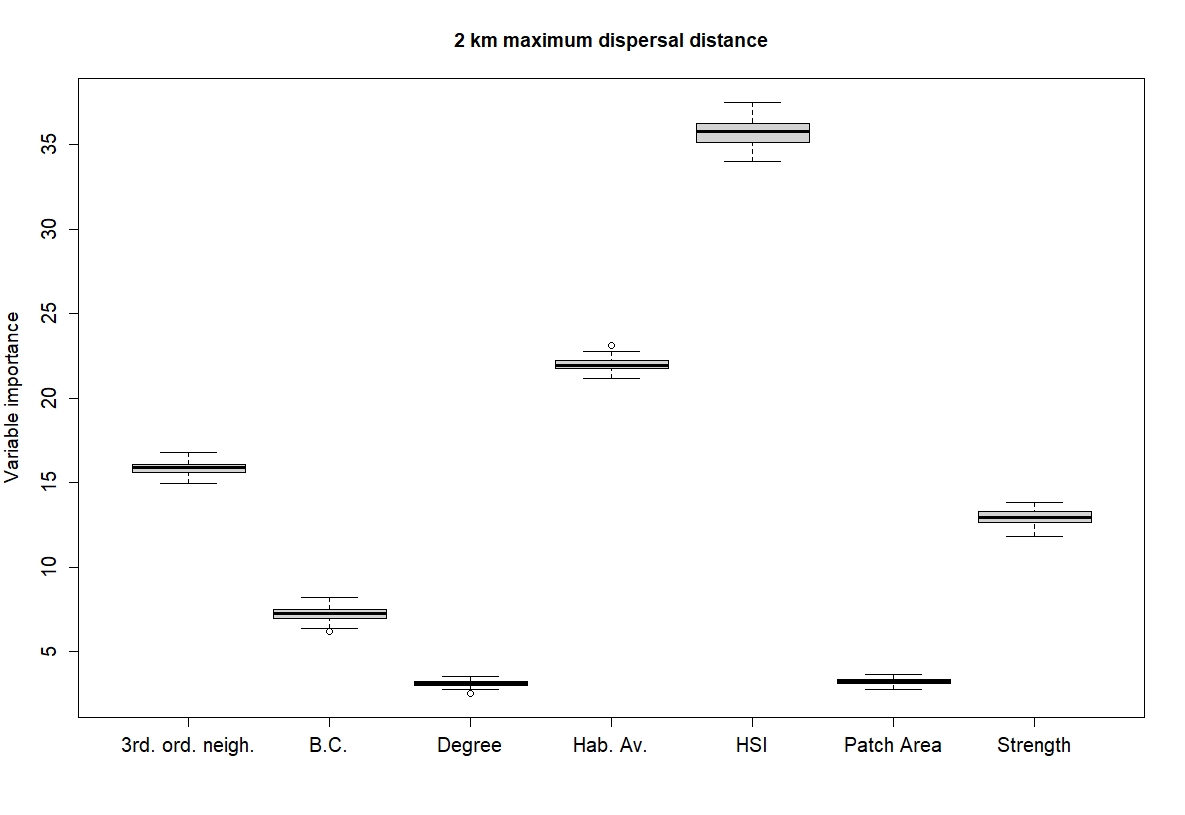


***Alytes obstetricans***


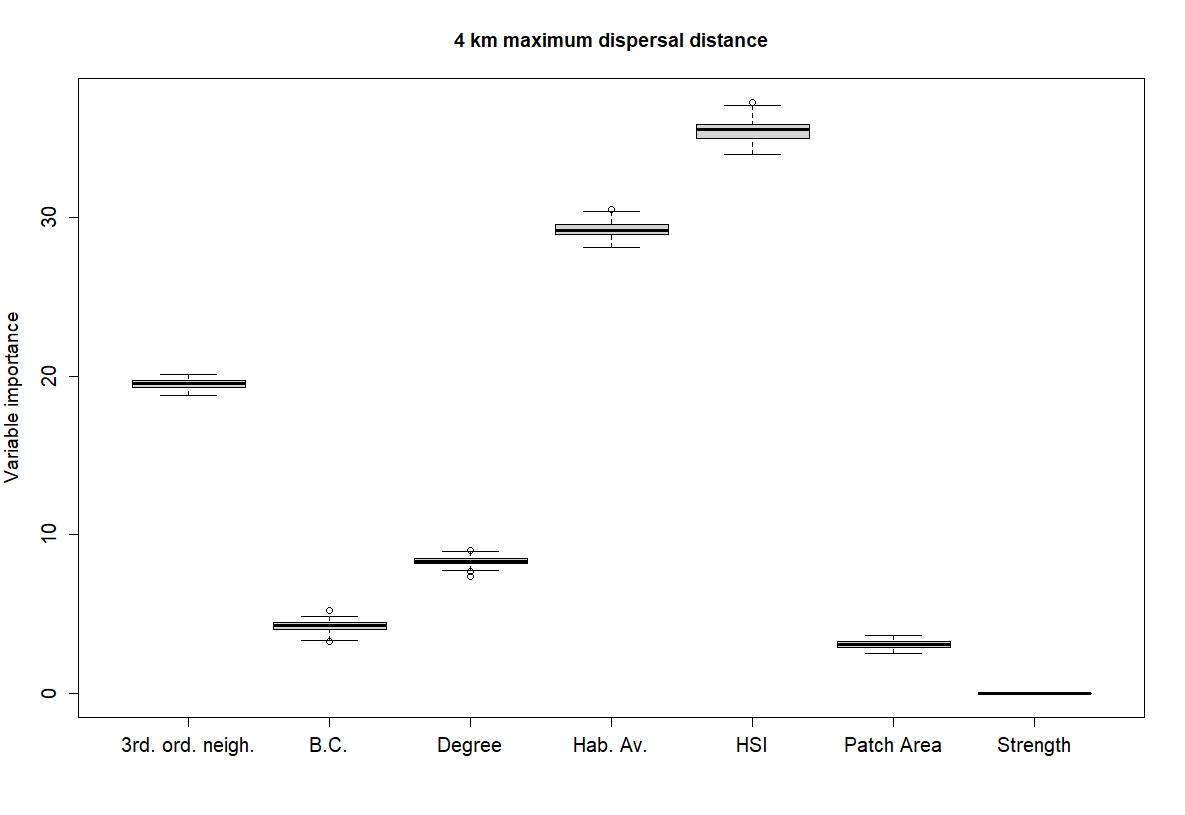

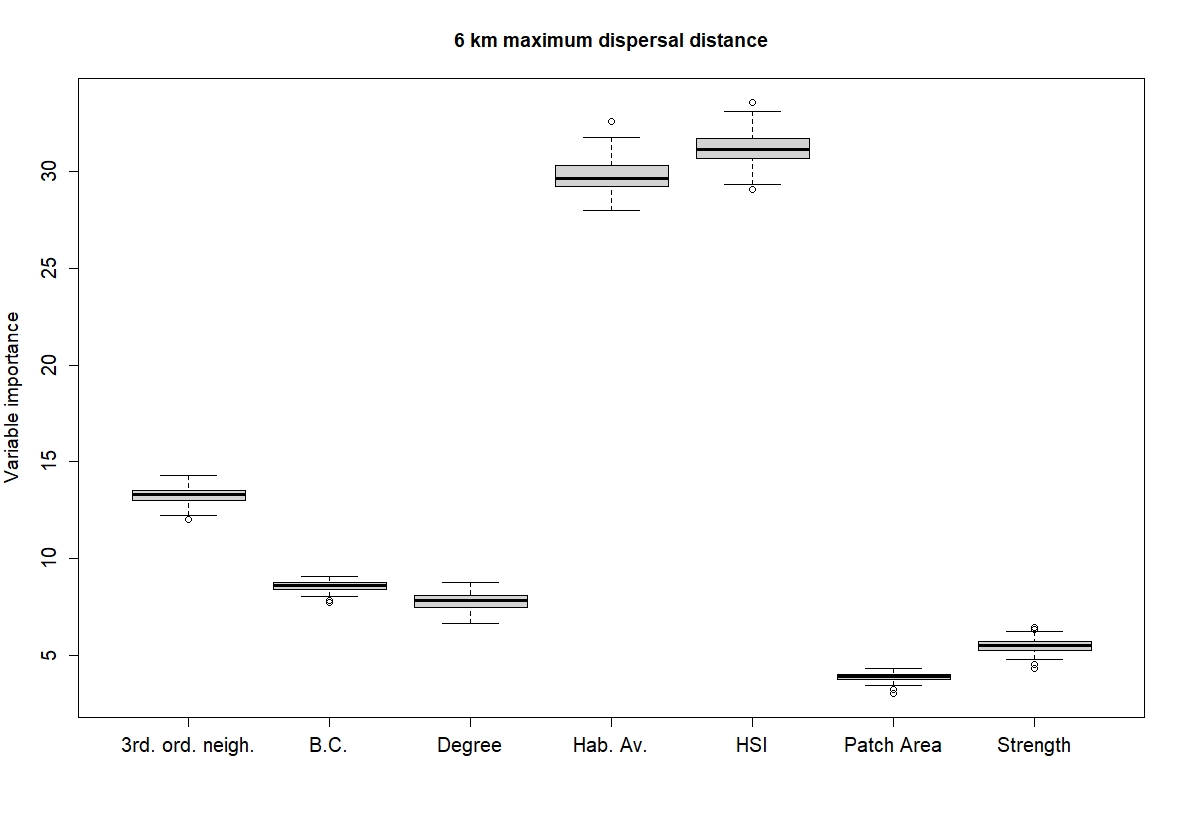


***Alytes obstetricans***


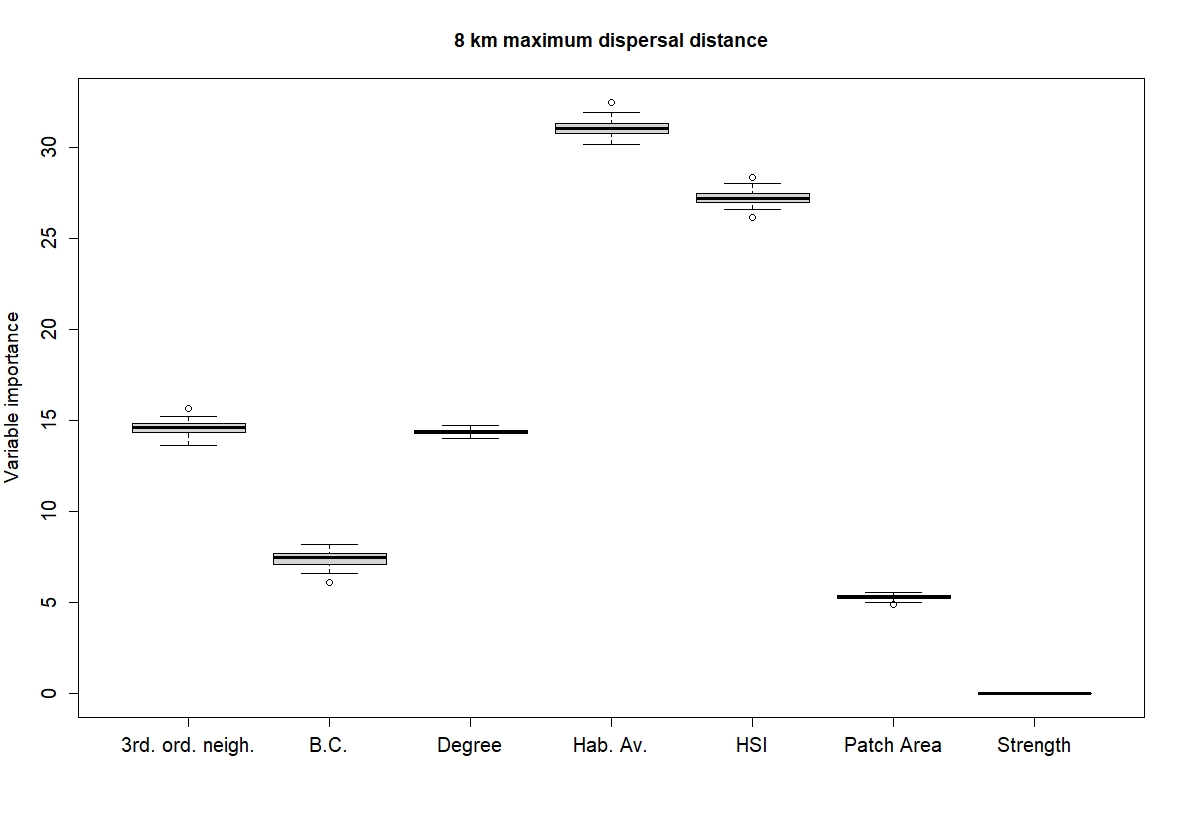

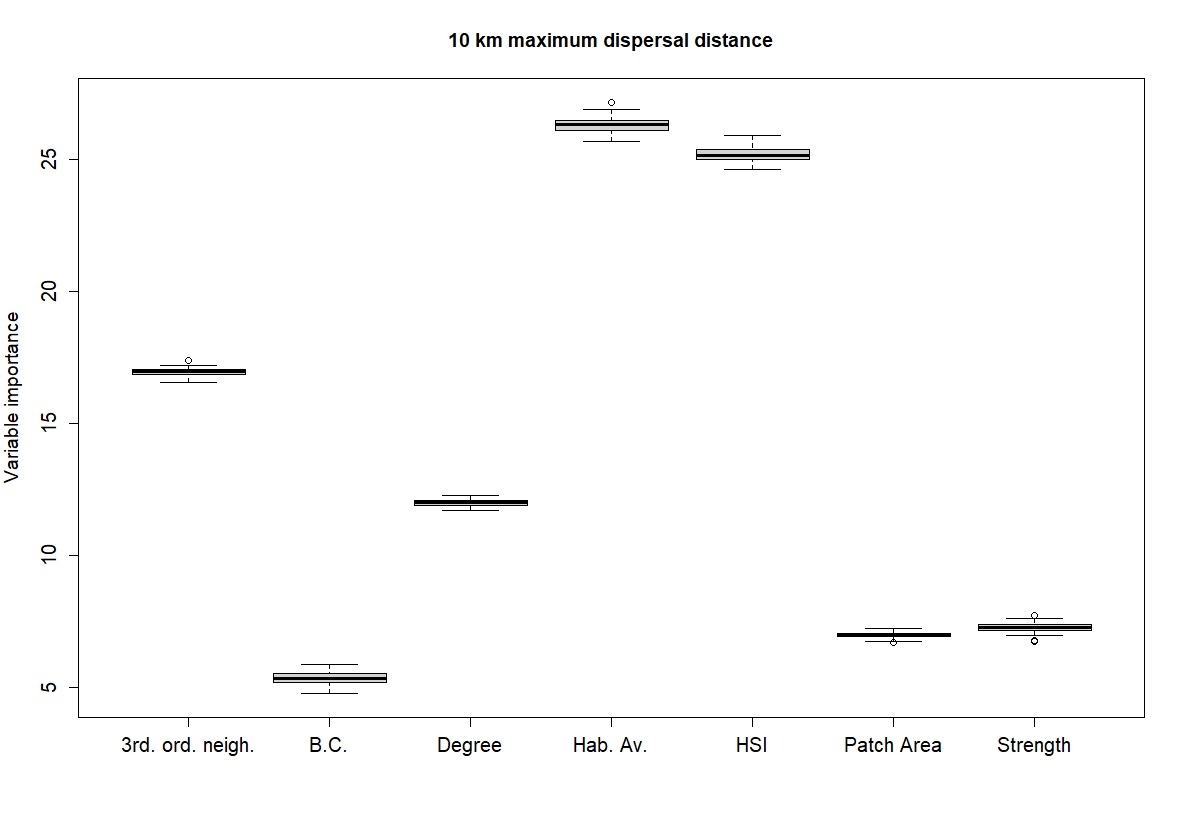


***Bombina variegata***

***
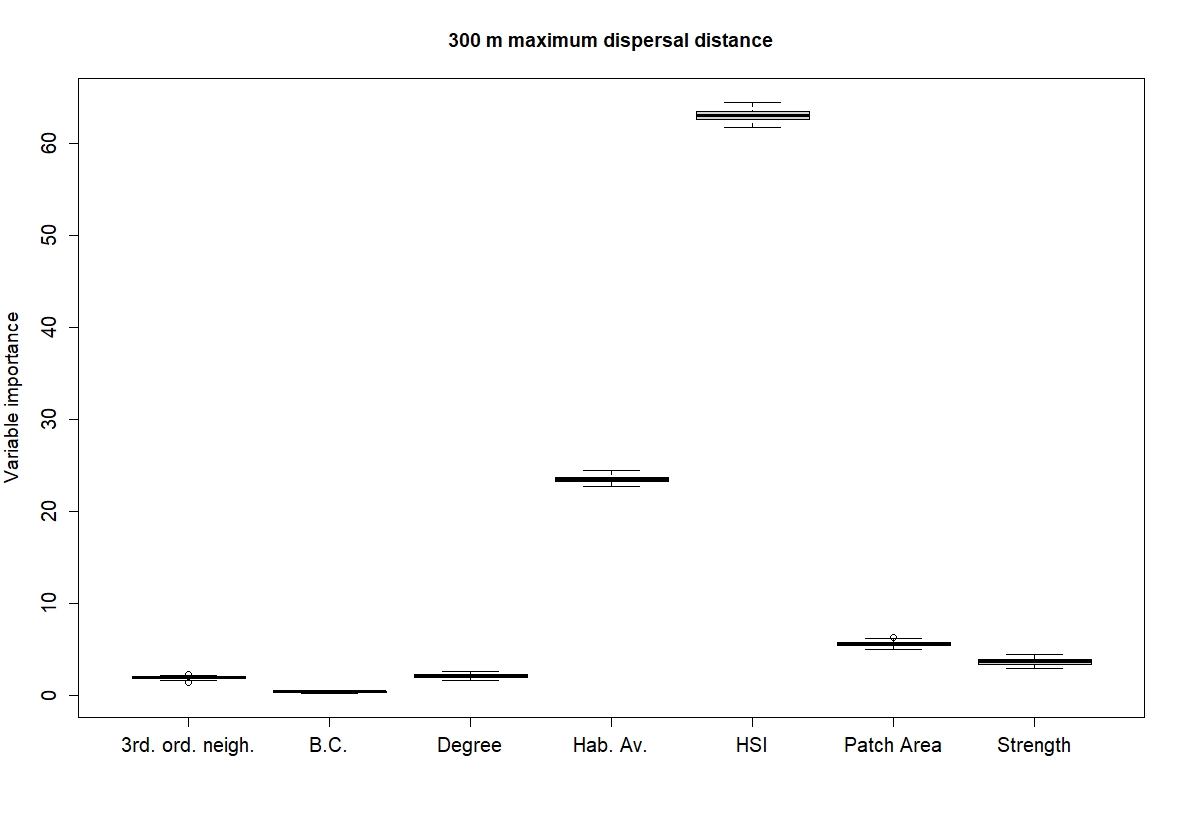
***

***
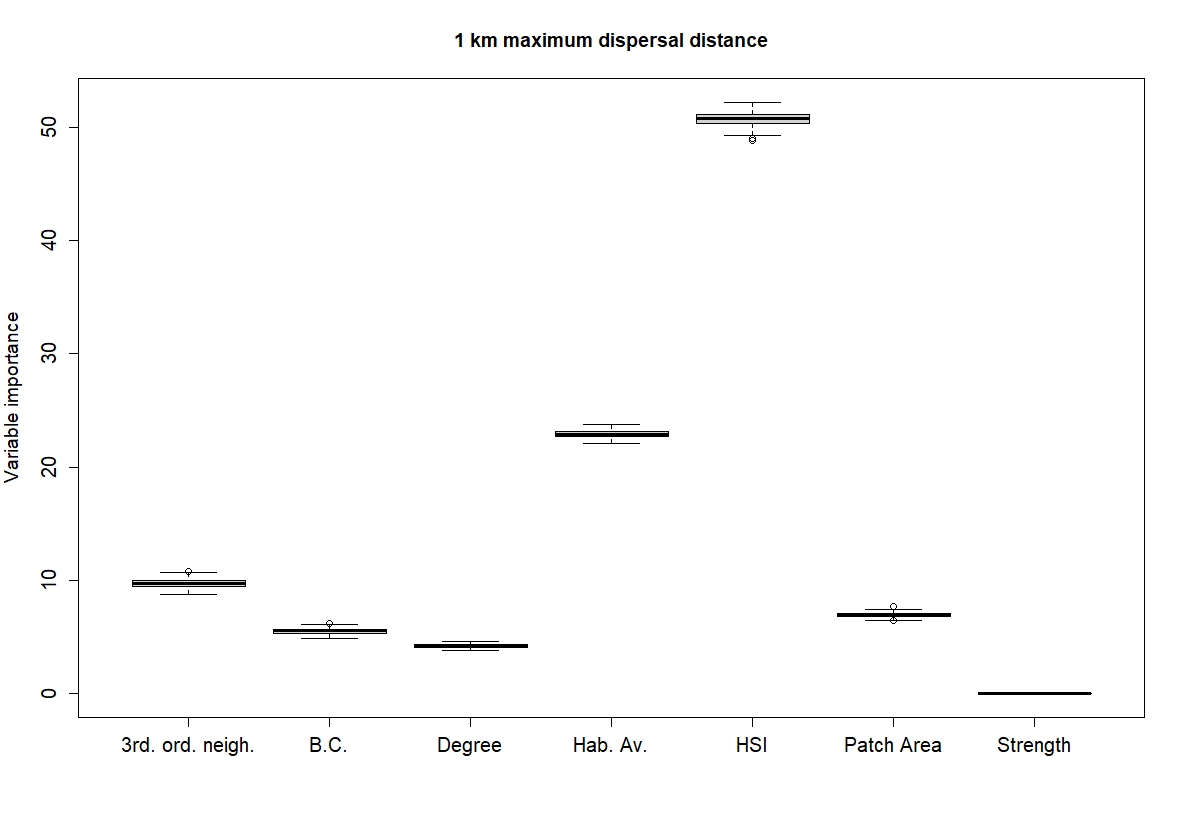
***

***Bombina variegata***

***
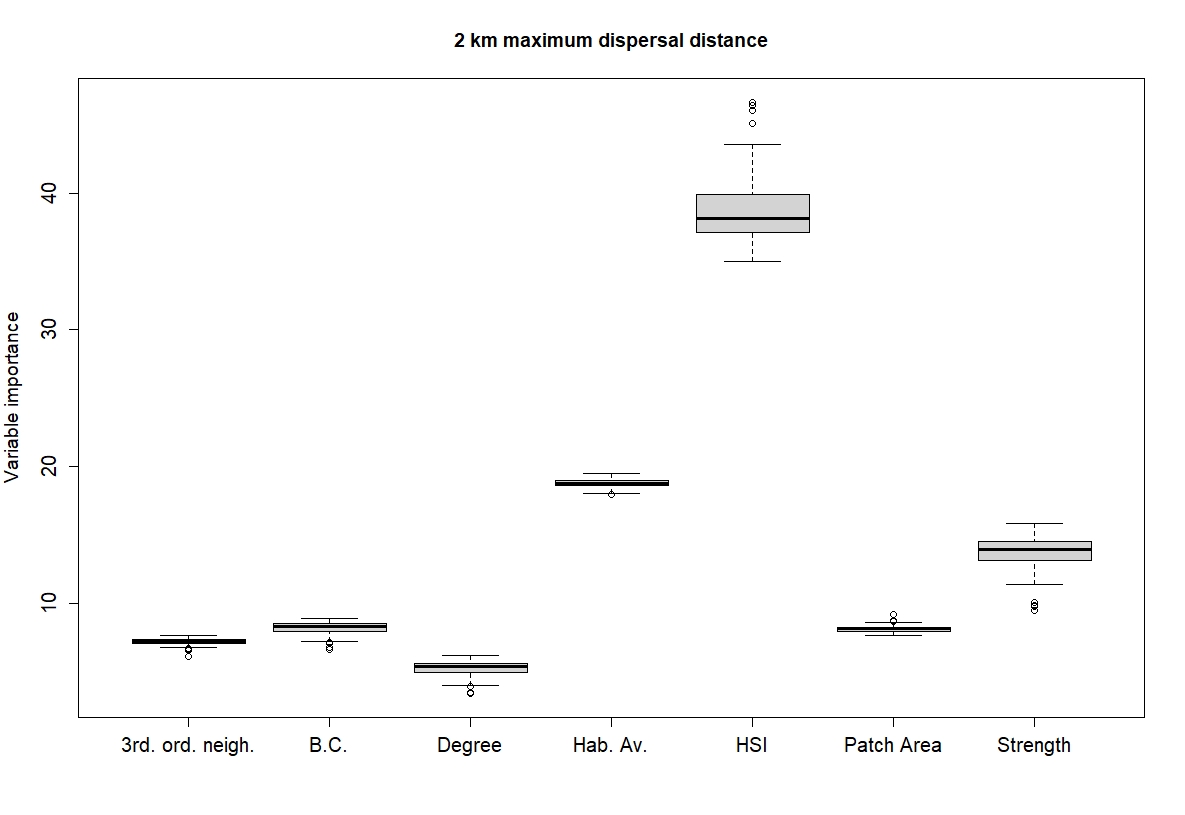

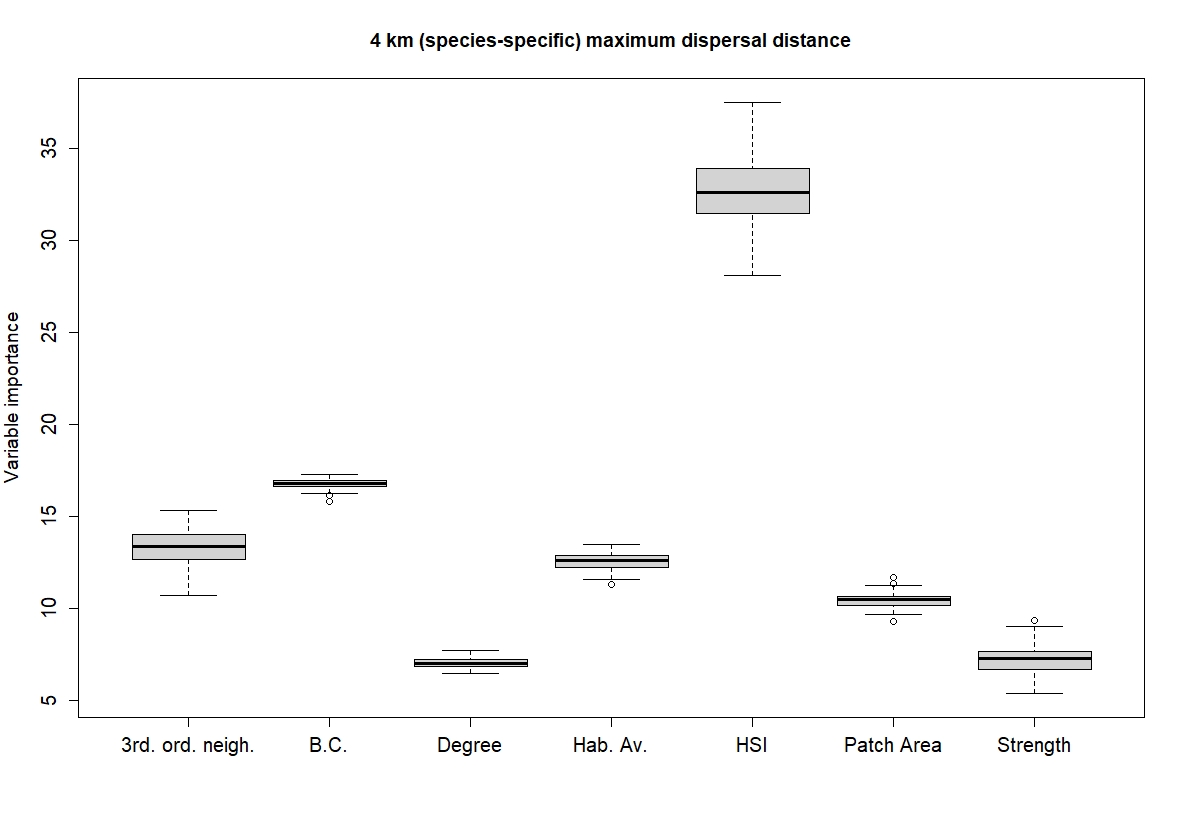
***

***Bombina variegata***

***
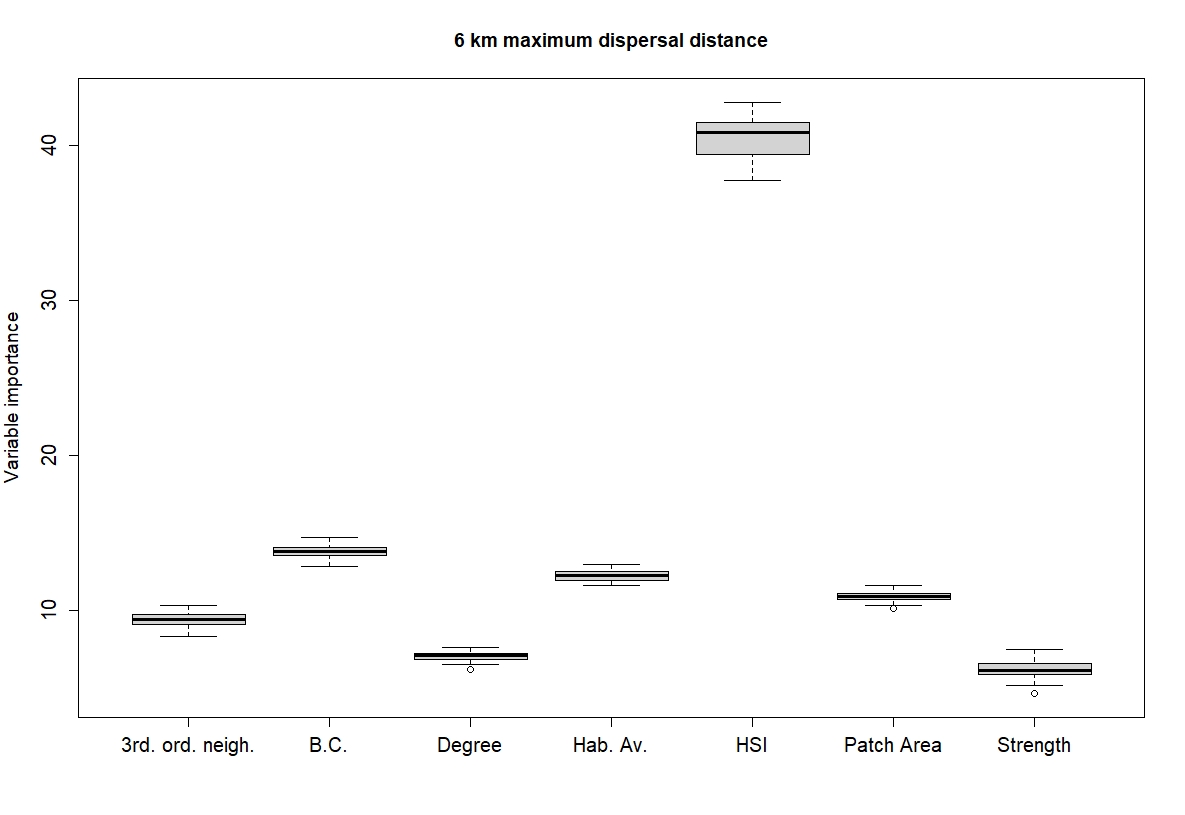

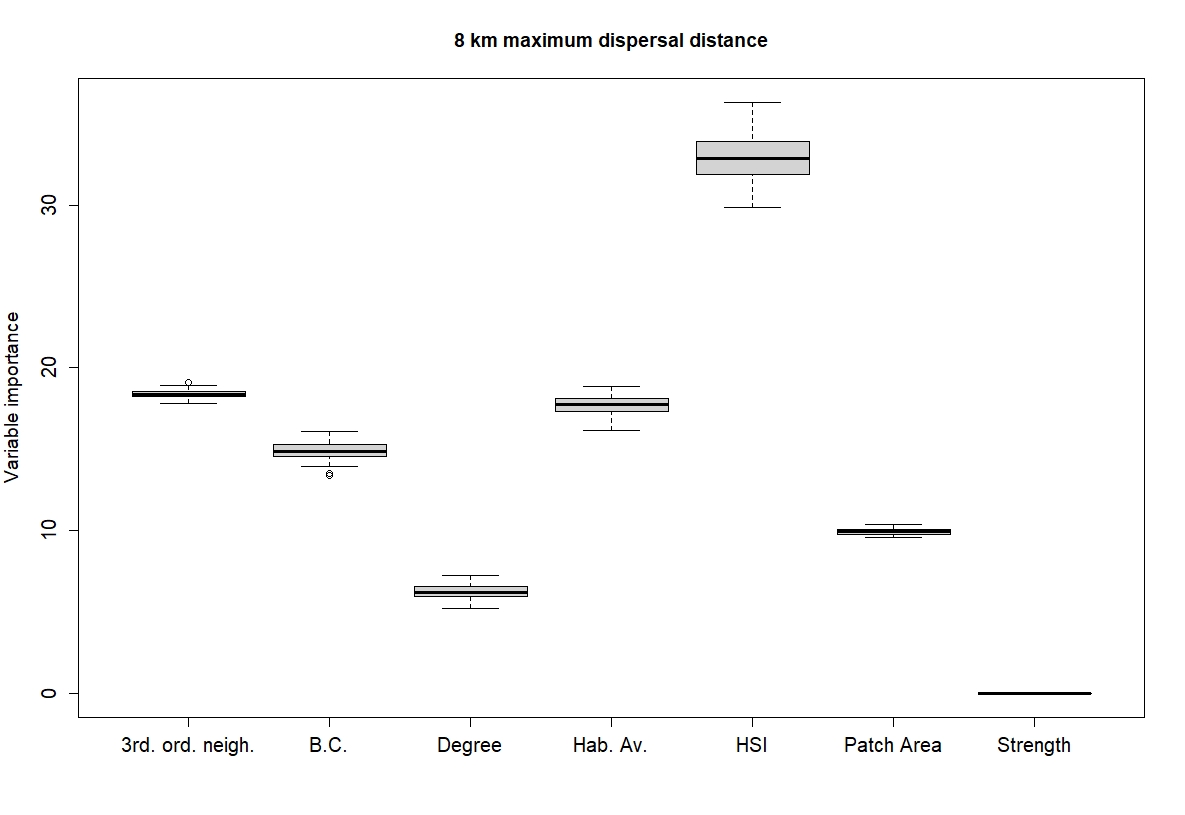
***

***Bombina variegata***

***
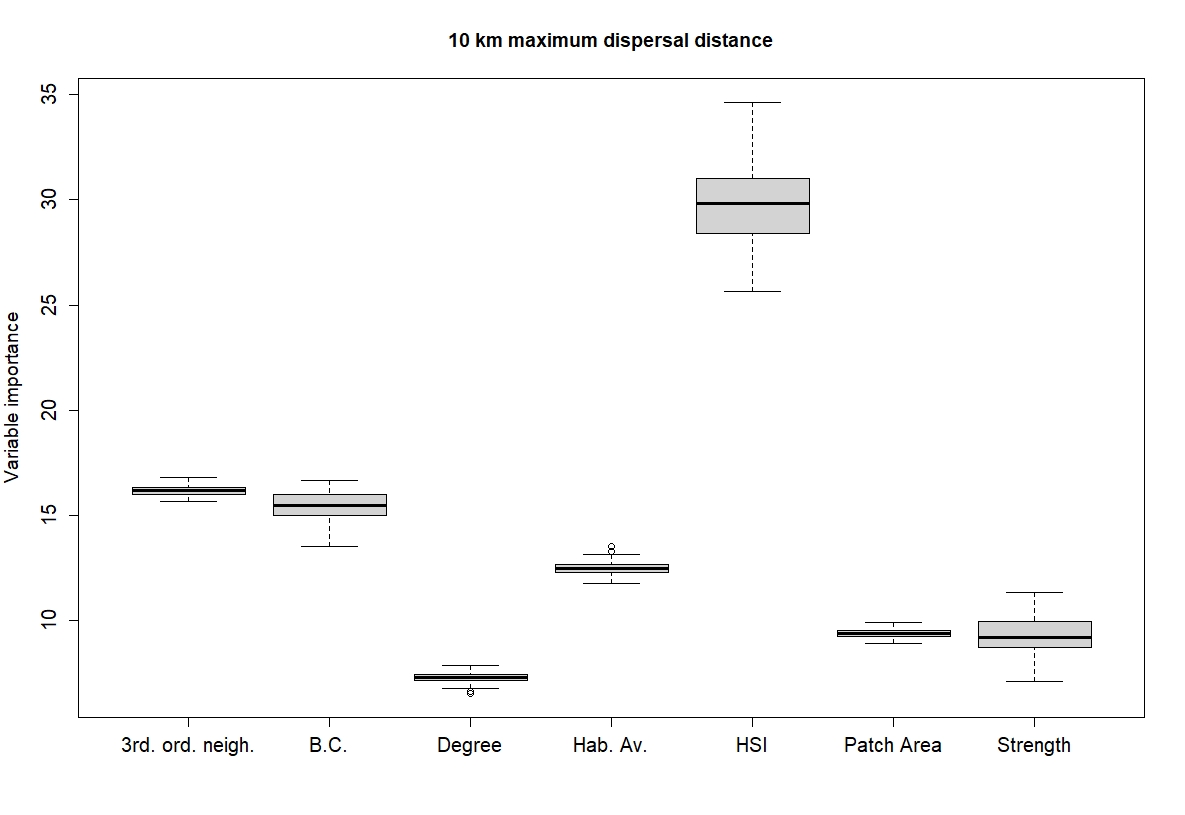
***

***Epidalea calamita***


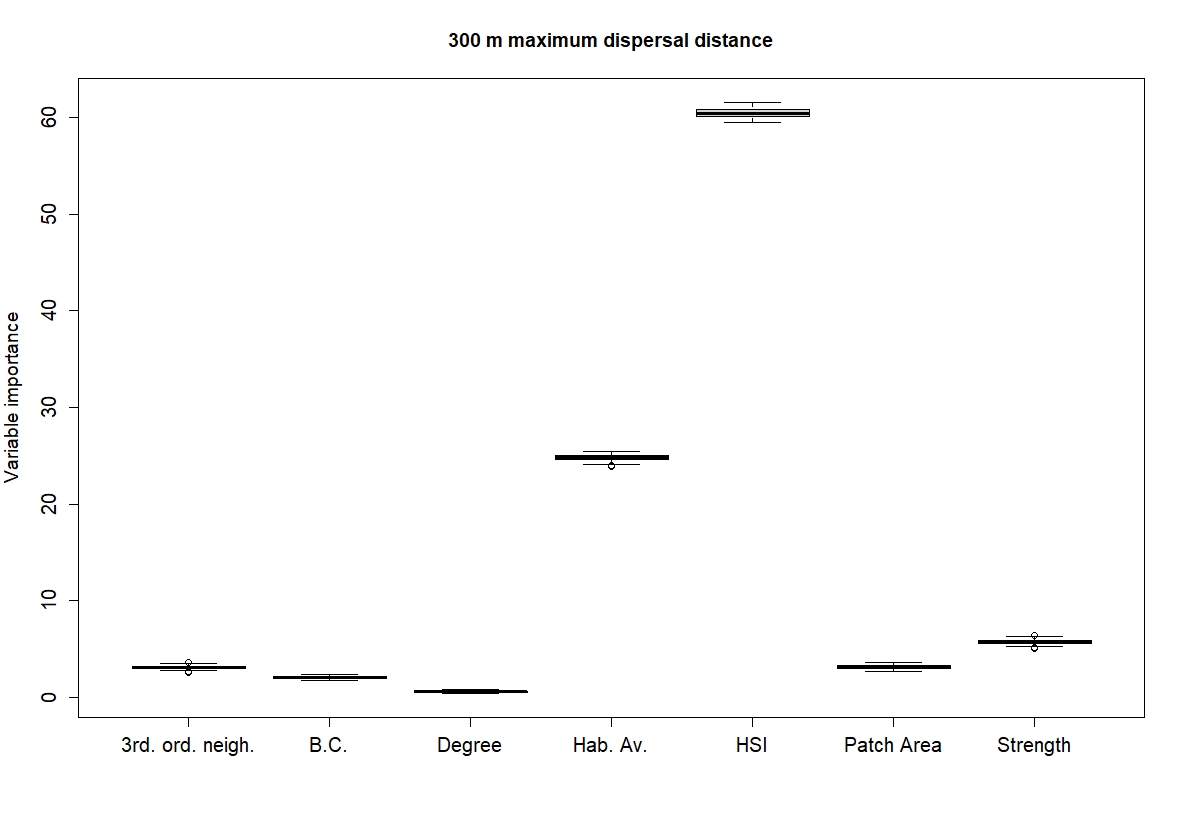

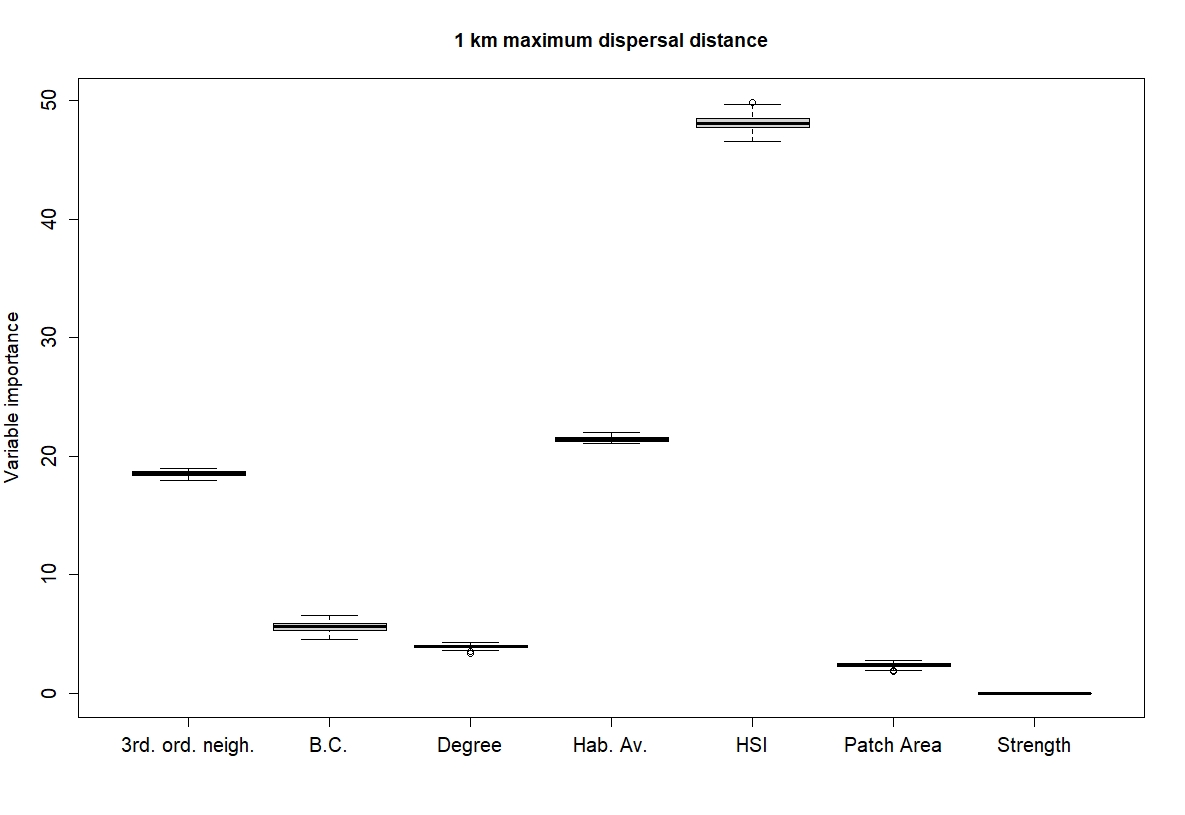


***Epidalea calamita***


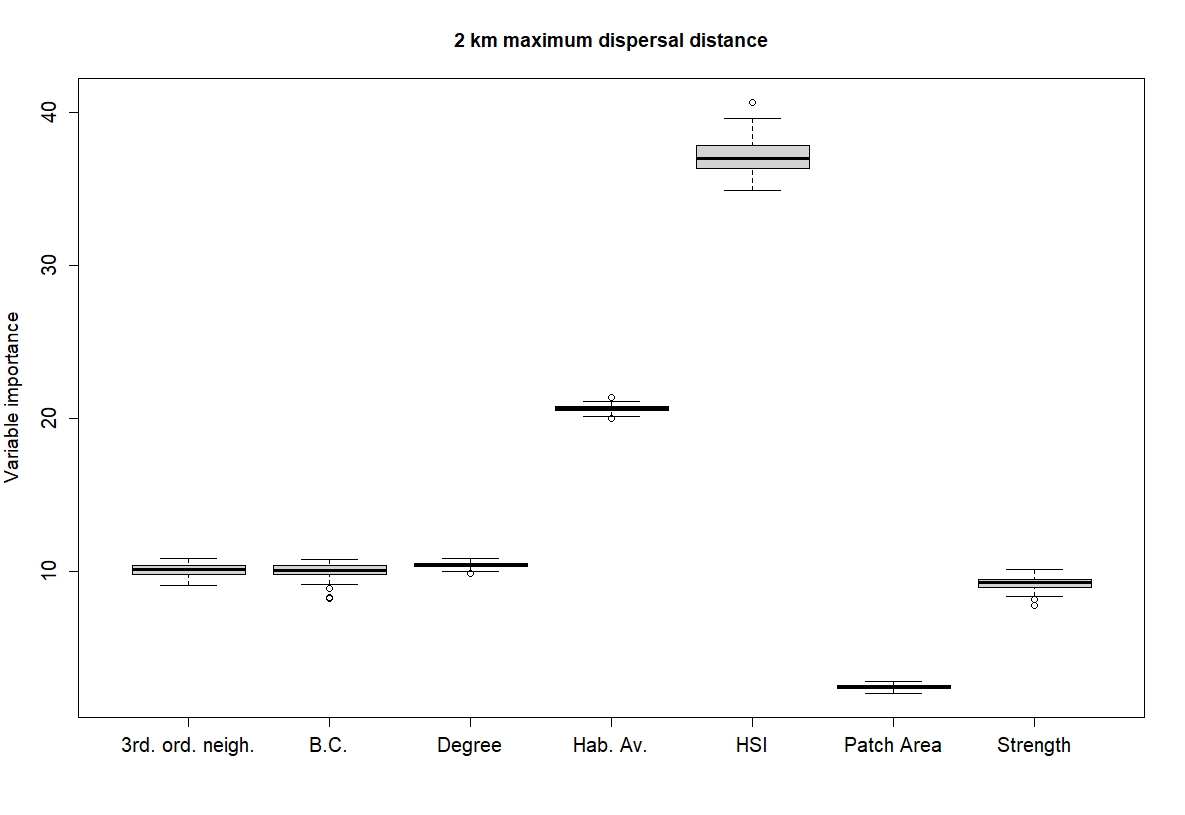

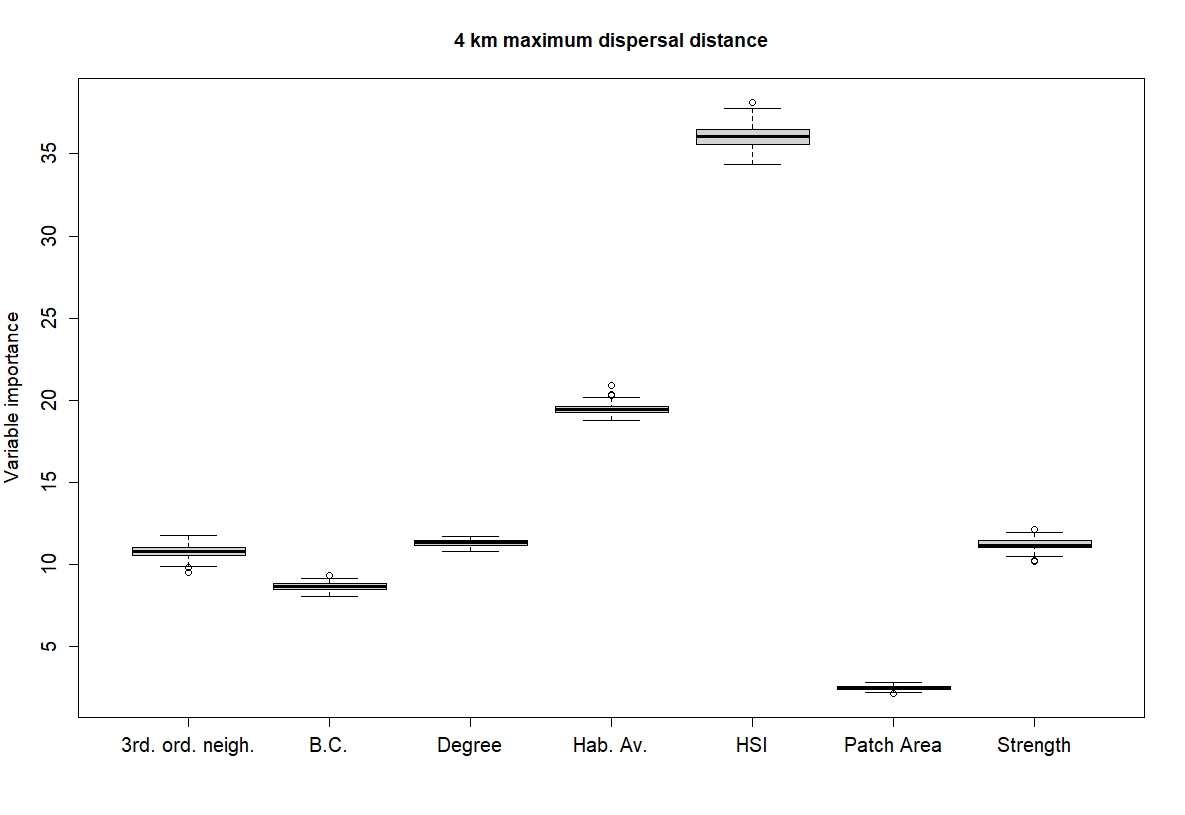


***Epidalea calamita***


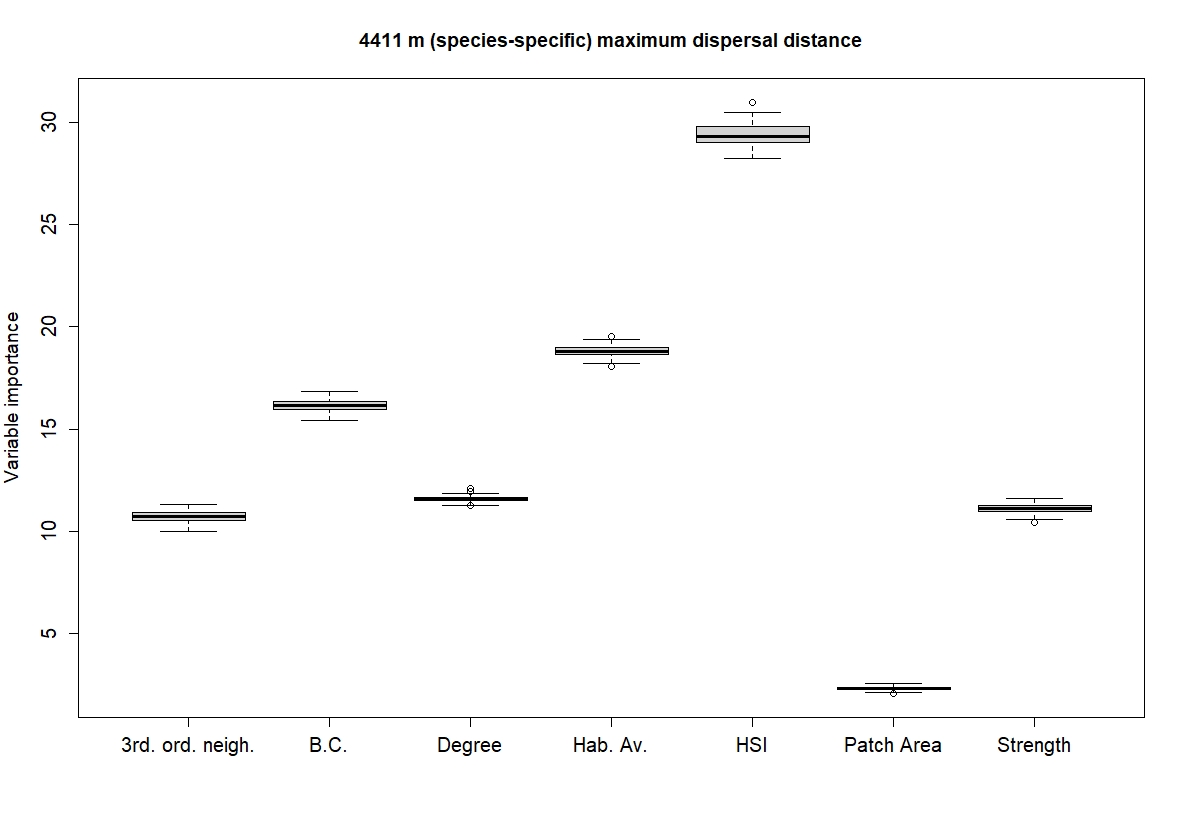

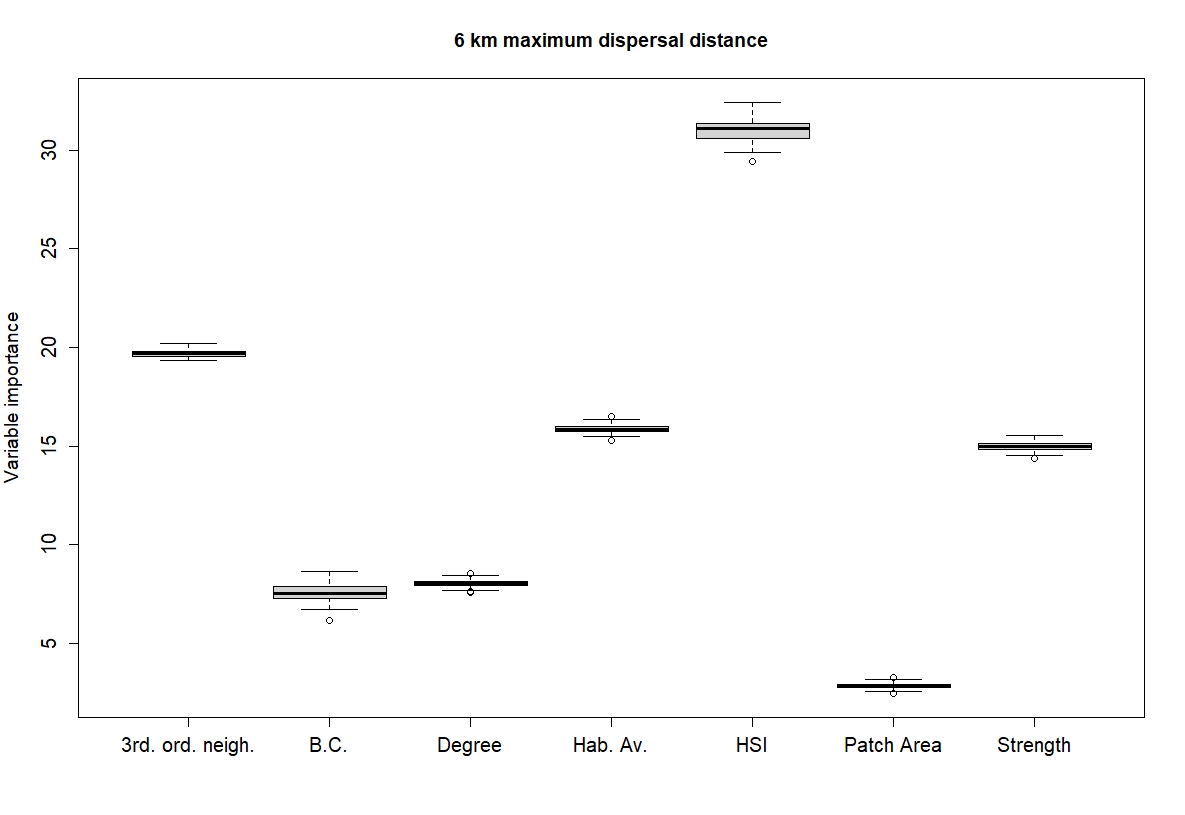


***Epidalea calamita***


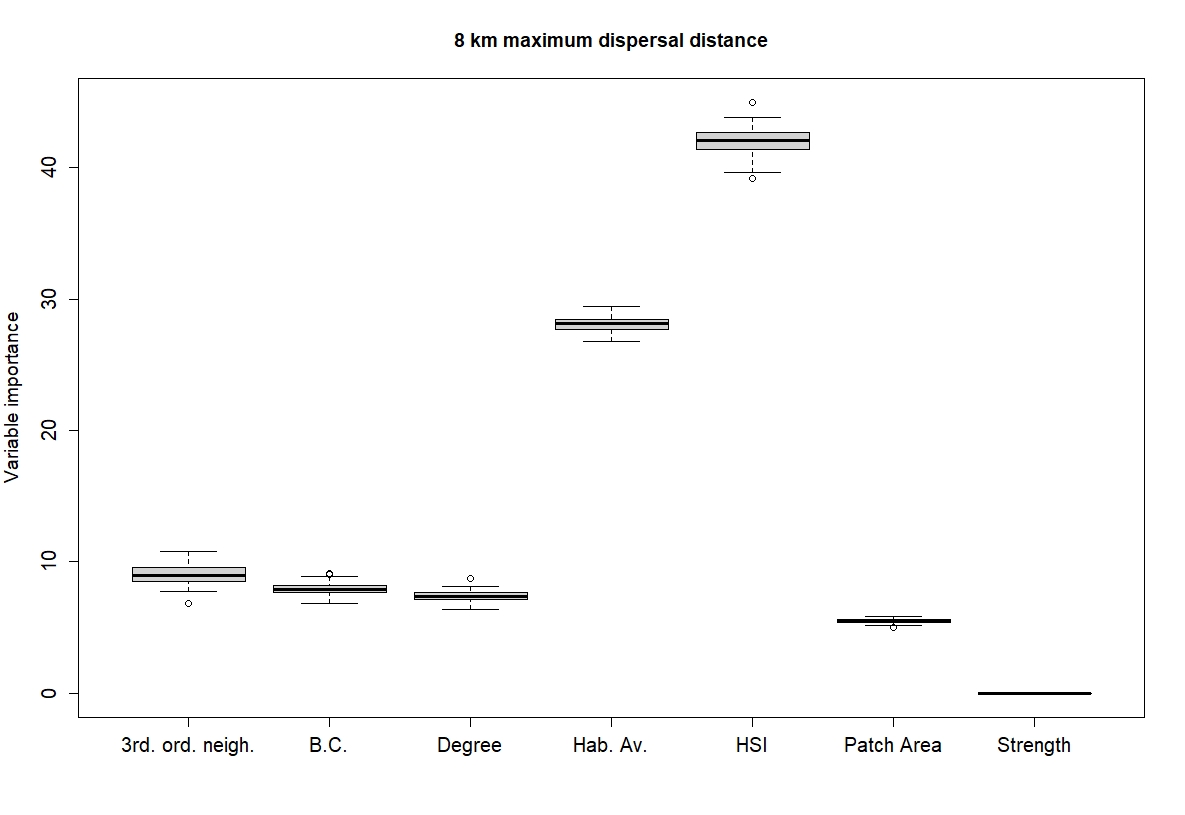

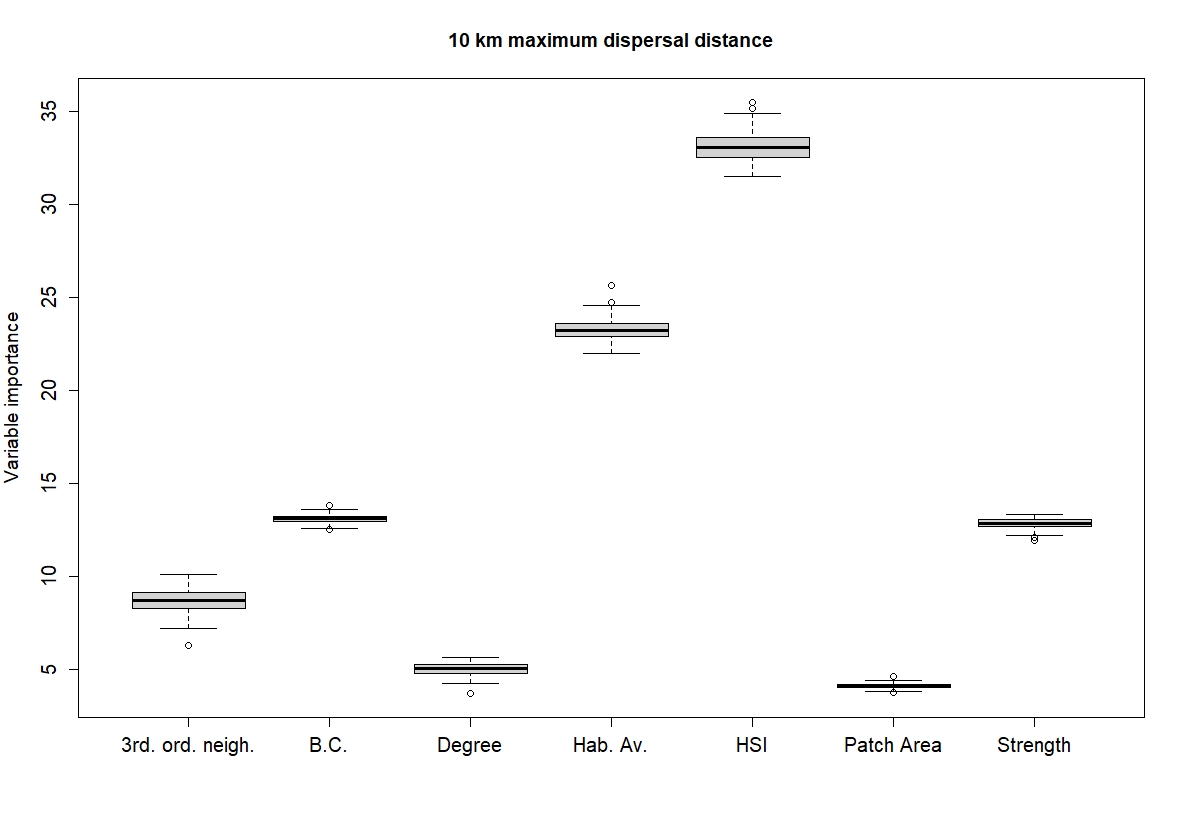


***Hyla arborea***


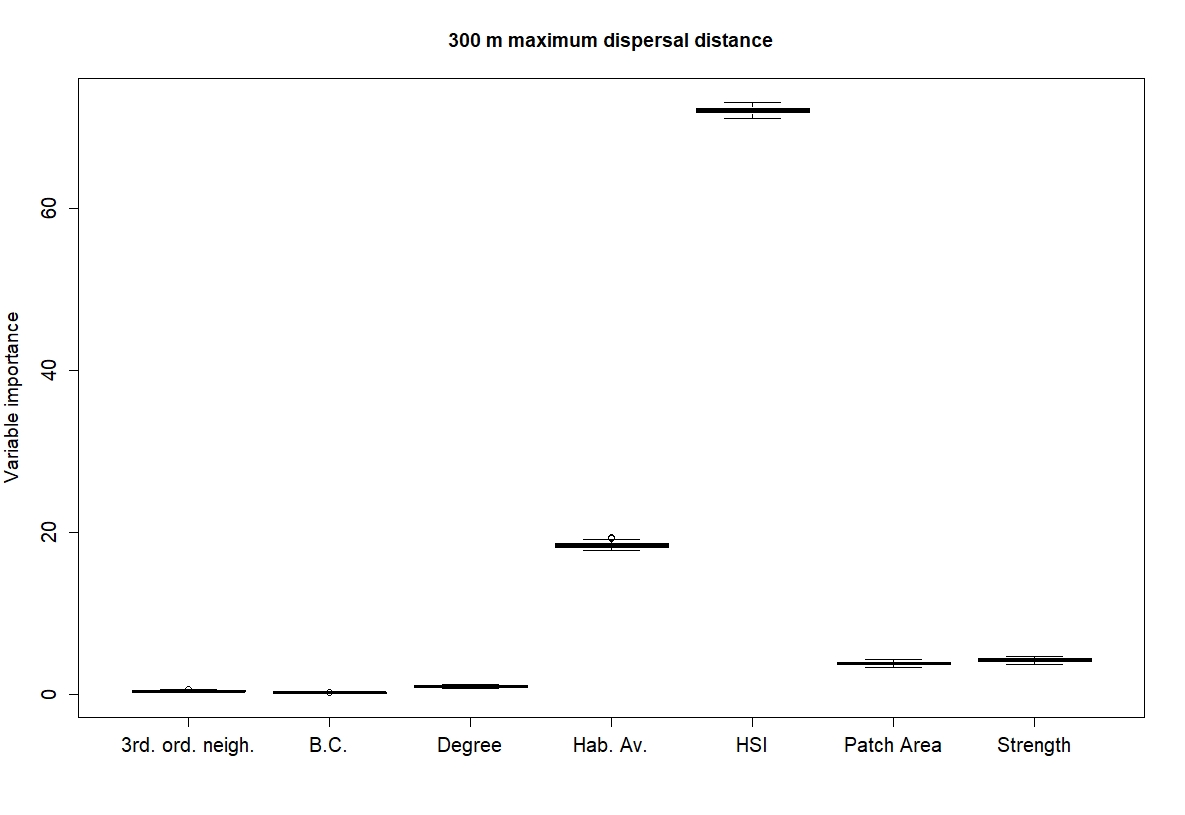

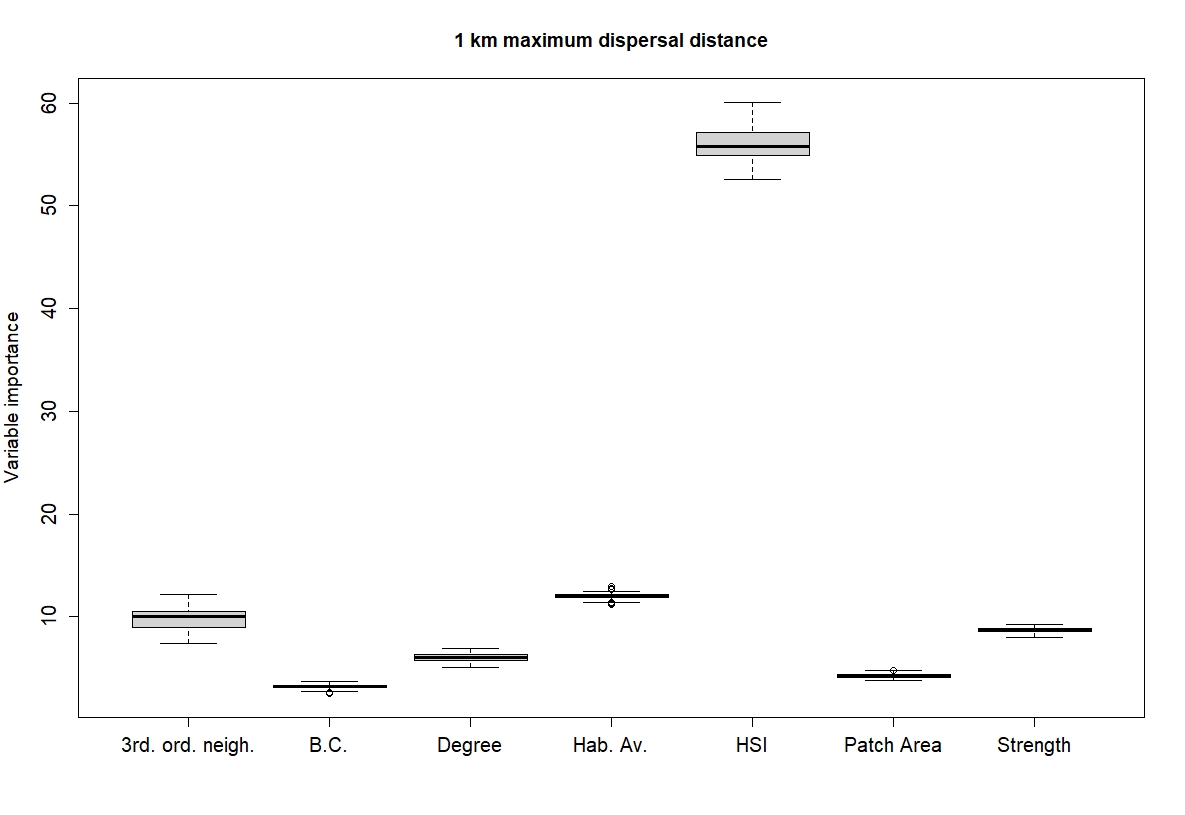


***Hyla arborea***


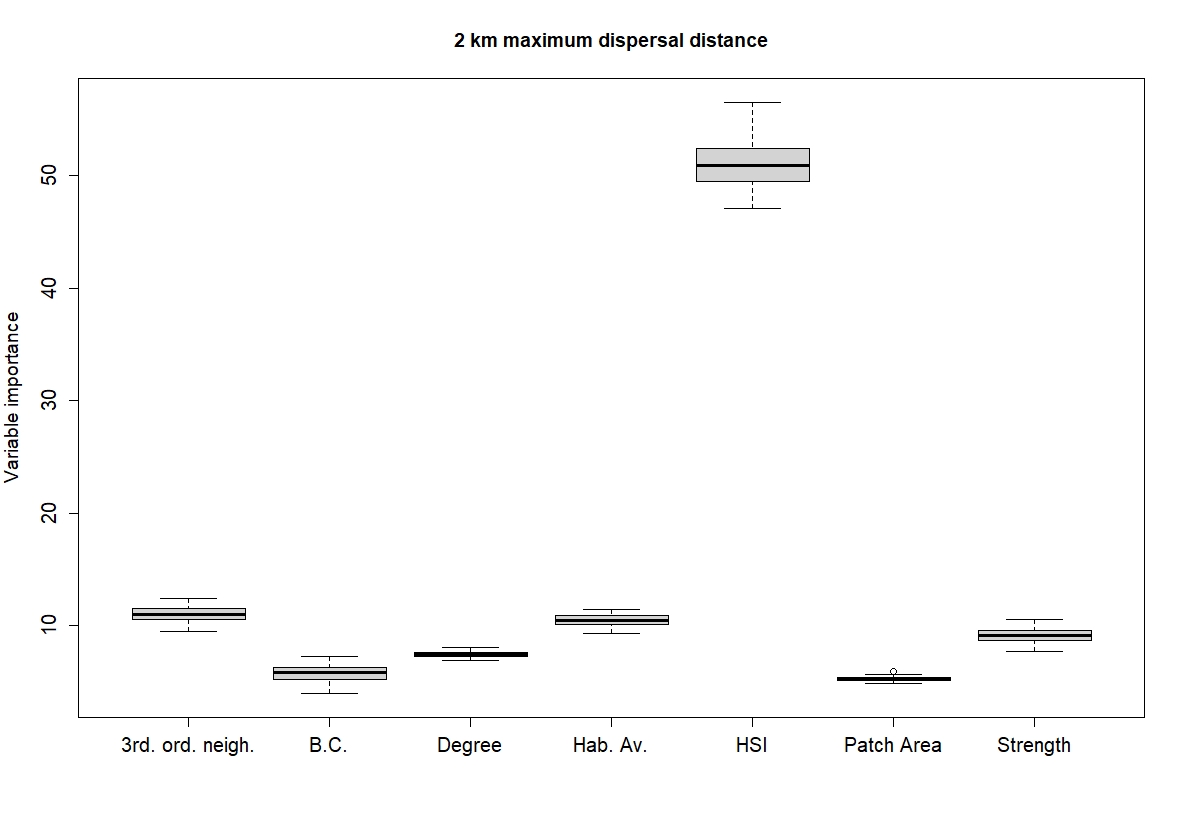


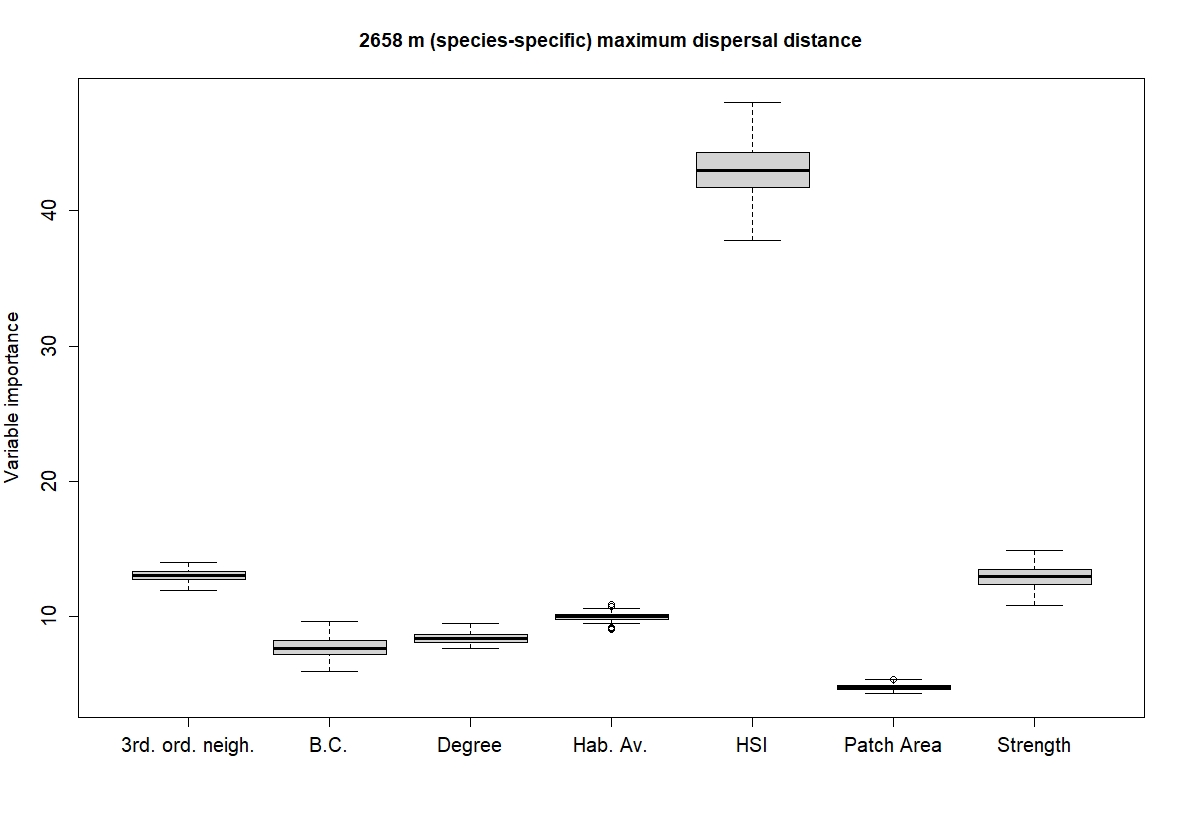


***Hyla arborea***


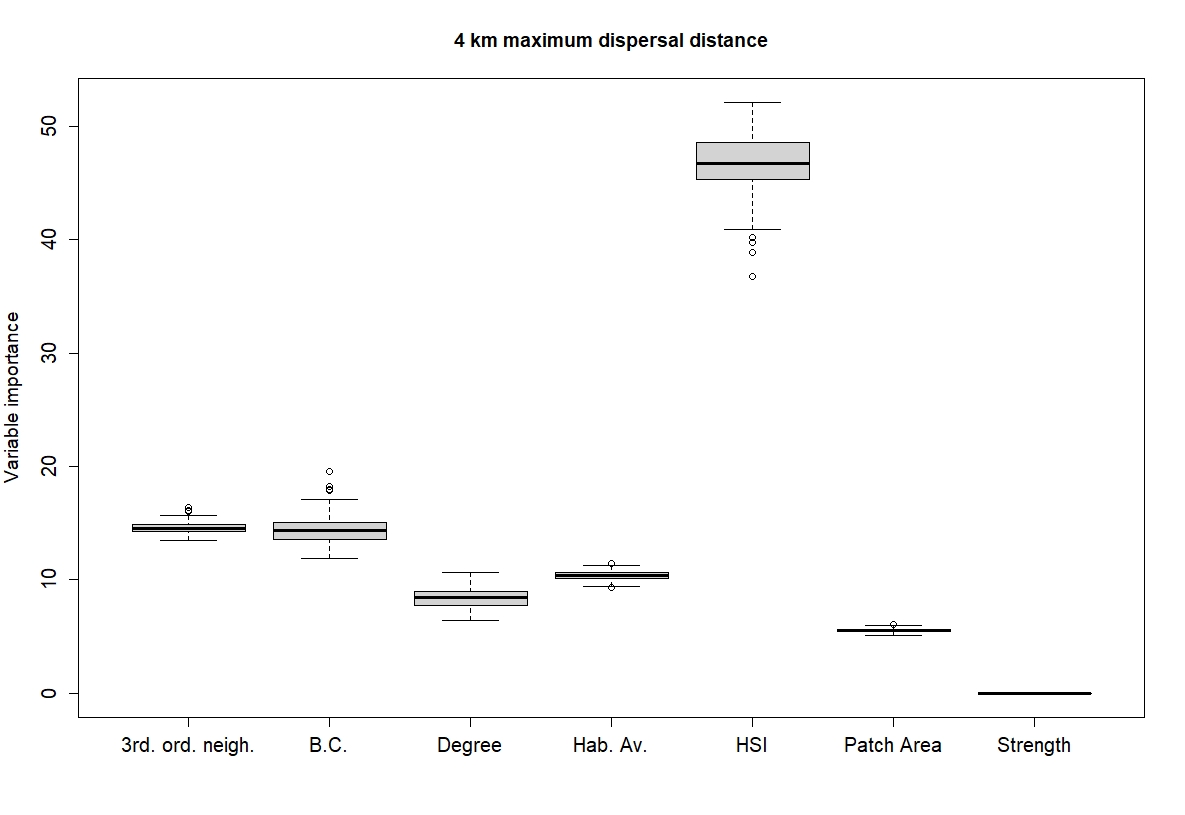

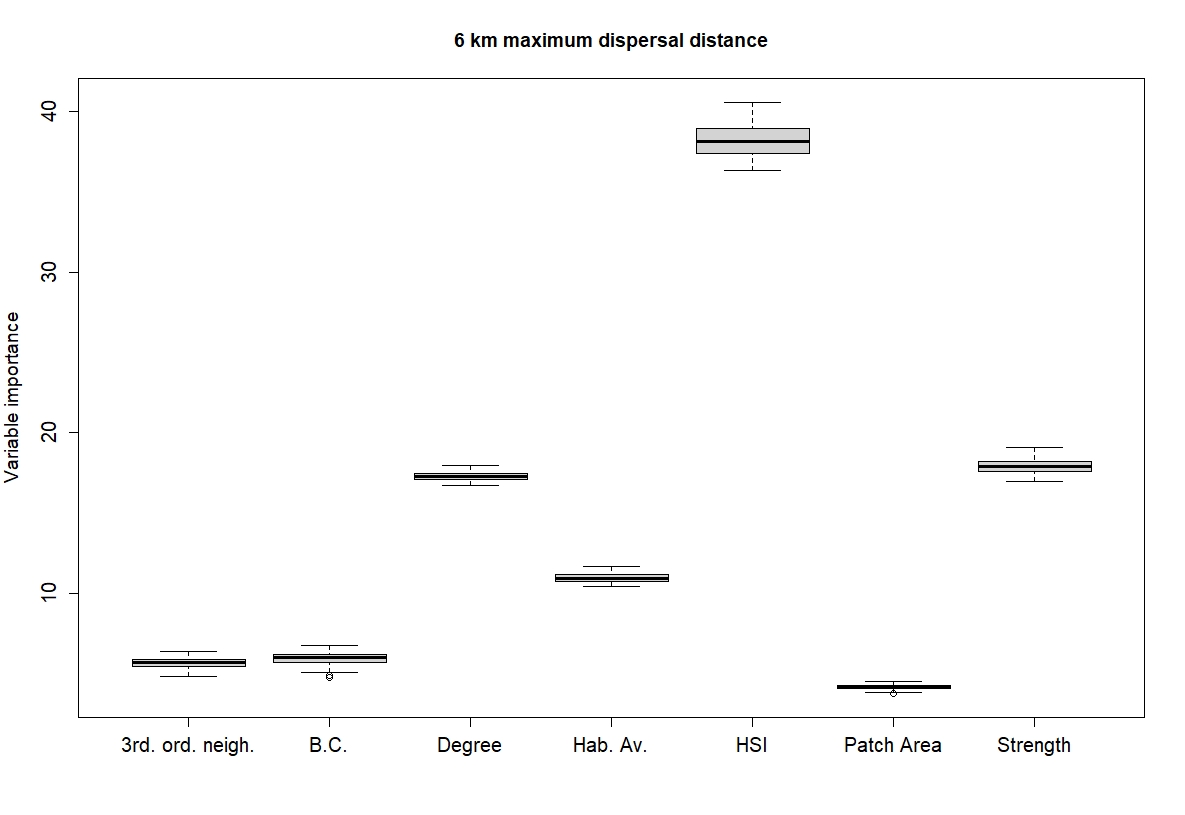


***Hyla arborea***


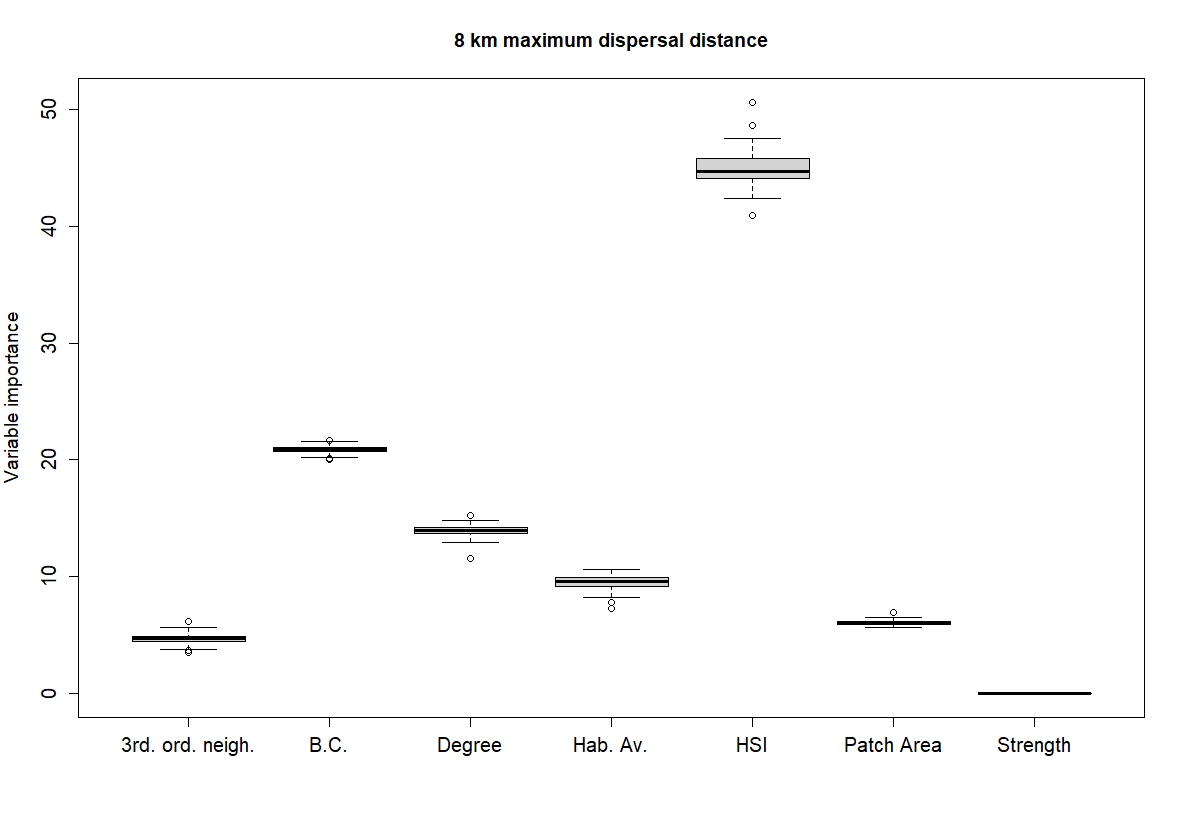

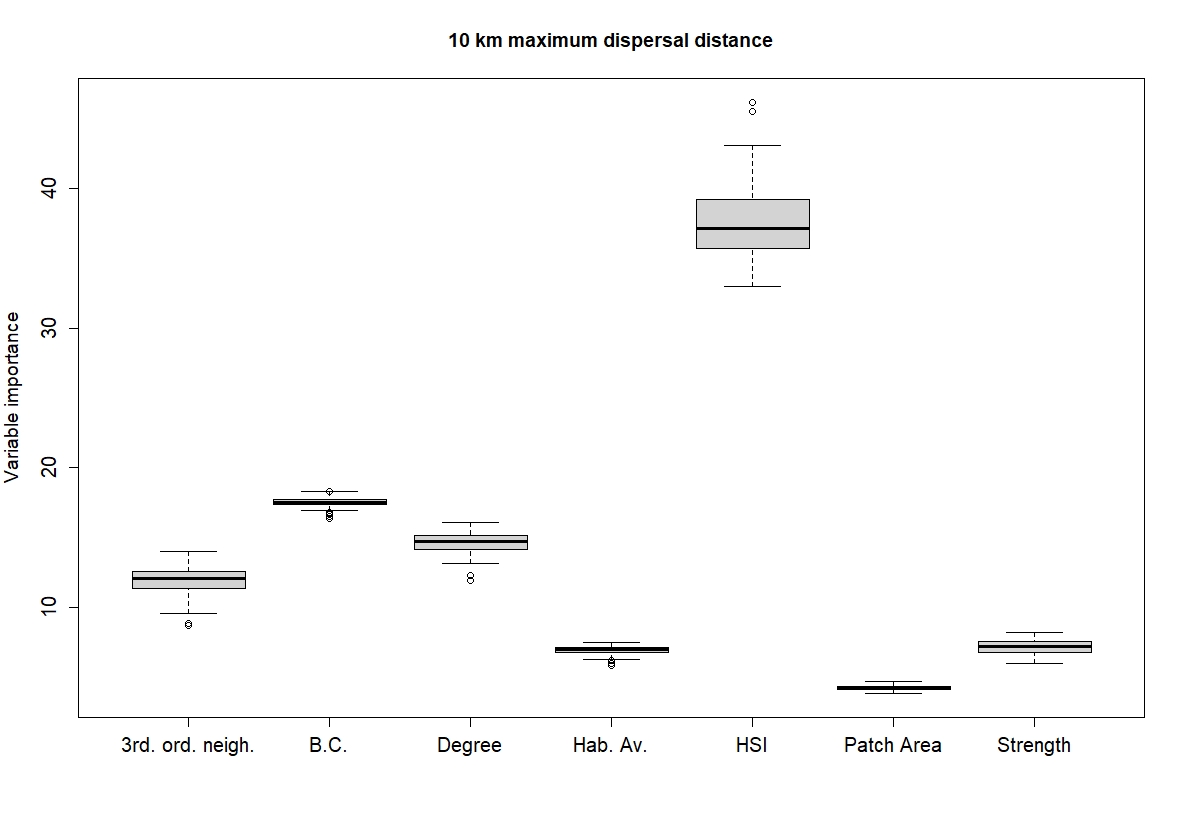


***Pelophylax lessonae* agg**
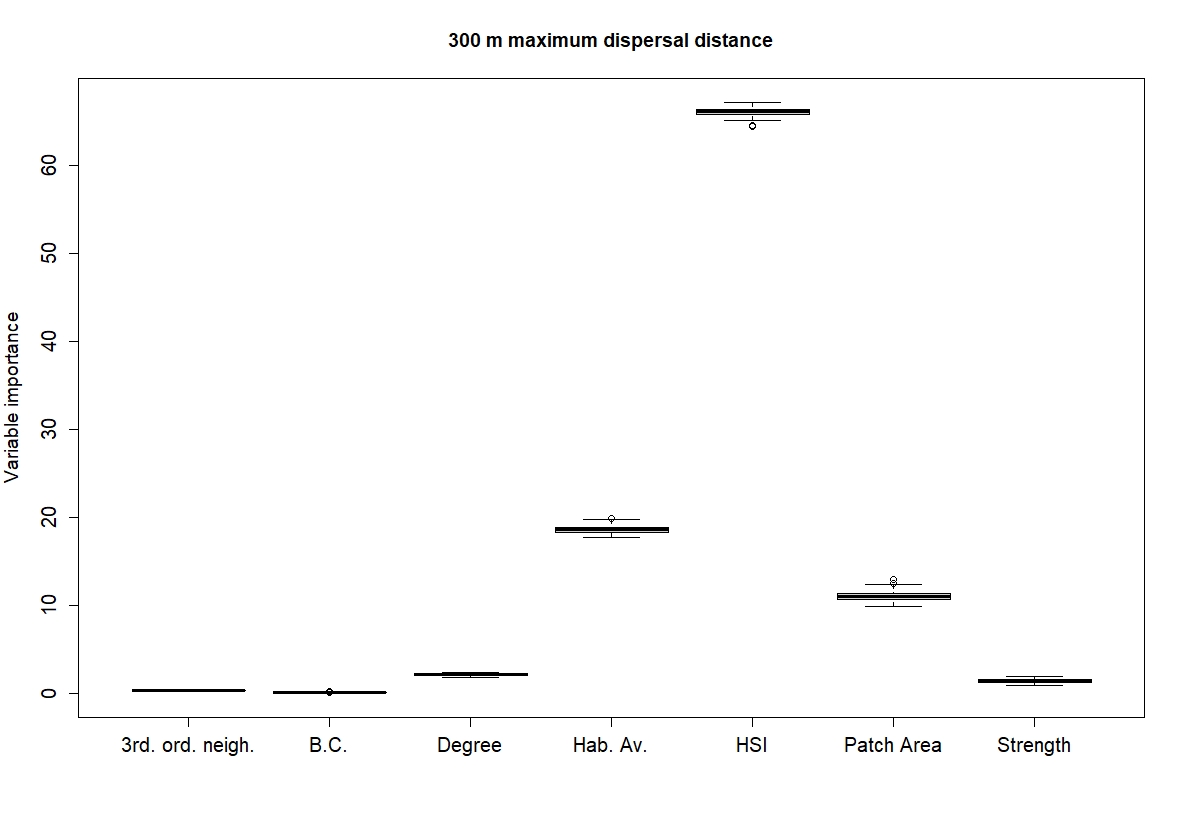


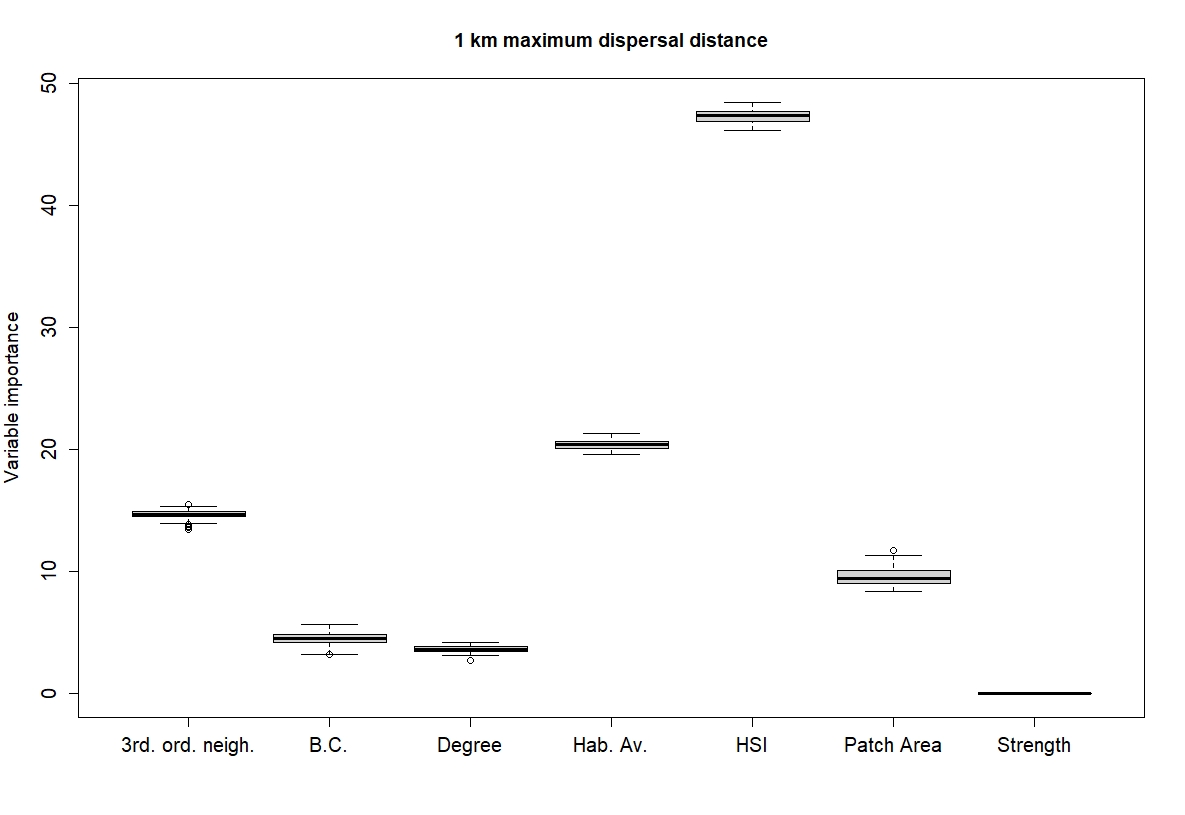


***Pelophylax lessonae* agg**


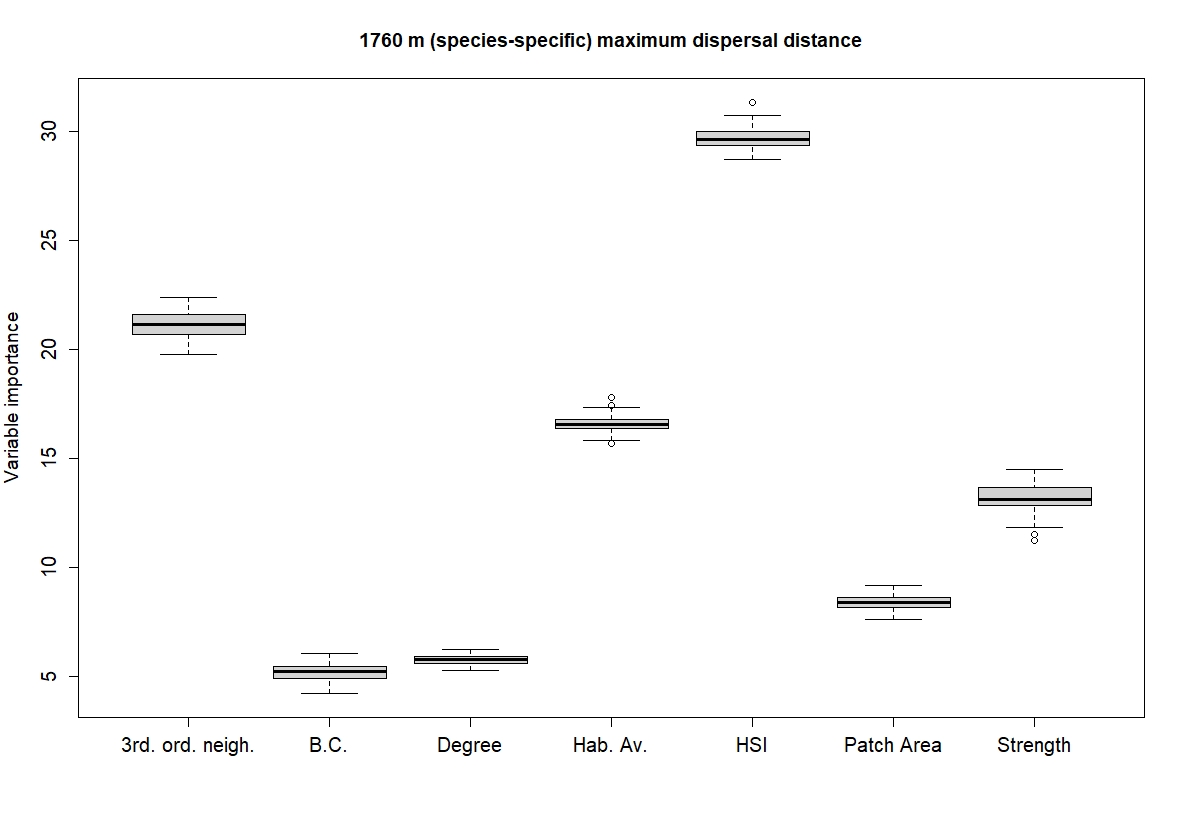


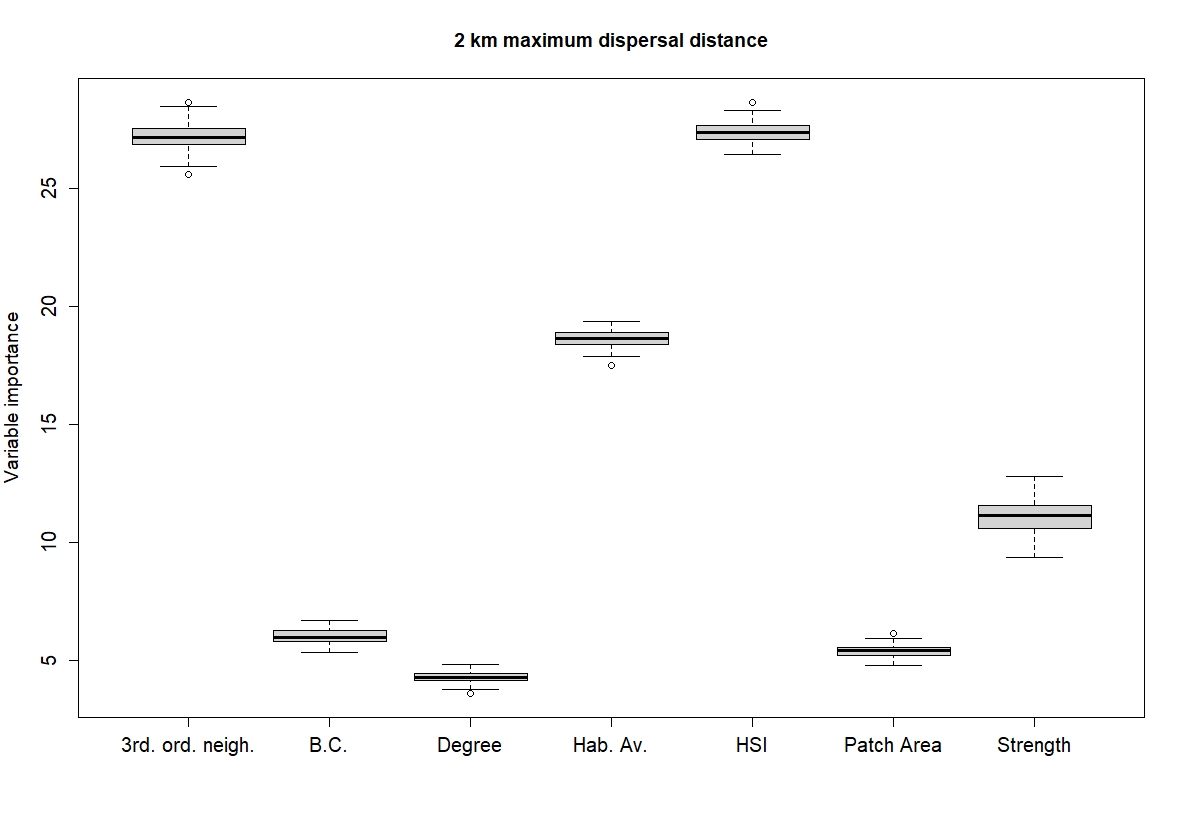


***Pelophylax lessonae* agg**


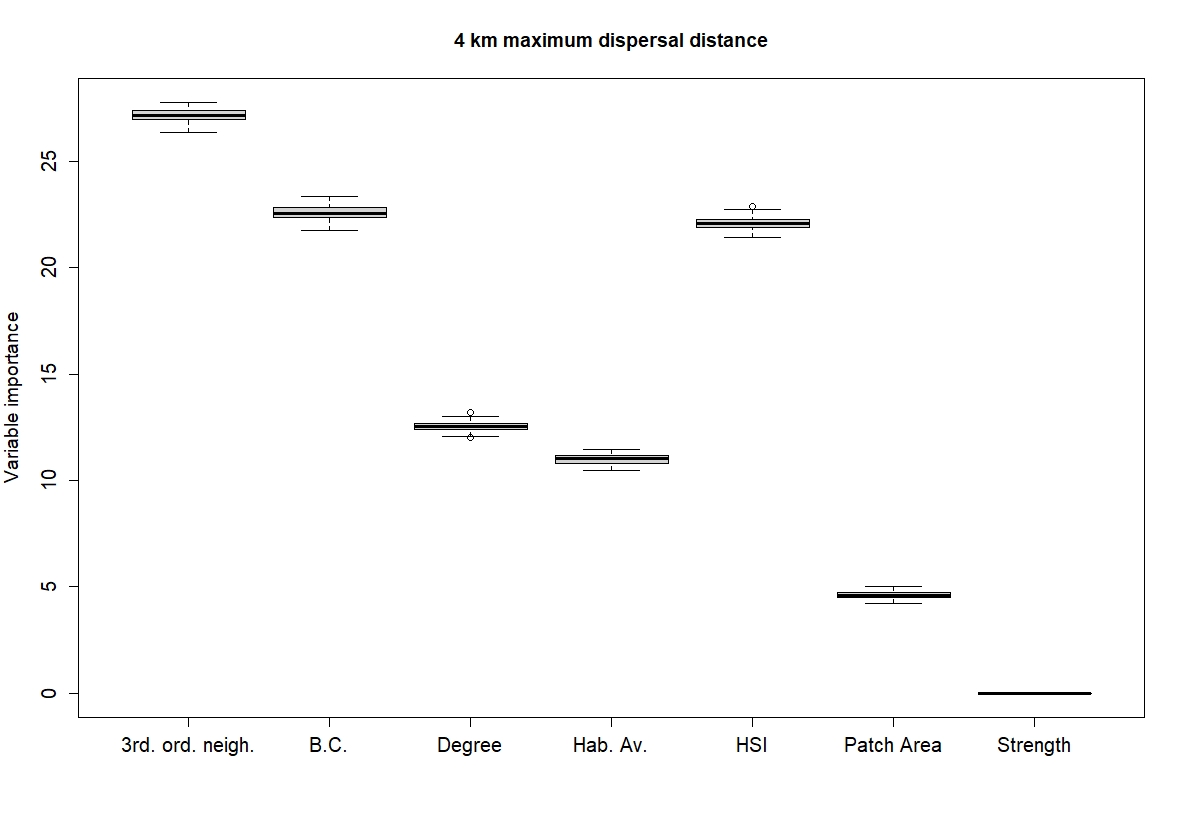

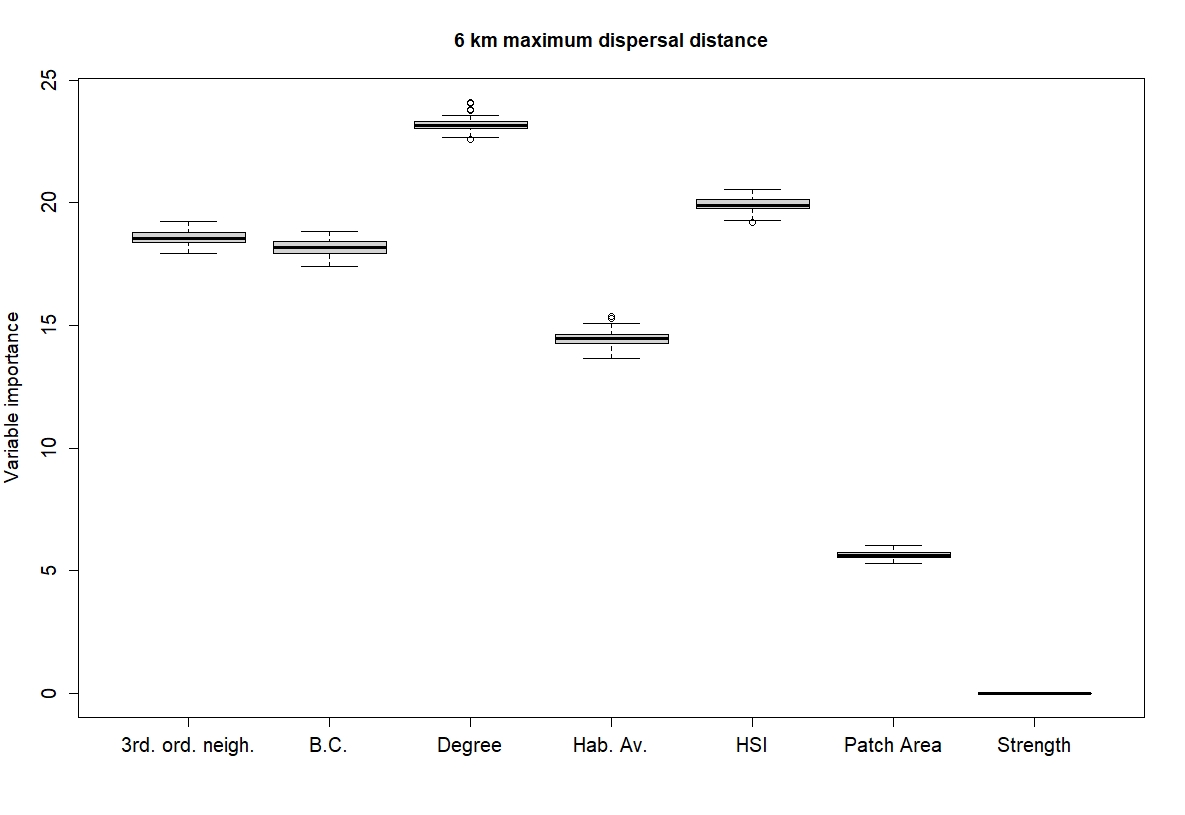


***Pelophylax lessonae* agg**


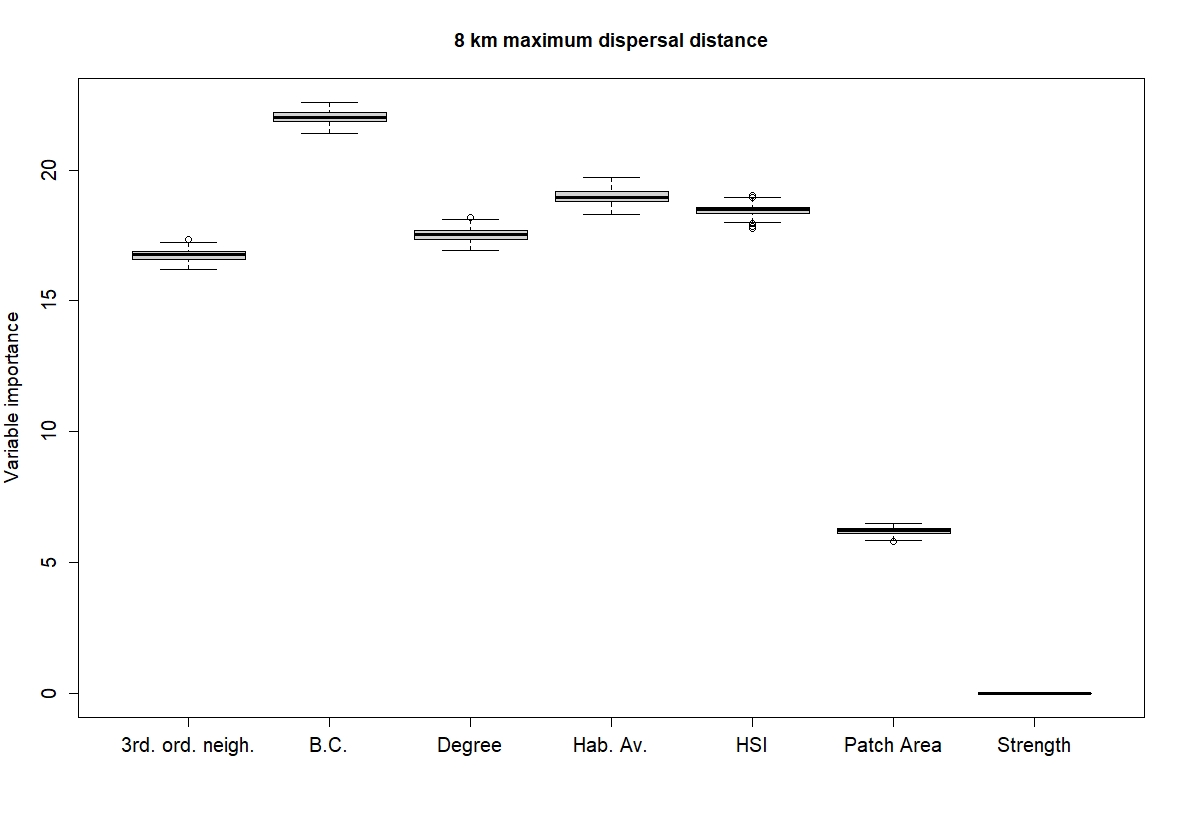

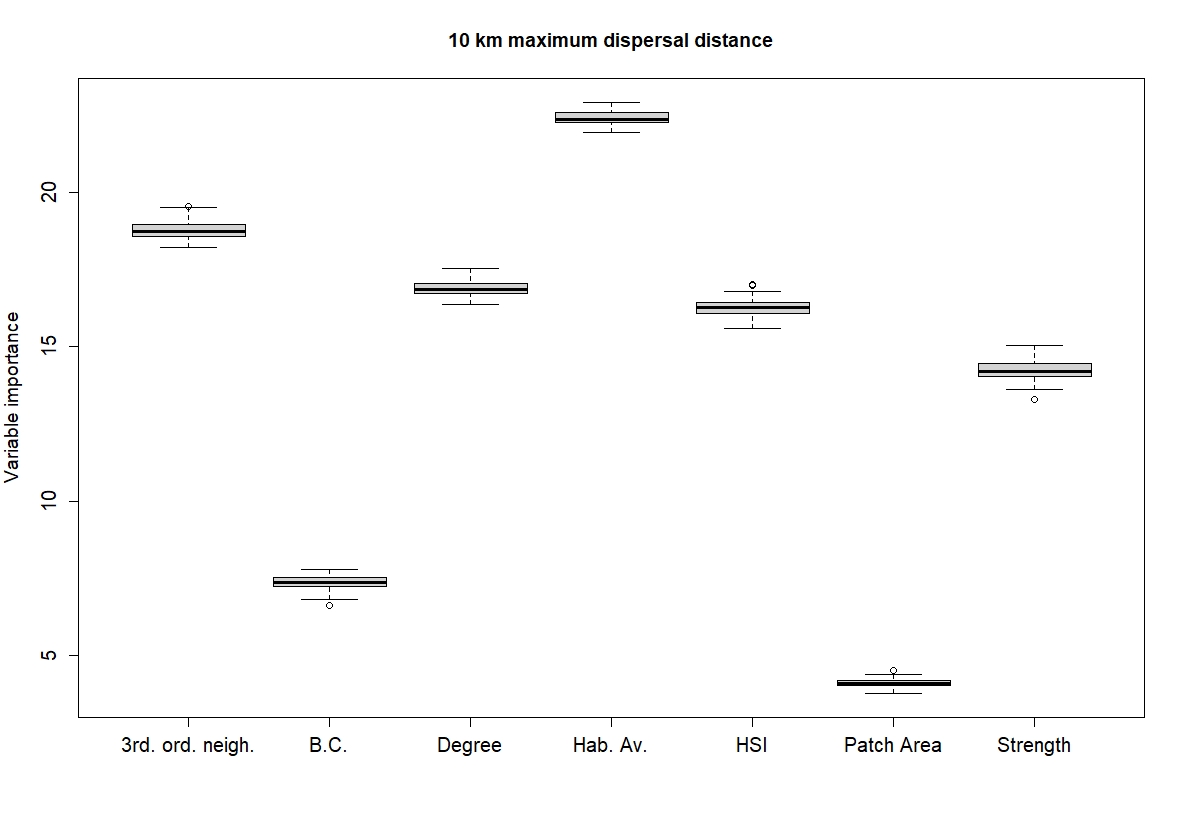


***Pelophylax ridibundus***


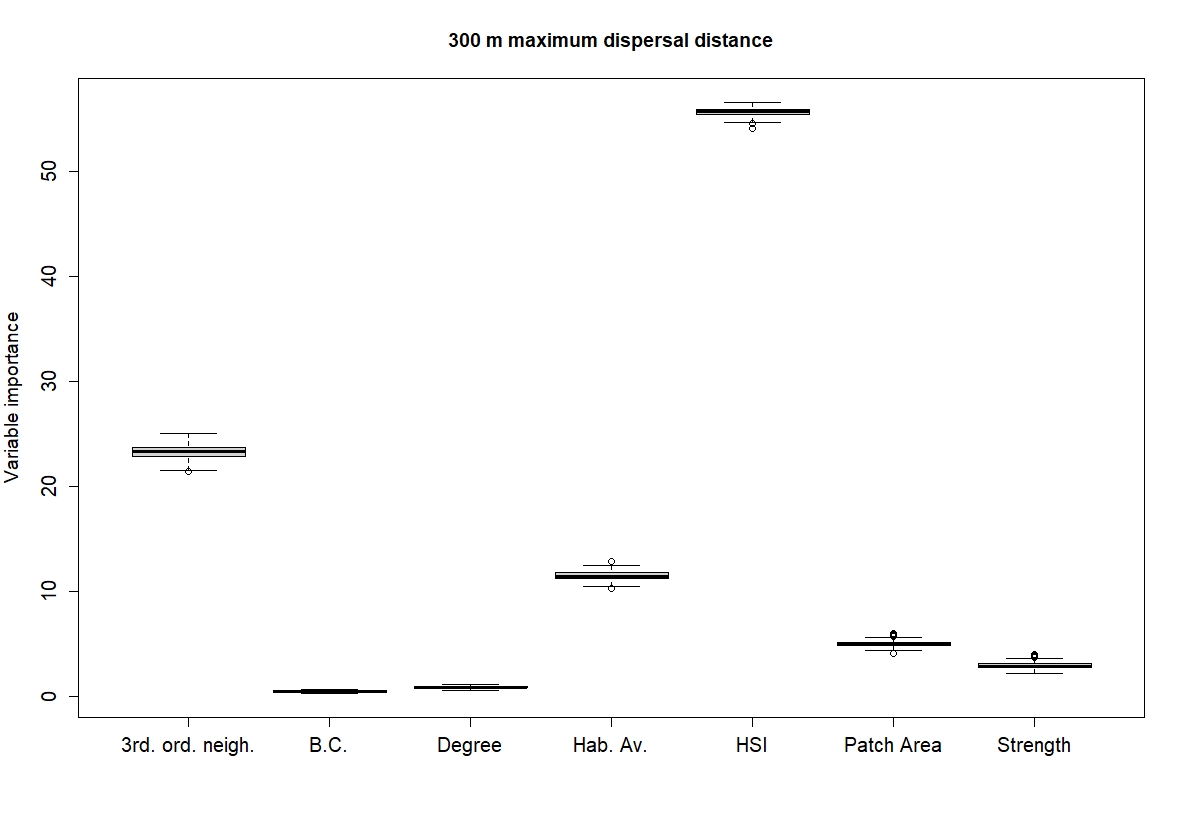

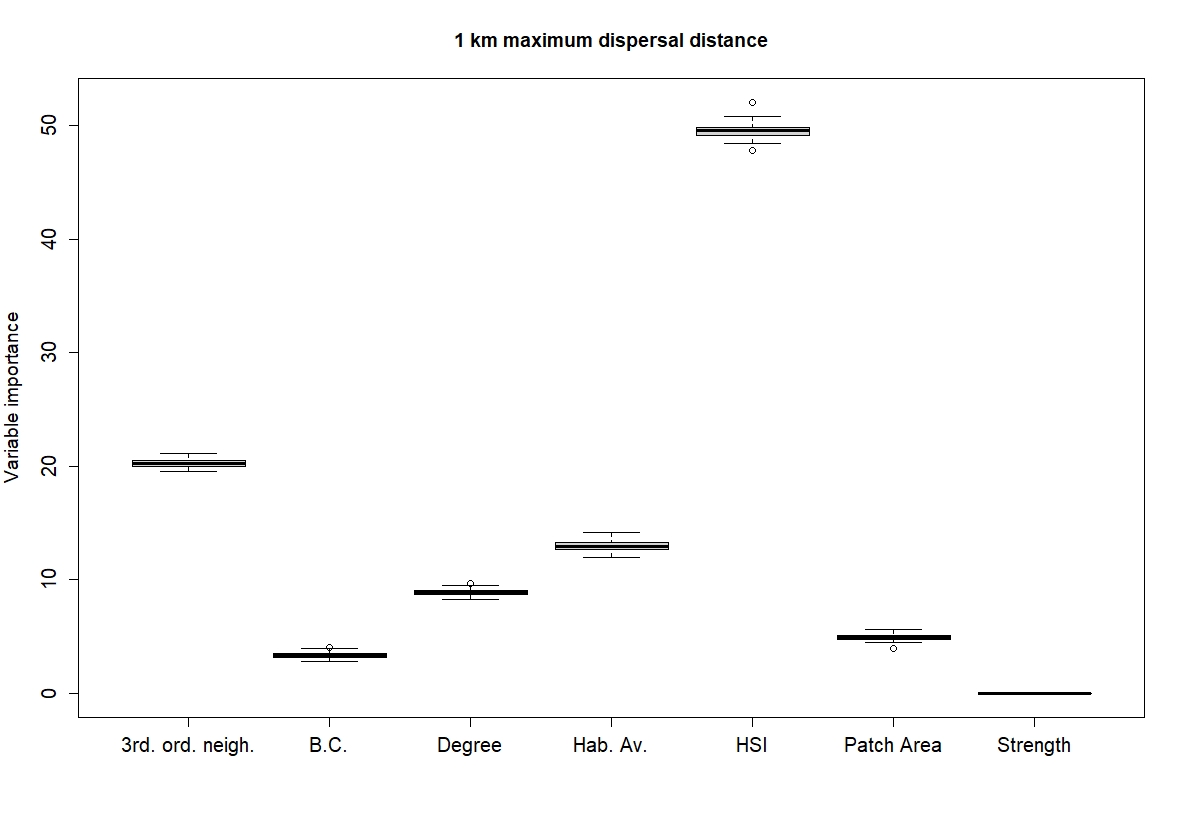


***Pelophylax ridibundus***


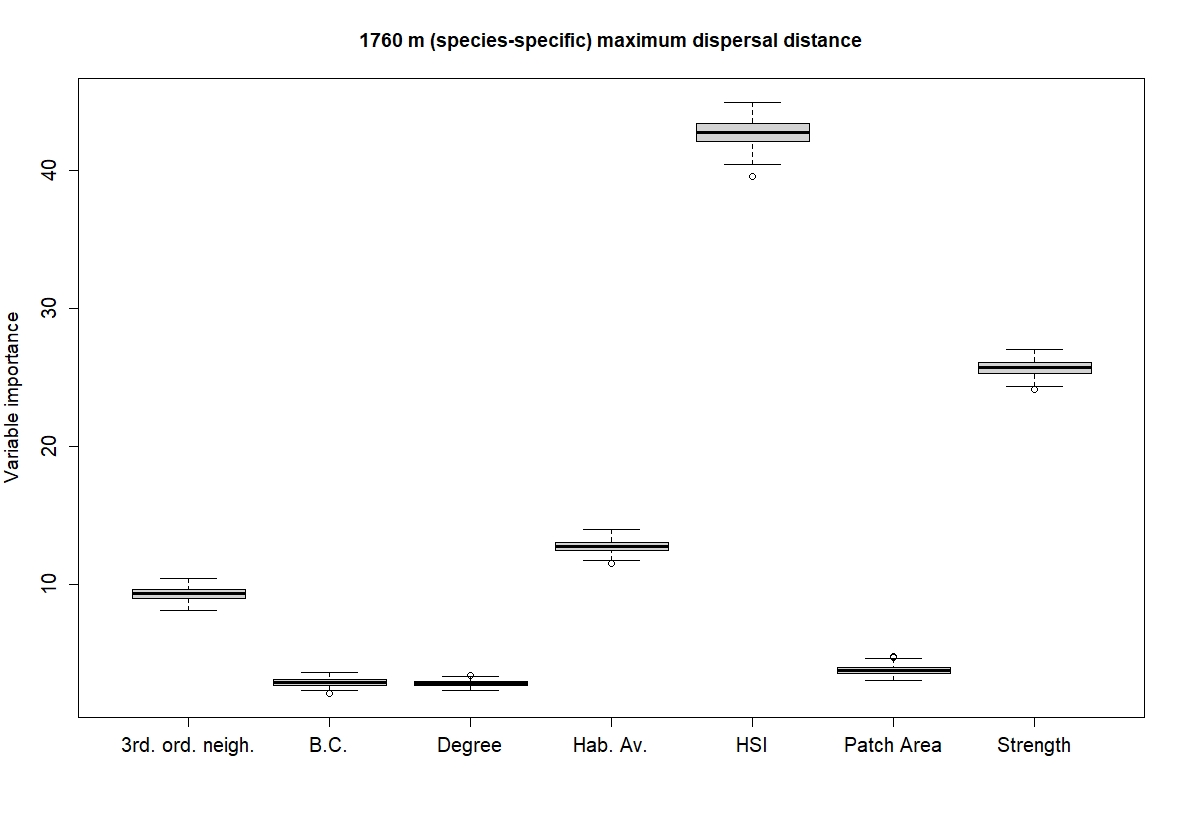

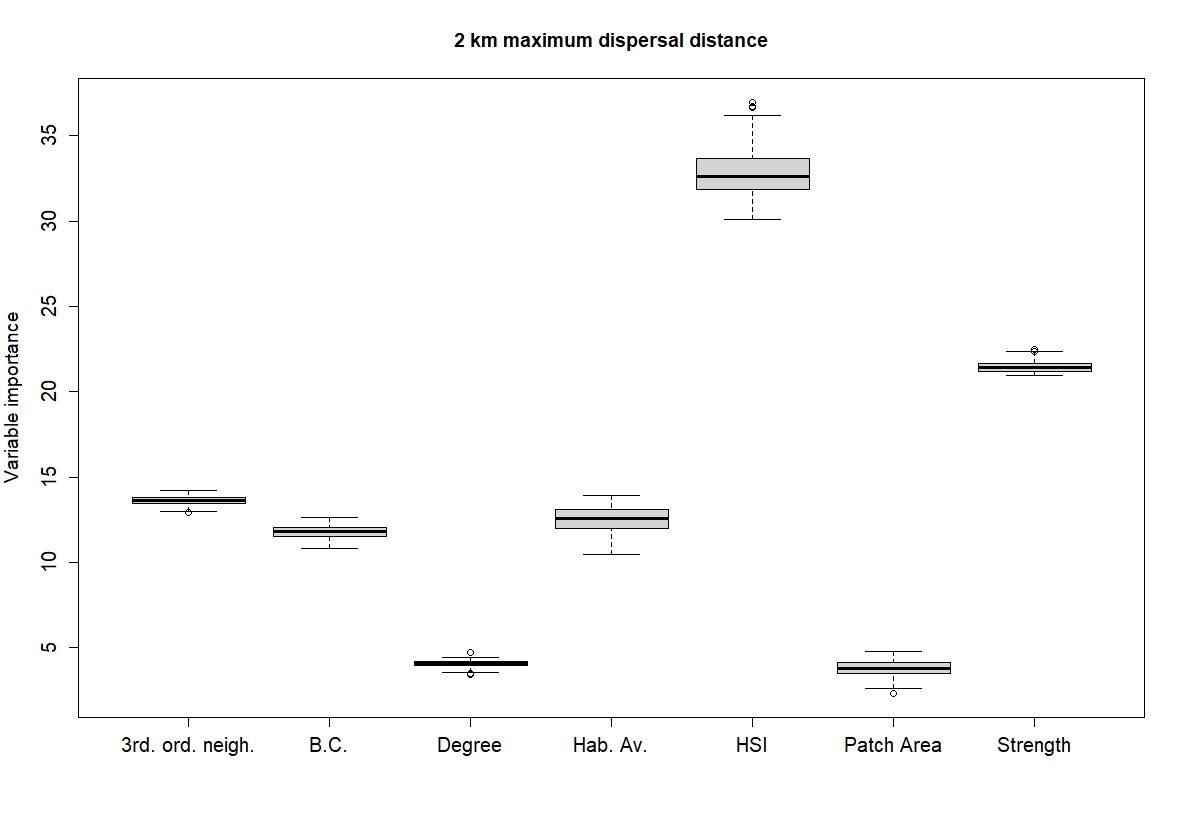


***Pelophylax ridibundus***


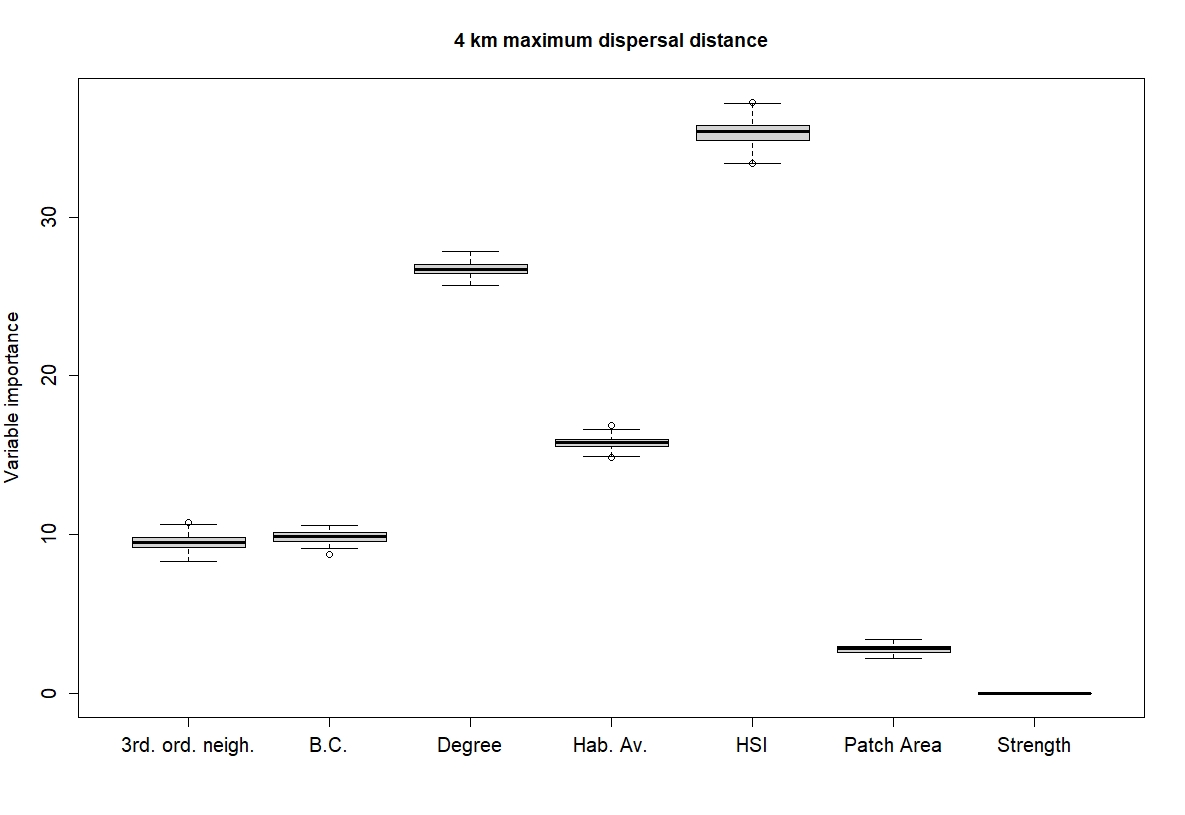

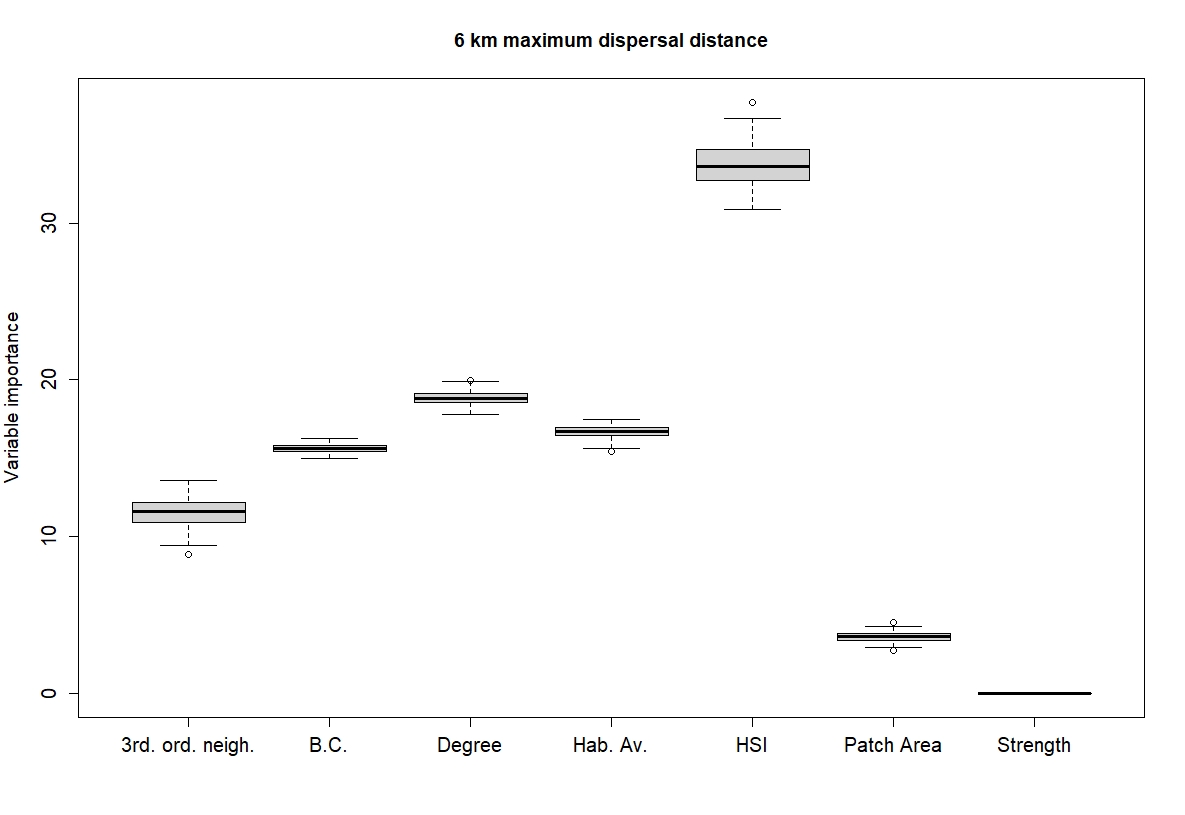


***Pelophylax ridibundus***
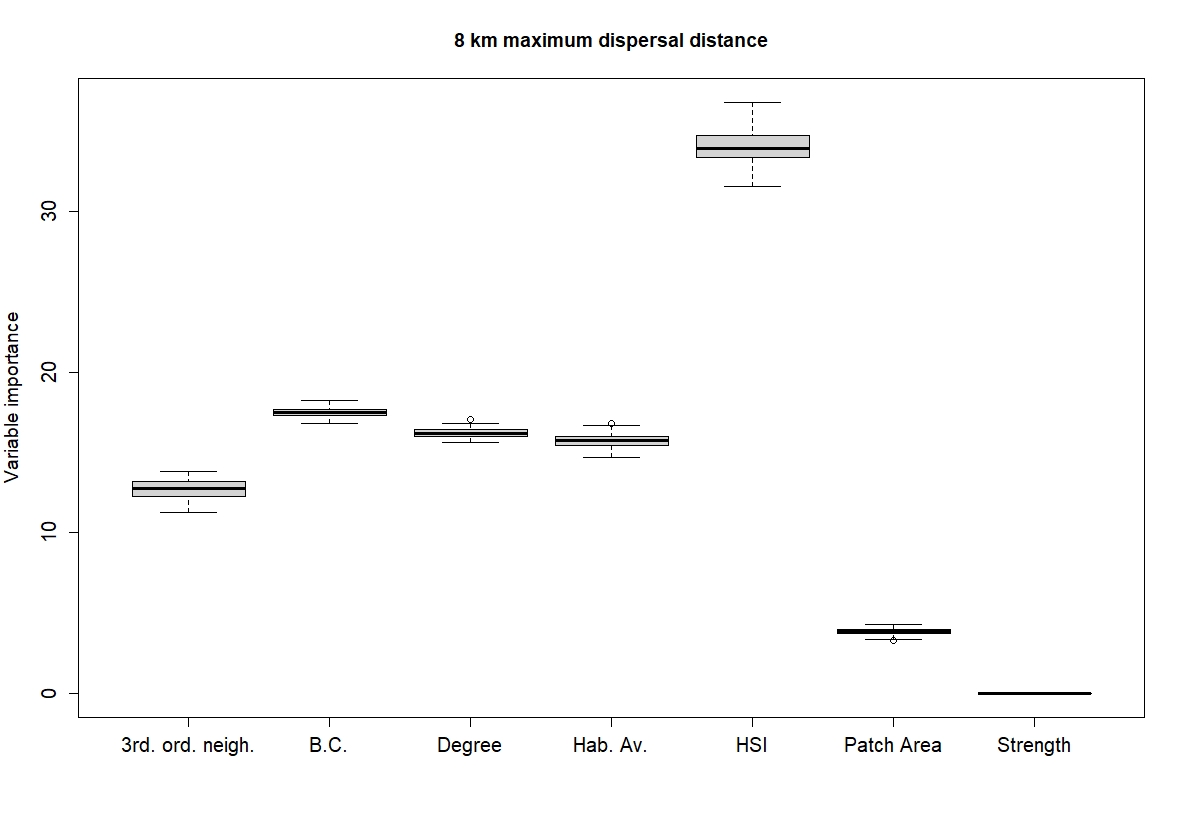

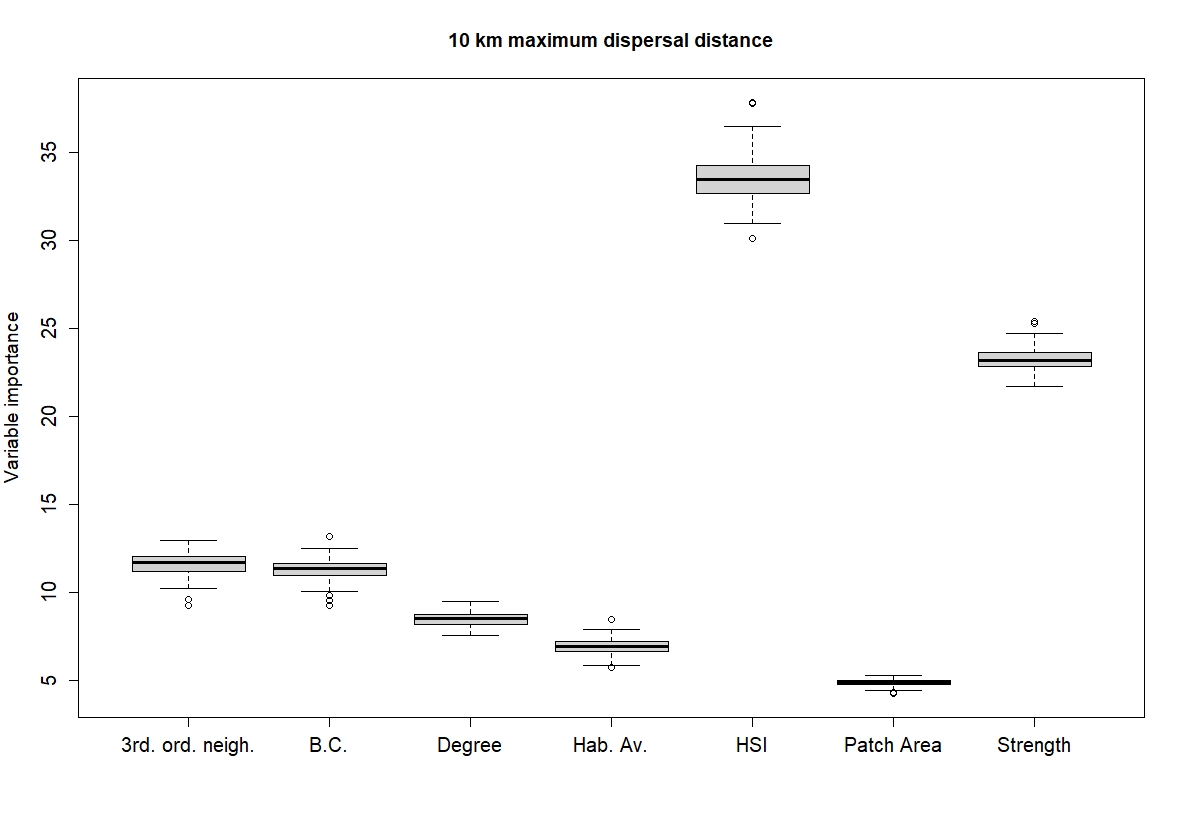

Supplement: S5 Appendix — In each figure: 3rd. org. neigh = third-order neighborhood, B.C. = betweenness centrality, Hab. Av. = habitat availability, HSI = habitat suitability index. (DOCX) [file pone.0293966.s005.docx]
